# Supplementary material for: Protocol for DexEnceph: a randomised controlled trial of dexamethasone therapy in adults with herpes simplex virus encephalitis
Source: BMJ Open. 2021 Jul 21;11(7):e041808. doi: 10.1136/bmjopen-2020-041808 (PMC8728349; doi:10.1136/bmjopen-2020-041808)
Supplement: Supplementary data [file bmjopen-2020-041808supp002.pdf]

RCT Adult with Capacity Information Sheet
Version 5.0, 25 May 2018

DexEnceph: A study of dexamethasone in adults with Herpes Simplex Virus (HSV) encephalitis
Brain Infections Group, University of Liverpool

We understand this is a difficult and stressful time for you, so we firstly want to thank you for taking the time to read this leaflet.

You are being invited to take part in a research study on HSV encephalitis. This condition is extremely rare and is probably something you had never heard about before. This is why a team member will go through this leaflet with you, explaining what taking part in the study would involve and answering any questions.

Important things you need to know

- This is a study for patients with encephalitis (swelling of the brain) caused by a virus called herpes simplex virus (HSV).
- Encephalitis can make you confused, drowsy, behave out of character, affect your sleep and memory, change your mood or may cause you to have fits.
- We want to find out if reducing the swelling with a drug called dexamethasone is of benefit to patient’s memory in the longer term.
- In the study there will be two groups of patients, one that receives dexamethasone and one that does not.
- If you are in the group that receives dexamethasone this will be for 4 days in hospital.
- Both groups will have the same investigations to see if dexamethasone has been of benefit.
- Dexamethasone is a commonly used drug in brain swelling and many other conditions. Like all medicines, dexamethasone has side-effects. We will explain what these can be later.

We would like to invite you to take part in a research study

- Before you decide to take part it is important you know why the research is being done and what it will involve.
- You can discuss with family, friends and clinical staff before making a decision.
- You are free to decide whether you would like to take part.
- If you choose to take part and then decide you no longer want to be involved you can stop taking part without giving a reason. Your care will not be affected.
- Please let us know if there is anything in this leaflet that is not clear or if you would like more information. A member of our team will answer your questions.
- If you decide to take part we will offer you a copy of this form and ask you to sign a consent form.

Whitfield T, *et al.* *BMJ Open* 2021; 11:e041808. doi: 10.1136/bmjopen-2020-041808

HSV encephalitis

1. What is HSV encephalitis?

Encephalitis means swelling of the brain and has many different causes. It is often caused by a virus. Herpes Simplex Virus (HSV) is the most common virus that causes encephalitis in the UK.

HSV encephalitis is very rare. It is diagnosed by finding the virus in fluid around the brain and spinal cord. This fluid is called CSF (cerebrospinal fluid). The CSF is obtained by the doctor who performs a lumbar puncture (LP).

HSV encephalitis is treated with the drug aciclovir. Despite treatment, some people are left with significant loss of memory. About 2 out of every 3 people will have memory difficulties long term.

The study

2. Why are we doing this study?

We know dexamethasone can reduce swelling. Reduction in swelling of the brain may improve the recovery of patients with HSV encephalitis.

This study, called DexEnceph, will allow us to compare the recovery of patients that received dexamethasone and those that did not.

3. Why have I been invited to take part?

There are two reasons why you may have been invited to take part:

A. Your doctors have diagnosed you with having HSV encephalitis.

OR

B. You may have been invited to take part before the diagnosis is made. This is because your doctors think there is a chance you may have HSV encephalitis. This will mean you have more time to think about taking part.

4. What will happen to me during the study?

All patients in the study will receive aciclovir. This is standard treatment for HSV encephalitis.

In addition, if you decide to take part in the study, you may be offered a short course of dexamethasone. This will be decided at random by a computer. This is to be fair, so neither you, your doctor, nor the research team, can choose whether you receive dexamethasone or not. Half of the people in the study will receive dexamethasone and half will not.

If you receive dexamethasone this will be 4 times a day for 4 days. It is given in a line you already have for clinical care.

What taking part involves

5. What tests are done if I take part?

When you sign up to the study:
Blood sample
CSF sample
MRI scan

↓

After 2 weeks:
MRI scan
Blood sample
CSF sample

↓

After 1 month (or when you are discharged from hospital):
Review of activities daily living
Short memory test only

↓

After 6 months:
Memory test
MRI scan
Blood sample
Review of activities daily living
Health questionnaires

↓

After 18 months:
Memory test
MRI scan
Review of activities daily living
Health questionnaires

All the tests done when you sign up to the study and the CSF tests after 2 weeks will

Whitfield T, et al. BMJ Open 2021; 11:e041808. doi: 10.1136/bmjopen-2020-041808

be done as part of your care whether you take part in the study or not.

6. What do the memory tests involve?

These tests are the most important in the study as they will help us find out if dexamethasone improves memory problems from HSV encephalitis. These tests are sometimes called Neuropsychology tests. They are completed 6 and 18 months after the illness.

The key part of this test takes about 35 minutes. If you are not too tired we can continue with further tests that will provide useful information. These can take up to 2 hours.

They are not pass or fail tests. They provide information about your memory and thinking processes.

They can be done in one day or divided over a few short visits. If you have left hospital we can travel to see you in a convenient place for you. The test will be arranged on a day(s) which suits you.

The results can be added to your hospital notes for future reference if you wish or kept confidential within the trial.

7. What does the MRI scan involve?

As part of your care your doctor will organise an MRI scan when you are in hospital. If you take part in the study we will ask you to have another 3 scans later on.

MRI scans allow us to assess if the brain has been affected by the infection and, if so, which parts.

Each scan takes about 20 minutes. The scan can be noisy but you will be offered headphones.

The extra 3 scans are planned for:

- <u>2 weeks</u> after the first one (when you are still in hospital)
- <u>After 6 months</u>
- <u>After 18 months</u>

The scans will be done at a hospital near you. We will reimburse mileage or public transport costs for any research visits.

We will check with you that you are still happy to have the scan each time. Sometimes scans may find something not related to this illness. If this happens the doctors looking at the scans will tell your own hospital doctors who will look into this further.

None of the research scans are compulsory so if you do not wish to have them you can still be part of the study.

8. Are there risks to having an MRI scan?

There are no known risks from an MRI scan. They do not use radiation. MRI scans are done routinely in patients with HSV encephalitis.

Because MRI scans use strong magnets you will not have the scan if you have any metal implants or fragments in your body.

Where you lie is quite enclosed and some people may find this unsettling. If you have a fear of confined spaces you should discuss this with your doctor before you go for the scan.

If you think you may be pregnant let your local research team know. We will not ask pregnant women to have MRI scans due to possible risks to the foetus.

9. What samples are collected? What does this involve?

We will collect blood and CSF samples during the study.

All patients with HSV encephalitis need a lumbar puncture (LP) when they come to hospital to find out why they are unwell. The doctor uses a small needle to take a sample from the lower part of the back. This is repeated after 2 weeks of treatment to see if all the virus has gone. Both lumbar punctures are part of the standard care in all patients with this condition.

We will take a little extra fluid at this time for the research tests. The amount of fluid we ask for each time is about 1 teaspoon, 5.5mls.

If you have already had a lumbar puncture before being told about the study, we will take stored CSF that is leftover for research tests.

Blood tests are requested at 3 different times spread over 6 months. We take between 1 to 4 teaspoons of blood, this is 5 to 23mls.

With these blood and CSF tests we will be able to better understand how the infection affects your body and how the body tries to defend itself against it.

10. What will happen to the samples that are collected? Will any genetic tests be done?

All samples will be taken at your hospital and then transported to the University of Liverpool or other laboratories supporting the study. The samples will not have any of your personal information written on them. In the University they will be stored in a secure building.

There is an option for the blood and CSF collected to have tests looking at DNA. DNA is found in all cells of the body and contains the genetic information for the working of all human beings. This study collects DNA samples to find out why some people get HSV encephalitis and others do not, and why some people have severe problems due to HSV and others do not. The information we learn from DNA may benefit others with this condition in the future but will not influence your treatment or your future health.

Some of your samples may be left over. We will ask you if they can be used for this and future studies run by the University of Liverpool.

11. How do you review activities of daily living?

We will find out how the illness has affected your day-to-day life.

The research team will look at your hospital notes. They may also talk to you and, if you choose, your relatives. This will happen when you are in hospital and when you have gone home.

We will compare patients who received dexamethasone to those that did not and see if it made a difference.

12. What are the health questionnaires?

Two questionnaires will be sent through the post. They will ask you your views about your health and quality of life. Please send them back in a pre-paid envelope.

Dexamethasone

13. What are the side effects of dexamethasone?

Dexamethasone is used widely in patients and the side-effects are well known as this medicine has been prescribed for a long time. A short course of dexamethasone will be prescribed in this study. Side effects are less common when dexamethasone is given for shorter periods.

It is important you know about the possible side-effects before you decide to take part. These are:

- Stomach pain, indigestion, having more appetite than usual, feeling or being sick.
- Feeling tired or fatigued
- Mood and behaviour changes, especially at the beginning.
- Higher blood sugars.

Other possible risks can include:

- Stomach ulcers and bleeding of ulcers.
- Decreased response to infections.

You will be in hospital when you take dexamethasone so you can tell your doctors immediately if you have any problem.

If you suffer side effects you or your doctor can decide to stop the dexamethasone at any point.

Dexamethasone is prescribed to women who are pregnant or breast feeding as there are no known risks to the foetus.

Other things to consider

14. Do I have to take part?

No, taking part is voluntary. If you agree, we will ask you to sign a consent form.

If you agree to take part you are free to change your mind at any time, without giving a reason. You may decide to have only some tests in the study without having to drop out of the study altogether. This will not affect the standard of care you receive.

If you withdraw from the study we will stop collecting data. We will ask you if we can use the information and samples we have gathered up to the point that you withdraw.

15. What happens if there is a problem?

If you have any concerns about any part of this study, please speak with your hospital doctor (consultant) or one of your research team.

If you remain unhappy and wish to complain formally you can do this using the NHS Complaints Procedure. You can get information on how to do this from the Patients Advice Liaison department (PALS) in your hospital.

If you suffer harm from taking part in this study, there are no special compensation arrangements. If harm occurs to you and it is due to someone's negligence, you may have grounds for legal action for compensation against the NHS hospital where you are being treated but you may have to pay your legal costs.

16. Who will know I have taken part in this study?

Only people in your clinical care team and people involved in the study will have access to personal data. With your consent we will tell your GP that you are taking part.

All information collected about you during this study will be confidential and anonymised. It will be handled, stored and destroyed in accordance with the General Data Protection Regulation.

University of Liverpool is the sponsor for this study based in the United Kingdom. We will be using information from you and your medical records in order to undertake this study and will act as the data controller for this study. This means that we are responsible for looking after your information and using it properly. University of Liverpool will keep identifiable information about you for 15 years after the study has finished

Your rights to access, change or move your information are limited, as we need to manage your information in specific ways in order for the research to be reliable and accurate. If you withdraw from the study, we will keep the information about you that we have already obtained. To safeguard your rights, we will use the minimum personally-identifiable information possible.

You can find out more about how we use your information http://www.dexenceph.org.uk/. Our Data Protection Officer is Victoria Heath and you can contact them at V.Heath@liverpool.ac.uk.

Benefits and risks

17. What are the benefits of taking part?

You may benefit from receiving dexamethasone, however we will not know this until the end of the study. You

Whitfield T, et al. BMJ Open 2021; 11:e041808. doi: 10.1136/bmjopen-2020-041808

may also benefit from the increased monitoring of having extra scans and memory tests.

The information we get from this study may benefit patients in the future.

18. What are the possible disadvantages and risks of taking part?

The disadvantage in taking part in this study may be the risk of having the side-effects of dexamethasone listed in question 13 (this will not be the case if you are in the group that does not have dexamethasone).

There is the inconvenience of having the dexamethasone through the drip when you are in hospital. Once you leave hospital there is the inconvenience of travelling to hospital for 2 scans, having the memory tests and completing questionnaires.

Contact details

If you have any questions about this study, then please contact the study team members:

Principal Investigator (Doctor leading this study in your hospital):

Name: ______________________________

____

Telephone:__________________________

Research Nurse:

Name: ______________________________

____

Telephone:__________________________

Name of your Hospital:________________

____

Further information

This study is being run at your hospital and many other NHS hospitals throughout the UK. It aims to recruit 90 patients over 4 years.

It is organised by the University of Liverpool and is funded by the National Institute for Health Research (NIHR), the public body in charge of research in the UK.

Our study team includes The Encephalitis Society, a charity that supports patients and families (www.encephalitis.info).

The study has been reviewed for scientific content by expert members of NIHR. The National Research Ethics Service Committee Liverpool Central has reviewed the study and given approval for it to take place.

Whitfield T, *et al. BMJ Open* 2021; 11:e041808. doi: 10.1136/bmjopen-2020-041808

RCT Adult with Capacity Consent Form
Version 5.0, Dated: 25/May/2018
EudraCT Number: 2015-001609-16

Centre Name: Centre Code:
Name of Principal Investigator:
Study Number:

Please complete this form. When completed give one copy to the participant to keep, send one copy to CTU [fax/encrypted email/post], and keep one in the participant's medical notes. Please put the original in the site file.

For patient: once you have understood each statement please initial the YES OR NO box	YES	NO
1. I confirm I have read and understand the Information Leaflet (dated DD/MMM/YYYY) for the above study, and have had the opportunity to ask questions and have these answered satisfactorily.	INITIAL IF YES	INITIAL IF NO
2. I agree to take part in this study.	INITIAL IF YES	INITIAL IF NO
3. I understand that my participation is voluntary and that I am free to withdraw from the study at any time without giving a reason and without my care or legal rights being affected.	INITIAL IF YES	INITIAL IF NO
4. I agree for my consent form and contact details to be passed to the University of Liverpool for the administration of the study.	INITIAL IF YES	INITIAL IF NO
5. I understand that relevant sections of my medical notes and any data collected during the study may be looked at by authorised individuals from the research and clinical team and Regulatory Authorities where it is relevant to my taking part in this research. I give permission for these individuals to have access to my records.	INITIAL IF YES	INITIAL IF NO
6. I agree for genetic tests to be done on blood and CSF collected. I understand these genetic tests will not be of any individual significance to me.	INITIAL IF YES	INITIAL IF NO

Whitfield T, *et al. BMJ Open* 2021; 11:e041808. doi: 10.1136/bmjopen-2020-041808

UNIVERSITY OF LIVERPOOL
NHS National Institute for Health Research

7. I agree to have MRI scans as part of the trial.	INITIAL IF YES	INITIAL IF NO
8. I agree for my GP and hospital doctors to be informed if the scan picks up something unexpected.	INITIAL IF YES	INITIAL IF NO
9. I agree to gift the remainder of any blood or CSF sample to the University of Liverpool where it will be stored for use in future research. This may include genetic tests.	INITIAL IF YES	INITIAL IF NO
10. I agree to any images or scans that are taken to be used for teaching, education and publication (in scientific journals, books or internet).	INITIAL IF YES	INITIAL IF NO
11. I agree for my GP to be informed I am taking part in this study.	INITIAL IF YES	INITIAL IF NO

Name of Participant (Please print)	Signature	Date (DD/MM/YYYY)
Researcher*	Signature	Date (DD/MM/YYYY)

*** Important:** Prior to signing please ensure local research contact details are complete on page 6.

<u>Information to Research Team:</u>
Once a Consent Form has been signed, please copy three times: One for the participant, one to file in the medical notes and fax/post/encrypted email one to CTU. Please place original in the site file.

Please fax/encrypt email/post this consent form to CTU **separately** to other anonymised trial documents (e.g. CRF).
