# Supplementary material for: Protocol for DexEnceph: a randomised controlled trial of dexamethasone therapy in adults with herpes simplex virus encephalitis
Source: BMJ Open. 2021 Jul 21;11(7):e041808. doi: 10.1136/bmjopen-2020-041808 (PMC8728349; doi:10.1136/bmjopen-2020-041808)
Supplement: Author's manuscript [file bmjopen-2020-041808.draft_revisions.pdf]

BMJ Open is committed to open peer review. As part of this commitment we make the peer review history of every article we publish publicly available.

When an article is published we post the peer reviewers' comments and the authors' responses online. We also post the versions of the paper that were used during peer review. These are the versions that the peer review comments apply to.

The versions of the paper that follow are the versions that were submitted during the peer review process. They are not the versions of record or the final published versions. They should not be cited or distributed as the published version of this manuscript.

BMJ Open is an open access journal and the full, final, typeset and author-corrected version of record of the manuscript is available on our site with no access controls, subscription charges or pay-per-view fees (http://bmjopen.bmj.com).

If you have any questions on BMJ Open's open peer review process please email info.bmjopen@bmj.com

BMJ Open

Protocol for DexEnceph; a randomised controlled trial of dexamethasone therapy in adults with Herpes Simplex Virus Encephalitis

Journal:	*BMJ Open*
Manuscript ID	bmjopen-2020-041808
Article Type:	Protocol
Date Submitted by the Author:	17-Jun-2020
Complete List of Authors:	Whitfield, Thomas; University of Liverpool, Brain Infections Unit<br>Fernandez, Cristina; University of Liverpool, Brain Infections Unit<br>Defres, Sylviane; Institute of Infection and Global Health, Clinical infection, medical mircobiology and Immunology; Royal Liverpool and Broadgreen University Hospitals Trust, Tropical infectious diseases Unit<br>Griffiths, Michael; University of Liverpool, Brain Infections Unit; Alder Hey Children's NHS Foundation Trust, Neurology<br>Hooper, Cory; University of Liverpool, Brain Infections Unit<br>Tangney, Rebecca; Aintree University Hospitals NHS Foundation Trust<br>Davies, Kelly; University of Liverpool, Clinical Trials Research Centre<br>Burnside, Girvan; University of Liverpool Faculty of Health and Life Sciences, Biostatistics<br>Rosala- Hallas, Anna; University of Liverpool Faculty of Health and Life Sciences, Biostatistics<br>Moore, Perry; The Walton Centre NHS Foundation Trust, Dept of Clinical Neuropsychology<br>Das, Kumar; The Walton Centre NHS Foundation Trust<br>Zuckerman, Mark; King's College Hospital NHS Foundation Trust, South London Specialist Virology Centre<br>Parkes, Laura; University of Manchester Institute of Science and Technology Department of Biomolecular Science,<br>Keller, Simon; Aintree University Hospitals NHS Foundation Trust<br>Roberts, Neil; The University of Edinburgh The Queen's Medical Research Institute<br>Easton, Ava; The Encephalitis Society<br>Touati, Saber; CHU Grenoble Alpes, Service des Maladies Infectieuses et Tropicales<br>Kneen, Rachel; Alder Hey Children’s NHS Foundation Trust, Department of Neurology,; Institute of Infection and Global Health, Clinical Infection, medical microbiology and Immunology<br>Stahl, JP; Infectious Diseases Department, University of Grenoble<br>Solomon, Tom; Institute of Infection and Global Health, University of Liverpool; National Institute for Health Research Health Protection Research Unit in Emerging and Zoonotic Infections, University of Liverpool
Keywords:	Neurology < INTERNAL MEDICINE, VIROLOGY, NEUROLOGY, Neurological injury < NEUROLOGY

For peer review only - http://bmjopen.bmj.com/site/about/guidelines.xhtml

BMJ

I, the Submitting Author has the right to grant and does grant on behalf of all authors of the Work (as defined in the below author licence), an exclusive licence and/or a non-exclusive licence for contributions from authors who are: i) UK Crown employees; ii) where BMJ has agreed a CC-BY licence shall apply, and/or iii) in accordance with the terms applicable for US Federal Government officers or employees acting as part of their official duties; on a worldwide, perpetual, irrevocable, royalty-free basis to BMJ Publishing Group Ltd ("BMJ") its licensees and where the relevant Journal is co-owned by BMJ to the co-owners of the Journal, to publish the Work in this journal and any other BMJ products and to exploit all rights, as set out in our licence.

The Submitting Author accepts and understands that any supply made under these terms is made by BMJ to the Submitting Author unless you are acting as an employee on behalf of your employer or a postgraduate student of an affiliated institution which is paying any applicable article publishing charge ("APC") for Open Access articles. Where the Submitting Author wishes to make the Work available on an Open Access basis (and intends to pay the relevant APC), the terms of reuse of such Open Access shall be governed by a Creative Commons licence – details of these licences and which Creative Commons licence will apply to this Work are set out in our licence referred to above.

Other than as permitted in any relevant BMJ Author's Self Archiving Policies, I confirm this Work has not been accepted for publication elsewhere, is not being considered for publication elsewhere and does not duplicate material already published. I confirm all authors consent to publication of this Work and authorise the granting of this licence.

For peer review only

Protocol for DexEnceph; a randomised controlled trial of dexamethasone therapy in adults with Herpes Simplex Virus Encephalitis

The study is funded by the National Institute for Health Research's Efficacy and Mechanism Evaluation (EME) programme.

The trial sponsor is the University of Liverpool, who are responsible for all aspects of study design, implementation, analysis and write up (sponsor@liverpool.ac.uk).

For peer review only

For peer review only - http://bmjopen.bmj.com/site/about/guidelines.xhtml

Dr Tom Whitfield
Brain Infections Group
Institute of Global Health
Ronald Ross Building
University of Liverpool
Liverpool
L7 3EA
July 17, 2019

Dear Mr Adrian Aldcroft,

We wish to submit the 'Protocol for the DexEnceph; a randomised controlled trial of dexamethasone therapy in adults with herpes simplex virus encephalitis' for consideration by BMJ open. We confirm that this work is original and has not been published elsewhere, nor is it currently under consideration for publication elsewhere.

In this paper, we outline our design of the DexEnceph trial, which will be the first completed randomised controlled trial of corticosteroids in herpes simplex encephalitis. DexEnceph is designed to answer the longstanding question of whether using steroids as immunomodulation in herpes simplex encephalitis is effective and has the potential to change global practice. We hope it will give insight into treatment options for managing a condition that still has a high mortality rate and commonly leaves sufferers with devastating long-term sequelae.

We believe that this manuscript is appropriate for publication by BMJ open because in addition to its relevance to clinical practice, the protocol also outlines how the trial is uniquely and pragmatically designed to counter logistic difficulties of recruiting participants who present acutely with a very rare disease sporadic disease. This is of use and interest to other researchers in less common acute conditions. The manuscript is 5000 words in length, in order to cover both the SPIRIT checklist and the additional unique features of the trial. The trial is currently in progress and has recruited over 75% of trial participants, this adds legitimacy to the approach taken where previous trials have been unsuccessful.

Please address all correspondence concerning this manuscript to me at tw1@liverpool.ac.uk.

Thank you for your consideration of this manuscript.

Sincerely,

Tom Whitfield

For peer review only - http://bmjopen.bmj.com/site/about/guidelines.xhtml

Article Summary

Strengths and weaknesses

'Strengths and limitations of this study', and containing up to five short bullet points, no longer than one sentence each, that relate specifically to the methods.

- DexEnceph will be the first completed randomised controlled trial of steroid in herpes simplex virus encephalitis, examining the utility and safety of steroid use.

- The trial will provide important information to improve outcomes in a devastating disease with a high rates of mortality and severe sequalae.

- Herpes simplex virus encephalitis is a rare sporadic disease which presents acutely, with low numbers of expected patients (approximately 1-2 per year) at each recruiting hospital. Screening and site engagement are key to ensuring trial success.

- The pragmatic open label, observer blind design of the trial is ensuring successful recruitment to date.

- The recruitment target is informed by the recent Enceph-UK programme grant of encephalitis in the UK; the trial is currently open and has recruited 71 patients of a target 90.

For peer review only

Abstract

Introduction

Herpes simplex virus (HSV) encephalitis is a severe form of brain inflammation that commonly leaves survivors and their families with devastating long-term consequences. The virus particularly targets the temporal lobe of the brain causing debilitating memory problems, especially in verbal memory. It is postulated that immunomodulation with steroids could improve outcomes by reducing brain swelling. However, there are concerns (so far not observed) that such immunosuppression might facilitate increased viral replication with resultant worsening of disease. A previous trail closed early because of slow recruitment.

Method

DexEnceph is a pragmatic multicentre, randomised, controlled, open label, observer-blind trial to determine whether adults who receive dexamethasone alongside standard treatment with aciclovir for HSV encephalitis have improved clinical outcomes compared with those who receive standard treatment alone. Overall, 90 patients with HSV encephalitis will be recruited from a target of 90 recruiting sites; patients will be randomised 1:1 to the dexamethasone or control arms of the study. The primary outcome measured is verbal memory as assessed by the Weschler Memory Scale fourth edition Auditory Memory Index at 26 weeks post randomisation. Secondary outcomes are measured up to 72 weeks include additional neuropsychological, clinical and functional outcomes as well as comparison of neuroimaging findings. Patient safety monitoring occurs throughout and includes the detection of HSV DNA in cerebrospinal fluid 2 weeks after randomisation, which is indicative of ongoing viral replication.

For peer review only

For peer review only - http://bmjopen.bmj.com/site/about/guidelines.xhtml

Discussion

DexEnceph aims to be the first completed randomised controlled trial of steroid therapy in HSV encephalitis. The results will provide evidence for future practice in managing adults with the condition and has the potential to improve outcomes in a life-changing disease.

Ethical approval

The trial has ethical approval from the UK National Research Ethics committee (Liverpool Central, REF: 15/NW/0545, 10/08/2015). Protocol version 2.1 July 2019

Registration numbers

ISRCTN11774734, EUDRACT 2015-001609-16

Key words: Herpes simplex, Encephalitis, Dexamethasone, Steroid, Verbal memory

Authors: Whitfield T,[1] Fernandez C, [1] Davies K, [2] Defres S[1, 13, 15], Griffiths M, [1, 14] Hooper C,[1] Tangney R, [3] Burnside G, [4] Rosalas-Hallas A,[4] Moore P, [12] Das K, [5] Zuckerman M, [6] Parkes L, [7], Keller S, [3] Roberts N,[8] Easton A, [1,9] Touati S,[10] Kneen R, [1,14] JP Stahl[11] & Solomon T[1, 5,15]

1. Institute of Infection and Global Health, University of Liverpool, L69 7BE, United Kingdom
2. Clinical Trials Research Centre, University of Liverpool, L12 2AP, United Kingdom
3. University Hospital Aintree, L9 7AL, United Kingdom

For peer review only - http://bmjopen.bmj.com/site/about/guidelines.xhtml

4. Department of Biostatistics, University of Liverpool, L69 3GL, United Kingdom
5. The Walton Centre NHS Foundation Trust, L9 7JL, United Kingdom
6. South London Specialist Virology Centre, Kings College Hospital NHS Foundation Trust, SE5 9RS, United Kingdom
7. Division of Neuroscience and Experimental Psychology, Faculty of Biology Medicine and Health, University of Manchester, M13 9NT, United Kingdom
8. Queens Medical Research Institute, University of Edinburgh, EH16 4TJ, United Kingdom
9. Encephalitis Society, Malton, North Yorkshire, YO17 7DT, United Kingdom
10. Département de Recherche Clinique (DRC), Grenoble Cedex, France
11. Infectious Diseases Department, University and Hospital Grenoble Alpes, Grenoble Cedex, France
12. Salford Royal NHS Foundation Trust, M6 8HD, United Kingdom
13. Tropical Infectious diseases Unit, Royal Liverpool University Hospital, L7 8XP, United Kingdom
14. Alder Hey NHS Foundation Trust, Liverpool, L14 5AB, United Kingdom
15. NIHR Health Protection Research Unit on Emerging and Zoonotic Infections, Liverpool, L69 7BE, United Kingdom

Correspondence & trial sponser: tsolomon@liverpool.ac.uk

Word Count : 4916 words

For peer review only

Background

Herpes simplex virus infection (HSV) is the most commonly identified viral cause of encephalitis, inflammation and swelling of the brain caused by a virus or the body's immune system, in the UK as in most western industrialised nations.[1–4] The incidence has been estimated at 1 in 250,000-500,000,[2] with evidence this may be higher.[4] Although a rare disease, HSV encephalitis has a disproportionately large impact due to its devastating long term neuro-psychological sequelae . These can have a marked impact on the quality of life of the patient and their family and high health economic and social costs.[5,6]

Since the introduction of the antiviral drug aciclovir in the 1970s the mortality of HSV encephalitis has reduced from around 70% to 5.5-12%.[7–9] However, survivors are commonly left with neurological impairment; less than 20% of patients are able to return to work and 48% are classed as moderate to severely disabled.[10] Even when obvious disabilities have not occurred, families often report personality changes – the person they take home from hospital is simply not the same as the one before the illness. [6,11]

HSV encephalitis can cause a broad range of cognitive impairments, but impaired memory, especially verbal memory, is the most common and likely relates to the viral predilection for the temporal lobe of the brain. [12,13] The verbal memory deficits manifest as difficulties remembering names of objects and people, as well as listening to and recalling spoken information such as conversations. [14,15] In addition to memory problems, difficulties in processing speed, concentration, language and executive function are also common amongst survivors of HSV encephalitis, along with fatigue, poor concentration, anxiety and depression. [7,16,17]

For peer review only - http://bmjopen.bmj.com/site/about/guidelines.xhtml

The pathogenic mechanisms in HSV encephalitis are not fully understood. The evidence suggests that in addition to direct viral pathogenesis, inflammation of the brain in response to the virus is a key component of the disease process. [18–21] This is supported by the observation that in the cerebrospinal fluid (CSF), higher levels of proinflammatory chemokines, especially monocyte chemotactic protein (MCP)-1, interferon γ and interleukin 6 (IL-6) are associated with a worse prognosis.[22,23] Poor prognosis is also associated with the extent of inflammation seen on neuroimaging,[24] and the degree of temporal lobe swelling is correlated with the severity of verbal memory impairment.[25]

Control of the inflammation in HSV encephalitis may improve outcome, as shown in mouse models of the disease. [26–28] Before the availability of aciclovir, corticosteroids were also used routinely as a treatment in humans with HSV encephalitis,[29,30] and more recently both cerebral oedema on imaging and cerebrospinal fluid IL-6 levels were shown to be reduced in patients given corticosteroids.[23] However, because corticosteroids cause immunosuppression which in theory facilitates increased viral replication, their role is uncertain.[31]

In other brain infections, including bacterial meningitis and tuberculous meningitis the benefit of corticosteroids has been demonstrated in large clinical trials.[32] For HSV encephalitis the potential benefit of using corticosteroid as an adjunct to aciclovir therapy has been suggested from small case series and retrospective comparisons, but there has been no prospective randomised study reported. [33–38] One study, the German trial of Aciclovir and Corticosteroids in Herpes simplex virus Encephalitis (the GACHE trial) was stopped early because of poor recruitment rates. [39] However, there is clearly a need for a study to assess this question. The

For peer review only - http://bmjopen.bmj.com/site/about/guidelines.xhtml

DexEnceph Study, a randomised controlled trial of dexamethasone in HSV encephalitis, aims to achieve this.

Trial design

DexEnceph is a pragmatic, multicentre, randomised, controlled, observer-blind trial to determine whether the addition of dexamethasone to standard aciclovir treatment improves clinical outcomes (in particular verbal memory score) for adults with HSV encephalitis. Additionally, neuroimaging and biomarkers will be assessed along with detection of HSV in the cerebrospinal fluid at 2 weeks post randomisation to monitor for difference in viral replication between the two groups.

Primary objective

To determine whether a short course of intravenous dexamethasone, in addition to standard care, improves verbal memory score in adults with HSV encephalitis at 26 weeks post treatment compared to standard care alone.

Secondary objectives

Secondary objectives include the following:

To determine whether dexamethasone therapy has an effect on other neuropsychological, cognitive, clinical, disability and functional outcomes in HSV encephalitis.

For peer review only - http://bmjopen.bmj.com/site/about/guidelines.xhtml

To assess the effect of dexamethasone therapy on brain swelling assessed by neuroimaging imaging.

To determine whether dexamethasone therapy affects clearance of HSV from cerebrospinal fluid, the emergence of anti-N-Methyl-D Aspartic acid (NMDA) receptor antibody or causes any changes in transcriptomic and proteomic profiling in the CSF and blood.

A more comprehensive list of measures is detailed in the outcomes section.

Methods

DexEnceph is an observer blind, open label, prospective, randomised, controlled trial of dexamethasone at 10mg four times daily for 4 days, versus no dexamethasone, in adults with HSV encephalitis.

Research setting

The trial is being conducted in up to 45 NHS trusts, with a recruitment target of 0-2 patients per site per year. A full list of sites involved in DexEnceph can be obtained from www.dexenceph.org.uk

Eligibility criteria

Inclusion Criteria

Enrolled patients fulfil all of the following criteria:

1. Suspected encephalitis defined as: new onset seizure OR new focal neurological signs OR alteration in consciousness, cognition, personality, or behaviour. Personality / behaviour

For peer review only

change includes agitation, psychosis, somnolence, insomnia, catatonia, mood lability, altered sleep pattern.

2. A positive HSV DNA polymerase chain reaction (PCR) result from CSF, reported not more than 7 days prior to randomisation.

3. Receiving intravenous aciclovir administered as 10mg/kg three times daily or at a reduced dose if clinically indicated.

4. Age ≥ 16 years.

5. Written informed consent given by the patient or their legal representative[40]

Exclusion Criteria

Patients are excluded if they have any of the following:

1. Have received oral or injectable corticosteroid therapy in the 30 days prior to the day of admission to hospital. This does not apply to topical/ inhaled corticosteroids. [Patients who have received oral or injectable corticosteroid therapy AFTER their admission to hospital will not be excluded from the study if they consent to trial participation].

2. History of hypersensitivity to corticosteroids.

3. Immunosuppression secondary to:

For peer review only

For peer review only - http://bmjopen.bmj.com/site/about/guidelines.xhtml

a. Known HIV infection and CD4 white cell count under 200/mm^3

b. Currently taking biologic therapy or other immunosuppressive agents [e.g. azathioprine, methotrexate, ciclosporin]

c. Previous solid organ transplant and currently on immunosuppression

d. Previous bone marrow transplant

e. Currently undergoing a course of chemotherapy or radiotherapy

f. Known primary immunodeficiency syndrome

g. Known current haematological malignancy

4. Pre-existing indwelling ventricular devices.

5. Peptic ulcer disease in the last 6 months, defined as a peptic ulcer seen at endoscopy or an upper gastrointestinal bleed causing a ≥ 2unit haemoglobin drop, in the last 6 months.

6. Antiretroviral regime containing rilpivirine as current treatment [Levels of rilpivirne are known to significantly decrease in co-administration with dexamethasone, a switch to a suitable alternative can facilitate trial entry].

Intervention

Participants are randomised in a 1:1 ratio to dexamethasone four times daily for four days alongside standard care, or standard care alone (Figure 1). Standard care includes intravenous aciclovir for a minimum of 14 days based on an ideal body weight at 10mg/kg every 8 hours, unless dose adjustment to account for renal impairment is necessary. Participating clinicians

For peer review only

remain free to modify or discontinue the dexamethasone administration or to give alternative treatments at any stage, if this is judged to be in the best interest of the patient.

Participants assigned dexamethasone receive 10 mg equivalent of ordinary ward stock, prescribed by an authorised member of the local study team, given intravenously four times daily for four days (16 doses in total) starting within 24 hours of randomisation.

The University of Liverpool employs a clinical trials unit to be responsible for screening, and monitoring data collection, quality and completeness. As there is a low number of participants to be recruited, the trials unit are able to liaise regularly with each site following randomisation to ensure all follow up data are collected. Primary outcome is recorded by a centrally employed roving neuropsychologist, who collects the neuropsychological outcomes at 26 and 72 weeks.

For peer review only

Outcome measures

Primary Outcome

Verbal memory score, determined by the Wechsler Memory Scale 4th edition (WMS-IV) Auditory Memory Index, at 26 weeks after randomisation.

Secondary Outcomes

Other neuropsychological outcome measures [at 26 weeks and 78 weeks after randomisation]:

- Verbal memory score, determined by the WMS-IV, Auditory Memory Index, at 78 weeks after randomisation.
- Visual, Immediate and Delayed Memory by Indexes of the WMS-IV, processing speed and working memory subscales from the Wechsler Adult Intelligence Scale Fourth Edition.
- Higher executive function using the Trail Making Test.
- Anxiety and Depression symptom levels by the Beck Depression Inventory and Beck Anxiety Inventory.
- Subjective cognitive complaints using the Perceived Deficits Questionnaire.

Cognitive Outcome Measures [at discharge or 30 days if still in hospital, 26 weeks and 78 weeks]

- Addenbrooke's Cognitive Assessment III.

Clinical Outcomes

- Incidence of epilepsy.
- Time to hospital discharge.

For peer review only

- Requirement of high dependency unit or Intensive care unit admission up to 30 days post randomisation.
- Time taken to be free of ventilatory support for 14 days [if any].
- Time to reach maximum recorded Glasgow coma scale score.
- Survival.

Disability & Functional Outcomes [at discharge or 30 days if still in hospital, 26 weeks and 78 weeks]:

- Modified Rankin Score, Barthel Index, Liverpool Outcome Score and Glasgow Outcome Scale Extended.

Imaging Outcomes: Change from Baseline at 2 weeks, 26 weeks and 78 weeks

- Temporal lobe volume (as % of intra-cranial volume).
- Whole brain volume (as % of intra-cranial volume).
- Volume of affected region as seen on fluid-attenuated inversion recovery (FLAIR) image (as % of intra-cranial volume).
- Volume of affected region as seen on diffusion-weighted image (as % of intra-cranial volume).

Biomarker Outcomes:

- Transcriptomic and proteomic profiling on blood at convalescence (2 weeks and 26 weeks), compared to acute baselines, and on CSF at 2 weeks compared to acute baseline.
- Anti-NMDA receptor antibody testing at 26 weeks.

Safety Outcomes:

- Proportion of patients with detectable HSV in CSF by PCR at 2 weeks.

Health Status and Quality of Life [at 26 and 78 weeks]:

For peer review only

- Measured by the EuroQoL-5 Dimension-5 Level quality of life scale (EQ-5D-5L) and 36 item short form survey (SF-36) self-completed questionnaires.

Screening

The majority of potential patients are identified by the local research team through identifying patients with a relevant clinical presentation suspicious of HSV encephalitis and/or detection of HSV in a CSF sample. A screening log is completed for all potential patients. A strong link with the local laboratory is essential as a key factor in ensuring eligible patients are not missed by the investigative team. Investigators for the local research team include neurologists, infectious disease clinicians, microbiologist and virologists.

Because this is an uncommon disease, extra measures have been taken to try and maximise recruitment. On identifying a suitable patient, sites are able to contact the trial management team for intensive support via a dedicated telephone hotline, email, or an app. Short videos which explain the trial to patients, families and to health care workers also support recruitment. Every month, the trial management group monitor the screening reports of each site for the previous 3 months, to ensure they are actively looking for patients. Lower than expected screening is followed up by the central study team making contact with the study site to review their screening methodology and offer support.

Randomisation

Participants are randomised using a 24-hour secure web-based programme, which is centrally controlled by the clinical trials research centre. Designated members of the trial team at the

For peer review only

site (detailed on the delegation of responsibilities log) are provided with a unique username and password which is required to access the web-based randomisation system. In the event of system failure, the patient can be randomised centrally electronically or through secure envelopes. Each participant is allocated a unique study number (randomisation number), the primary identifier for all the participants in this study.

The neuropsychologist collecting the primary outcome and other outcome assessors such as radiologists and those responsible for paper authorship are blinded to randomisation during the trial. Trial participants and local site study teams, as well as the trial manager and trial data manager at the clinical trials unit are aware of what treatments have been allocated. The independent data safety and monitoring committee (IDSMC) and statisticians have access to unblinded data grouped by intervention throughout the trial and make recommendations to the trial's steering committee who would only become unblinded in the event of a serious event.

For peer review only

Participant timeline

The time schedule for enrolment, interventions and assessments is given in table 1.

Statistical Considerations

Sample Size

The primary outcome variable is verbal memory , assessed as part of WMS-IV. In one published series of adults who survived HSV encephalitis, 19 of 22 had memory impairment

For peer review only - http://bmjopen.bmj.com/site/about/guidelines.xhtml

evident at follow up, with verbal memory being most severely affected [12]. In that study the mean (standard deviation, SD) verbal memory score was 88.9 (18.9) compared with the population mean (SD) of 100 [15]. This score can only be assessed in survivors, we estimate approximately 10% of patients in the trial will die before assessment of the primary outcome[8,41,42]. In instances where the death is judged to be associated with encephalitis the verbal memory score is recorded as 40, (the lowest possible value which would be obtained even where a patient recorded no recall of any of the items administered in the memory subtests). Where the cause of death is thought to be independent of having encephalitis, those patients will be recorded as lost to follow up. Similarly, for patients who are too unwell, due to encephalitis, to undergo the assessment, the score is recorded as 40. Decisions as to whether the reasons for death or non-completion of the measures were due to encephalitis will be made by an independent committee blinded to dexamethasone allocation. Adjusting the estimate of mean and standard deviation from survivors, to include the 10% of patients with the lowest possible value of 40, gives a total population mean of 84.8, with a standard deviation of 23.1. A final sample size of 36 participants per group allows us to detect a clinically meaningful difference of 15.5 on the verbal memory score with 80% power, at a two-sided significance level of 0.05. Allowing for up to 20% dropout gives an initial target sample size of 45 participants per group, for a total of 90.

Statistical analysis

For the primary outcome, participants are included in the analysis based on the intention-to-treat principle. Verbal memory score will be compared between groups using linear regression. The model will be adjusted for pre-specified variables which are judged to be

For peer review only

potentially related to the outcome, including age and admission Glasgow coma scale score. No interim analysis is planned, but there is regular monitoring by the IDSMC.

As there may be some missing primary outcome data due to death, inability to complete the assessment, or loss to follow up, a sensitivity analysis will be carried out. All randomised patients will be included in this analysis.

For continuous secondary outcome variables, comparisons between groups will be analysed as per the primary outcome. The results for residual viral presence in the CSF at 2 weeks will be reported with a 95% confidence interval for the difference in proportions between groups. Time to event outcomes will be analysed using Kaplan-Meier curves, log rank tests and Cox Proportional Hazards models. Binary secondary outcomes will be analysed using logistic regression.

Trial Promotion and engagement

Considerable effort is maintained to keep the principal investigators, research nurses and the community engaged in the trial. The trial is being publicised using public forums, the Encephalitis Society website and newsletter, social media, patient journey articles and work with newspapers and TV. To promote site engagement, study days are arranged for research teams to attend, along with scheduled research nurse teleconferences to allow ideas on maximising recruitment and updates on trial progress to be shared. Sites are also kept updated through our website (www.dexenceph.org.uk), newsletters and an innovative sticker chart, whereby a sticker is sent out to every site each time a patient is recruited (Figure 2).

For peer review only

The Encephalitis Society are playing a key role in providing additional support to the patients and their families aside from their work for the trial.

Publication and Dissemination

The results from different centres will be pooled for analysis and published as soon as the analysis is complete. They will be presented at the annual conference organised by the Encephalitis Society and at other meetings.

For peer review only

Trial Closure

The end of the trial is defined to be the date on which data for all participants is frozen and data entry privileges are withdrawn from the trial database. The trial may be closed prematurely by the trial steering committee, on the recommendation of the IDSMC if there is sufficient evidence of risk to patient safety.

Pharmacovigilance

Oversight of the trial is provided by the trial steering committee, which meets at least annually to review trial progress, safety, and adverse events. The committee is also informed of any protocol changes by the clinical trial research unit.

The Medicines for Human Use (Clinical Trials) Regulations 2004 (SI 2004/1031) definitions are used for Adverse Event (AE), Adverse Reaction (AR), Unexpected Adverse Reaction (UAR), Serious Adverse Event (SAE), Serious Adverse Reaction (SAR) or Suspected Unexpected Serious Adverse Reaction (SUSAR).

Depending on the nature of the event the reporting procedures below are followed:

For peer review only - http://bmjopen.bmj.com/site/about/guidelines.xhtml

1. Serious adverse events occurring up to 30 days after randomisation are reported through a Serious Adverse Event Form (if serious) or in the 30 day/discharge case record forms (CRF) if they are a notable event (Positive PCR in CSF at 2nd lumbar puncture, gastrointestinal bleed, hyperglycaemia requiring change in medical management, opportunistic infections, unexpected/severe neuropsychiatric events).

2. Serious adverse events occurring after 30 days from randomisation are monitored through reporting in the CRFs with safety data collected in the 26 week and 78-week CRFs if serious.

The research investigator at each study site (or designated other) assesses all adverse events for seriousness, causality and severity. The Chief Investigator (or designated other) assesses all adverse drug reactions for expectedness from known side effects of the use of Dexamethasone.[43] All serious ARs, AEs and SUSARs occurring up to 30 days from randomisation (apart from death unless the investigator suspects causality) require reporting to the clinical trials unit, within 24 hours of the site becoming aware of the event. In the case of death of a patient causality will be assessed by the trial steering committee.

The clinical trials unit will notify the Medicines and Healthcare products Regulatory Agency and main research ethics committee of all SUSARs that occur during the study according to the following timelines; fatal and life-threatening within 7 days of notification and non-life threatening within 15 days. All investigators are informed of all SUSARs occurring throughout the study.

SAEs occurring after 30 days from randomisation are monitored by the clinical trials unit via the 26 week and 78week CRFs. These CRFs need to be received at the clinical trials research unit by 4 weeks after the 26 and 6 weeks after 78week time points.

For peer review only - http://bmjopen.bmj.com/site/about/guidelines.xhtml

Safety data is provided to the IDSMC, who are responsible for safeguarding the interests of trial participants and assessing the safety of the interventions during the trial; the IDSMC ensures action is taken as needed should they become aware of trends in reported AEs that raise safety concerns.

Ethical Considerations

The trial falls within the remit of the EU Directive 2001/20/EC, transposed into UK law as the UK Statutory Instrument 2004 No 1031: Medicines for Human Use (Clinical Trials) Regulations 2004 as amended. This trial is registered with the MHRA and granted Clinical Trial Authorisation (CTA). The EUDRACT number for CTA reference is 2015-001609-16. Ethical approval has been obtained from a multi-centre research ethics committee familiar with the principals of the Mental Capacity Act 2005 guidance for sites in England and Wales and the Adults with Incapacity Act 2008 for sites in Scotland as the principals are relevant to a clinical trial of investigational medicinal products (CTIMP). Clinical Research Governance approval was given through the Sponsor, The University of Liverpool. The trial protocol was approved by a National Research Ethics Service Committee reference is 2015-001609-16 (Attained 31/03/2016) and underwent independent review at the Research and Development offices at participating sites. This study abides by the principles of the World Medical Association Declaration of Helsinki (1964) and Tokyo (1975), Venice (1983), Hong Kong (1989) and South Africa (1996). Due to the nature of this trial it also abides by the Medicine for Human Use (Clinical Trials) regulations 2004 (S.I.2004:1031) and all following amendments which are incorporated into UK law.

For peer review only

For peer review only - http://bmjopen.bmj.com/site/about/guidelines.xhtml

503 **Informed Consent Process**

504

505 In obtaining and documenting informed consent, the investigators adhere to National
506 Institute for Health Research (NIHR) Good Clinical Practice (GCP) guidelines and the ethical
507 principles derived from the Declaration of Helsinki. Staff delegated by the principal
508 investigator and appropriately trained with experience in obtaining informed consent, discuss
509 the objectives, risks and inconveniences of the trial and the conditions under which it is to be
510 conducted with the patient or if the patient lacks capacity with a legal or professional
511 representative. Trial information documents and points of contact for further information are
512 provided and the potential participants are given adequate time to consider their decision.

513

514 As this is a CTIMP, the clinical trial regulations for incapacitated adults are followed
515 (Medicines for Human Use Clinical Trial Regulations 2004 and amendments). When a legal
516 representative has given consent for a patient to participate in the trial and the patient
517 subsequently regains capacity, the research team will provide the patient information sheet
518 and request consent from the participant. Patients are allowed to withdraw from the study
519 at any point and may request withdrawal of their data collected until this point. Prospective
520 consent can also be obtained prior to a positive PCR result so participants may have adequate
521 time for contemplation.

522

523 As suspected encephalitis is a medical emergency, a deferred consent process is used for the
524 collection and retention of some samples as part of routine clinical management. Using
525 emergency deferred consent for samples involves taking additional samples of blood and CSF
526 only if the procedure is being performed for clinical care. If deferred consent has been used,

For peer review only

written consent is requested from either the patient or a legal representative as soon as is possible and appropriate, with samples discarded if this is declined. This approach is based on discussions with patients and the public through the Encephalitis Society.

Data Capture Methods

Data is stored securely in line with the Data Protection Act 1998. The randomisation system, data capture form and CRF have been designed to optimally protect participant information and to maintain confidentiality. Trial data is captured at local sites using paper CRFs. These are then sent into the clinical trials research unit for data entry into the study specific database. Completed CRFs are returned to clinical trial research centre within 7 days of completion. A copy of the CRF sent over to the clinical trials research unit is retained at site. CRFs and consent forms are stored separately and securely at all times in dedicated areas of the clinical trials research unit.

CRFs are checked for data quality by the clinical trials research unit in Liverpool responsible for ensuring data collection and storage.

Patients' anonymised and labelled neuroimaging data are put on to discs at site and sent to the clinical trials research unit; the images can also be transferred via the Image Exchange Portal in an encrypted manner. The final dataset will be solely accessible to the central study team at the University of Liverpool for analysis and write up.

Trial Funding and Financial Arrangements

For peer review only

For peer review only - http://bmjopen.bmj.com/site/about/guidelines.xhtml

This trial is funded by the NIHR Efficacy and Mechanism Evaluation Programme for the Department of Health. Contractual agreements are in place between the Sponsor and collaborating centres that describe financial arrangements. Trial participants are not paid to participate in the trial but are paid travel expenses for the follow up visits, estimated at £50 per visit. Payments to sites are made per site initiation but the bulk of payments are made per patient recruitment. Sites receive payment for: clinical time oversight, research nurse time, administrative support, magnetic resonance imaging scanning and pharmacy oversight.

For peer review only

Patient and public involvement

The Encephalitis Society was consulted and provided advice on the design of the trial and the difficulties participants and their families will likely encounter. The Chief Executive of The Encephalitis Society is a co-applicant on the grant application and a co-author on this paper.

The Encephalitis Society has also provided patient representatives at our trial steering committee and assisted in the production and dissemination of trial promotional materials. The Encephalitis Society will drive forward publication and dissemination of the trial findings among lay, therapeutic and health professionals through the use of web materials, newsletters and guides as well as at conferences and seminars in relation to encephalitis and related fields. All patients and their family/carers will be acknowledged in any outputs from the trial. We also work with The Encephalitis Society on a programme of teaching events and produce guides for healthcare professionals and lay people.

For peer review only - http://bmjopen.bmj.com/site/about/guidelines.xhtml

In instances where trial participants and their families have ongoing difficulties the central study team seek help for them through the Encephalitis Society and appropriate specialists for further assistance.

Discussion

This protocol describes the design of a randomised controlled trial to demonstrate the utility of using dexamethasone in the management of individuals with HSV encephalitis. HSV encephalitis is a rare sporadic acute disease, and the trial has been designed to take this challenge into account, along with the practicalities of running the trial in a UK National Health Service setting. In particular lessons were learnt from a previous similar European study, the German trial of Aciclovir and Corticosteroids in Herpes simplex virus Encephalitis (GACHE trial), which was stopped early because of recruitment difficulties. Recruitment to the GACHE trial necessitated patients had focal neurological signs of no longer than five days prior to admission, whilst DexEnceph has less stringent criteria and reflects the diverse ways in which HSV encephalitis may present. DexEnceph has been designed to be both practical and pragmatic, in that patients must be recruited within 7 days of the PCR result becoming available. This allows for occasions where it may take longer to get the PCR performed, and also allows time for patients admitted to district general hospitals, which may not be study centres, to be transferred to larger hospitals which are. DexEnceph also has the advantage in that its recruitment projections were based on preliminary data garnered from the ENCEPH-UK NIHR program (www.encephuk.org) and from a multicentre cohort study of encephalitis in England, run by the Health Protection Agency (fore-runner to the Health Protection Agency).[9] These two studies provided direct information on the number of HSV encephalitis

For peer review only - http://bmjopen.bmj.com/site/about/guidelines.xhtml

patients presenting to UK hospitals. Our choice of an open label observer-blind study, rather than a placebo-controlled double-blind trial, avoided the logistic challenges of ensuring blinded study drug was available across the large number of centres, necessitated by a rare sporadic disease, which may also have been a factor in the recruitment difficulties experienced by the GACHE trial. We are confident our robust monitoring and trial promotion ensures the majority of eligible patients are recruited. N- methyl-D-aspartic acid (NMDA) receptor antibody encephalitis (which is treated with corticosteroids and other immunomodulatory therapies) is being recognised increasingly as a late complication of HSV encephalitis. [44,45] DexEnceph may also be able examine whether corticosteroids reduce the incidence of this complication. If there is demonstrable efficacy of corticosteroid in improving neuropsychological, imaging and quality of life outcomes, without compromising patient safety the results will be far reaching.

Collaboration with France

Because we recognised from the start that there may be difficulties keeping to recruitment targets in the DexEnceph study, we worked with colleagues in France to develop a parallel French study (DexEnceph-France). This follows the UK DexEnceph protocol as closely as possible, whilst being pragmatic about the constraints of a different country's health care system. The French trial is based in 10 hospitals with the lead centre being Grenoble Alpes University Hospital and the aim of recruiting 30 patients.

The intention is for the two trials to be analysed separately, with the option of also results also combining them into an overall analysis which will give additional power to detect a treatment effect.

For peer review only

For peer review only - http://bmjopen.bmj.com/site/about/guidelines.xhtml

Trial status

The trial has been open across the UK since August 2016, and as of July 2019 is open at 45 NHS trusts; 71 patients have been randomised (of the total target of 90) meaning that we have recruited 79% of the patients are recruiting at just over 80% of the target recruitment rate. The trial was due to complete recruitment later in 2020 with 6 months follow up and 3 months post trial closure for write up. Though recent COVID-19 pandemic has placed difficulties in conducting trials and paused recruitment, the primary outcome has not been missed in any existing DexEnceph recruit, this has been achieved by conducting neuropsychology assessment by either telephone or online video discussion, thus avoiding any risk of transmission. DexEnceph-France study, which opened in 2018 has recruited 10 patients from 10 sites.

If you are interested to find out more information or to see if your trust is involved visit www.dexenceph.org.uk or for more information please email: dexenceph@liverpool.ac.uk.

Dissemination

The results of the DexEnceph trial will be published in a high impact journal in a timely manner to present the findings to front-line clinicians. Authorship of the final papers will be determined in accordance with the international committee of medical journal editors’

For peer review only

guidelines. The investigators will be involved in the preparation and drafting of the manuscripts. There is no intended use of professional writers.

Declaration of Interest

TS is supported by the National Institute for Health Research (NIHR) Health Protection Research Unit in Emerging and Zoonotic Infections (Grant No. IS-HPU-1112-10117), NIHR Global Health Research Group on Brain Infections (No. 17/63/110), and the European Union's Horizon 2020 research and innovation program ZikaPLAN (Preparedness Latin America Network), grant agreement No. 734584.

Author Statement

All authors were consulted and inputted into the article, below lists the particular role within DexEnceph

TW: Clinical research fellow, CF: Clinical research fellow, KDavies: trials manager, SD: contributer to trial design and running, MG: Clinical and laboratory biomarkers lead, CH: Neuropsychology researcher, RT:trial pharmacist, GB: Trial statistician, AR-H: trial statistician, PM: Neuropsychology lead, KDas: Neuroimaging lead, MZ: Virology, LP: Neuroimaging, SK: Neuroimaging, NR: Neuroimaging, EA: Encephalitis Society Chief Executive advisor, RK: Clinical lead brain infections UK, TS Chief Investigator

Data Statement

There are currently no current plans to publish the data

References

For peer review only

1. Davison KL, Crowcroft NS, Ramsay ME, Brown DWG, Andrews NJ. Viral encephalitis in England, 1989-1998: What did we miss? *Emerg Infect Dis*. 2003;9(2):234-240. doi:10.3201/eid0902.020218

2. Whitley RJ. Herpes simplex encephalitis: Adolescents and adults. *Antiviral Res*. 2006;71(2-3 SPEC. ISS.):141-148. doi:10.1016/j.antiviral.2006.04.002

3. Mailles A, Stahl J. Infectious Encephalitis in France in 2007: A National Prospective Study. *Clin Infect Dis*. 2009;49(12):1838-1847. doi:10.1086/648419

4. Granerod J, Cousens S, Davies NWS, Crowcroft NS, Thomas SL. New estimates of incidence of encephalitis in England. *Emerg Infect Dis*. 2013;19(9):1455-1462. doi:10.3201/eid1909.130064

5. Solomon T, Hart IJ, Beeching NJ, Solomon T, Hart IJ, Beeching NJ. Viral encephalitis : a clinician ’ s guide. 2007;(October):288-305. doi:10.1136/jnnp.2007.129098

6. Easton A. Encephalitis: raising awareness and collaborating in research. *Lancet Neurol*. 2016;15(4):353. doi:10.1016/s1474-4422(16)00059-4

7. Mcgrath N, Anderson NE, Croxson MC, Powell KF. Herpes simplex encephalitis treated with acyclovir: diagnosis and long term outcome. *J Neurol Neurosurg Psychiatry*. 1997;63:321-326. doi:10.1136/jnnp.63.3.321

8. Jouan Y, Grammatico-Guillon L, Espitalier F, Cazals X, François P, Guillon A. Long-term outcome of severe herpes simplex encephalitis: a population-based observational study. *Crit Care*. 2015;19(1):345. doi:10.1186/s13054-015-1046-y

9. Granerod J, Ambrose HE, Davies NWS, et al. Causes of encephalitis and differences in their clinical presentations in England: A multicentre, population-based prospective study. *Lancet Infect Dis*. 2010;10(12):835-844. doi:10.1016/S1473-3099(10)70222-X

For peer review only - http://bmjopen.bmj.com/site/about/guidelines.xhtml

10. Raschilas F, Wolff M, Delatour F, et al. Outcome of and Prognostic Factors for Herpes Simplex Encephalitis in Adult Patients: Results of a Multicenter Study. *Clin Infect Dis*. 2002;35(3):254-260. doi:10.1086/341405

11. Egdell R, Egdell D, Solomon T. Herpes simplex virus encephalitis. *Bmj*. 2012;344(jun13 2):e3630-e3630. doi:10.1136/bmj.e3630

12. Utley TF, Ogden JA, Gibb A, McGrath N, Anderson NE. The long-term neuropsychological outcome of herpes simplex encephalitis in a series of unselected survivors. *Neuropsychiatry Neuropsychol Behav Neurol*. 1997;10(3):180-189. http://www.ncbi.nlm.nih.gov/pubmed/9297711.

13. Frisch S, Thiel F, Marschhauser A, Villringer A, Horstmann A, Schroeter ML. Identifying neural correlates of memory and language disturbances in herpes simplex encephalitis: a voxel-based morphometry (VBM) study. *J Neurol*. 2015;262(3):563-569. doi:10.1007/s00415-014-7604-4

14. Pewter SM, Williams WH, Haslam C, Kay JM. Neuropsychological and psychiatric profiles in acute encephalitis in adults. *Neuropsychol Rehabil*. 2007;17(4-5):478-505. doi:10.1080/09602010701202238

15. Hokkanen L, Poutiainen E, Valanne L, et al. Cognitive impairment after acute encephalitis: comparison of herpes simplex and other aetiologies. *Neurosurgery, and Psychiatry*. 1996;61(December 1994):478-484. doi:10.1136/jnnp.61.5.478

16. Cooper J, Kierans C, Defres S, Easton A, Kneen R, Solomon T. Care beyond the hospital ward: understanding the socio-medical trajectory of herpes simplex virus encephalitis. *BMC Health Serv Res*. 2017;17(1):1-11. doi:10.1186/s12913-017-2608-2

17. Cooper J, Kierans C, Defres S, Easton A, Kneen R, Solomon T. Diagnostic pathways as social and participatory practices: The case of herpes simplex encephalitis. *PLoS One*.

For peer review only

For peer review only - http://bmjopen.bmj.com/site/about/guidelines.xhtml

2016;11(3):1-15. doi:10.1371/journal.pone.0151145

18. Aurelius E, Andersson B, Forsgren M, Sköldenberg B, Strannegård. Cytokines and other markers of intrathecal immune response in patients with herpes simplex encephalitis. *J Infect Dis*. 1994;170(3):678-681. doi:10.1093/infdis/170.3.678

19. Sköldenberg B, Aurelius E, Hjalmarsson A, et al. Incidence and pathogenesis of clinical relapse after herpes simplex encephalitis in adults. *J Neurol*. 2006;253(2):163-170. doi:10.1007/s00415-005-0941-6

20. Michael BD, Griffiths MJ, Granerod J, et al. Characteristic Cytokine and Chemokine Profiles in Encephalitis of Infectious, Immune-Mediated, and Unknown Aetiology. *PLoS One*. 2016;11(1):e0146288. doi:10.1371/journal.pone.0146288

21. Wnęk M, Ressel L, Ricci E, et al. Herpes simplex encephalitis is linked with selective mitochondrial damage; a post-mortem and in vitro study. *Acta Neuropathol*. 2016;132(3):433-451. doi:10.1007/s00401-016-1597-2

22. Rösler A, Pohl M, Braune HJ, Oertel WH, Gemsa D, Sprenger H. Time course of chemokines in the cerebrospinal fluid and serum during herpes simplex type 1 encephalitis. *J Neurol Sci*. 1998;157(1):82-89. doi:10.1016/S0022-510X(98)00061-6

23. Kamei S, Taira N, Ishihara M, et al. Prognostic value of cerebrospinal fluid cytokine changes in herpes simplex virus encephalitis. *Cytokine*. 2009;46(2):187-193. doi:10.1016/j.cyto.2009.01.004

24. Taira N, Kamei S, Morita a, et al. Predictors of a prolonged clinical course in adult patients with herpes simplex virus encephalitis. *Intern Med*. 2009;48(2):89-94. doi:10.2169/internalmedicine.48.1445

25. Kapur N, Barker S, Burrows EH, et al. Herpes simplex encephalitis: long term magnetic resonance imaging and neuropsychological profile. *Neurol Neurosurg Psychiatry*.

For peer review only

For peer review only - http://bmjopen.bmj.com/site/about/guidelines.xhtml

1994;5757:1334-1342. doi:10.1136/jnnp.57.11.1334

26. Meyding-Lamadé UK, Oberlinner C, Rau PR, et al. Experimental herpes simplex virus encephalitis: a combination therapy of acyclovir and glucocorticoids reduces long-term magnetic resonance imaging abnormalities. *J Neurovirol*. 2003;9(1):118-125. doi:10.1080/13550280390173373

27. Sellner J, Dvorak F, Zhou Y, et al. Acute and long-term alteration of chemokine mRNA expression after anti-viral and anti-inflammatory treatment in herpes simplex virus encephalitis. *Neurosci Lett*. 2005;374(3):197-202. doi:10.1016/j.neulet.2004.10.054

28. Meyding-lamade U, Seyfer S, Dvorak F, et al. Experimental herpes simplex virus encephalitis: inhibition of the expression of inducible nitric oxide synthase in mouse brain tissue. *Neurosci Lett*. 2002;318:21-24.

29. Upton AR., Barwick D., Foster J. DEXAMETHASONE TREATMENT IN HERPES-SIMPLEX ENCEPHALITIS. *Lancet*. 1971;297(7693):290-291. doi:10.1016/S0140-6736(71)91019-1

30. Habel AH, Brown JK. DEXAMETHASONE IN HERPES-SIMPLEX ENCEPHALITIS. *Lancet*. 1972;299(7752):695. doi:10.1016/S0140-6736(72)90505-3

31. Defres S, Keller SS, Das K, et al. A feasibility study of quantifying longitudinal brain changes in herpes simplex virus (HSV) encephalitis using magnetic resonance imaging (MRI) and stereology. *PLoS One*. 2017;12(1):1-15. doi:10.1371/journal.pone.0170215

32. de Gans J, van de Beek D. Dexamethasone in Adults with Bacterial Meningitis. *N Engl J Med*. 2002;347(20):1549-1556. doi:10.1056/NEJMoa021334

33. Kamei S. Evaluation of combination therapy using aciclovir and corticosteroid in adult patients with herpes simplex virus encephalitis. *J Neurol Neurosurg Psychiatry*. 2005;76(11):1544-1549. doi:10.1136/jnnp.2004.049676

34. Musallam B, Matoth I, Wolf DG, Engelhard D, Averbuch D. Steroids for Deteriorating

For peer review only

Herpes Simplex Virus Encephalitis. *Pediatr Neurol*. 2007;37(3):229-232. doi:10.1016/j.pediatrneurol.2007.05.007

35. Maraş Genç H, Uyur Yalçın E, Sayan M, et al. Clinical outcomes in children with herpes simplex encephalitis receiving steroid therapy. *J Clin Virol*. 2016;80:87-92. doi:10.1016/j.jcv.2016.05.002

36. Nakano A, Yamasaki R, Miyazaki S, Horiuchi N, Kunishige M, Mitsui T. Beneficial effect of steroid pulse therapy on acute viral encephalitis. *Eur Neurol*. 2003;50(4):225-229. doi:10.1159/000073864

37. Lizarraga KJ, Alexandre LC, Ramos-Estebanez C, Merenda A. Are steroids a beneficial adjunctive therapy in the immunosuppressed patient with herpes simplex virus encephalitis? *Case Rep Neurol*. 2013;5(1):52-55. doi:10.1159/000350572

38. Mesker AJ, Bon GG, de Gans J, de Kruijk JR. Case report: a pregnant woman with herpes simplex encephalitis successfully treated with dexamethasone. *Eur J Obstet Gynecol Reprod Biol*. 2011;154(2):231-232. doi:10.1016/j.ejogrb.2010.10.014

39. Martinez-Torres F, Menon S, Pritsch M, et al. Protocol for German trial of Acyclovir and corticosteroids in Herpes-simplex-virus-encephalitis (GACHE): a multicenter, multinational, randomized, double-blind, placebo-controlled German, Austrian and Dutch trial [ISRCTN45122933]. *BMC Neurol*. 2008;8:40. doi:10.1186/1471-2377-8-40

40. The Medicines for Human Use (Clinical Trials) Regulations 2004. http://www.legislation.gov.uk/uksi/2004/1031/schedule/1/made. Accessed June 19, 2019.

41. Whitley RJ, Alford CA, Hirsch MS, et al. Vidarabine versus Acyclovir Therapy in Herpes Simplex Encephalitis. *N Engl J Med*. 1986;314(3):144-149. doi:10.1056/NEJM198601163140303

For peer review only

42. Sköldenberg B, Alestig K, Burman L, et al. ACYCLOVIR VERSUS VIDARABINE IN HERPES SIMPLEX ENCEPHALITIS. *Lancet*. 1984;324(8405):707-711. doi:10.1016/S0140-6736(84)92623-0

43. Spc. Dexamethasone SPC. 2014;(June):1-4.

44. Gilbert GJ, Leypoldt F, Dalmau J. Herpes simplex virus-1 encephalitis can trigger anti-NMDA receptor encephalitis: Case report. *Neurology*. 2014;82(22):2041-2041. doi:10.1212/01.wnl.0000450946.75616.32

45. Leypoldt F, Titulaer MJ, Aguilar E, et al. Herpes simplex virus-1 encephalitis can trigger anti-NMDA receptor encephalitis: Case report. *Neurology*. 2013;81(18):1637-1639. doi:10.1212/WNL.0b013e3182a9f531

Table 1: Time scale for patients Randomised in the DexEnceph study

	Follow-up Schedule						
Procedures	Screening	Baseline	2 weeks	Discharge or Day 30 of admission (whichever is sooner)	26 weeks	78 weeks	Premature Discontinuation
Signed Consent Form	X†	X*					
Assessment of Eligibility Criteria	X	X*					
Review of Medical History		X*					
Review of Concomitant Medications		X*	X	X	X	X	X
Physical Exam		X		X			
Study Intervention		X					
Clinical Data Collection		X		X	X	X	

For peer review only - http://bmjopen.bmj.com/site/about/guidelines.xhtml

	Follow-up Schedule						
Procedures	Screening	Baseline	2 weeks	Discharge or Day 30 of admission (whichever is sooner)	26 weeks	78 weeks	Premature Discontinuation
Magnetic resonance imaging (MRI) scan		Xµ	X		X	X	
Research Blood Testing		X	X		X		
Lumbar Puncture		Xγ	X				
Disability & Functional Outcomes				X	X	X	
Glasgow Coma Scale		X∞*	X∞	X∞	X	X	
Addenbrooke's Cognitive Examination revised				X	X	X	
Neuropsychology assessment					X	X	
Health Status and quality of life questionnaires					X	X	
Clinical Laboratory: Haematology, Biochemistry		Xα					
Assessment of Adverse Events			(X)	(X)	(X)	(X)	(X)

† Only applicable when patients are prospectively consented for the randomised controlled trial
*Procedures required before randomisation.

µ Baseline MRI done for clinical purposes can be done from hospital admission up to 7 days after randomisation
γ Diagnostic lumbar puncture for clinical purposes done prior to randomisation
α Recording of clinic al laboratory tests done for clinical purposes, NOT as part of trial
∞ Recorded prior to randomisation, daily for the first 14 days and then weekly until Discharge/30 days (whichever sooner)
(X) – As indicated/appropriate

Author Statement

For peer review only - http://bmjopen.bmj.com/site/about/guidelines.xhtml

Figure 1: Schematic Design of Randomised controlled trial

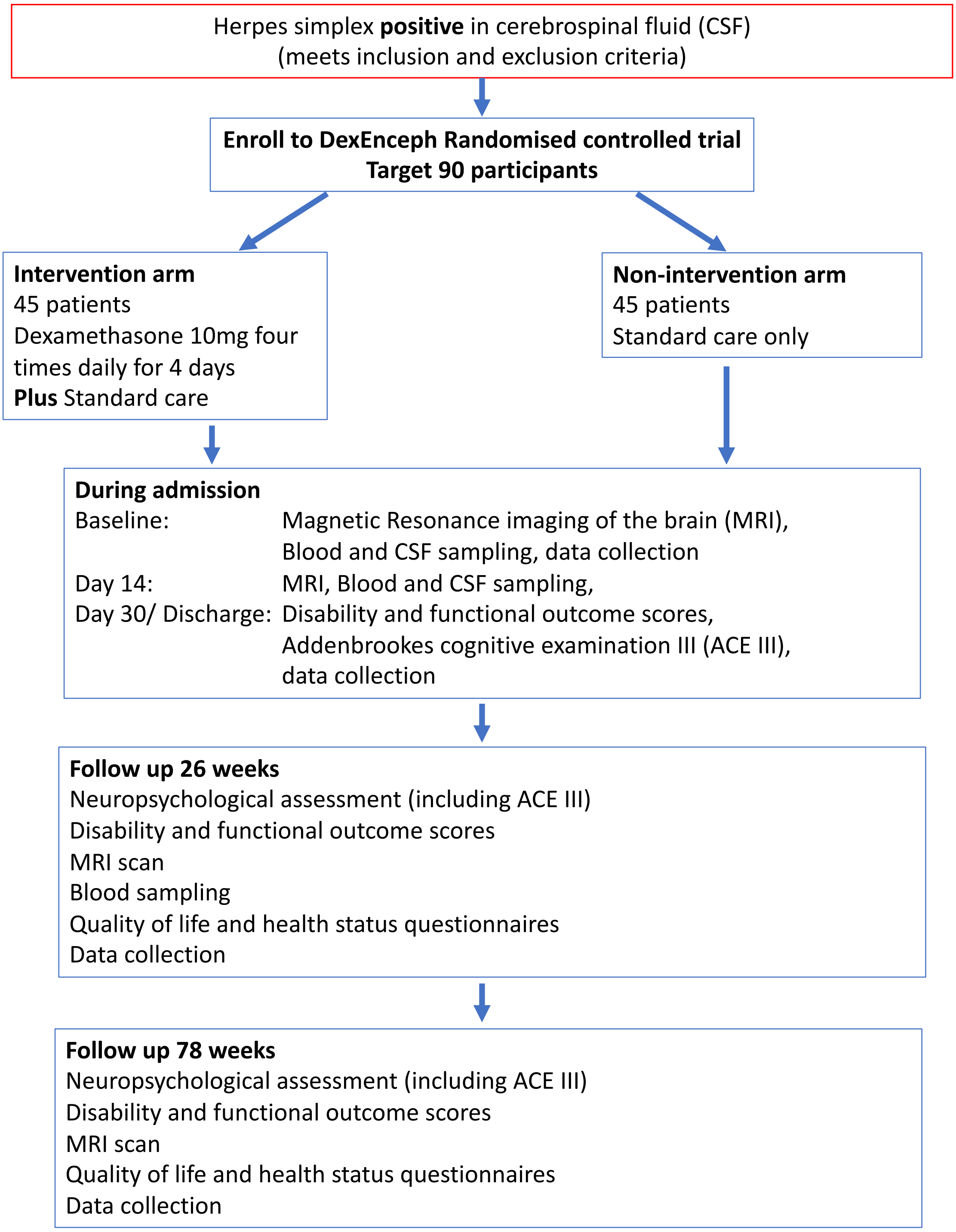

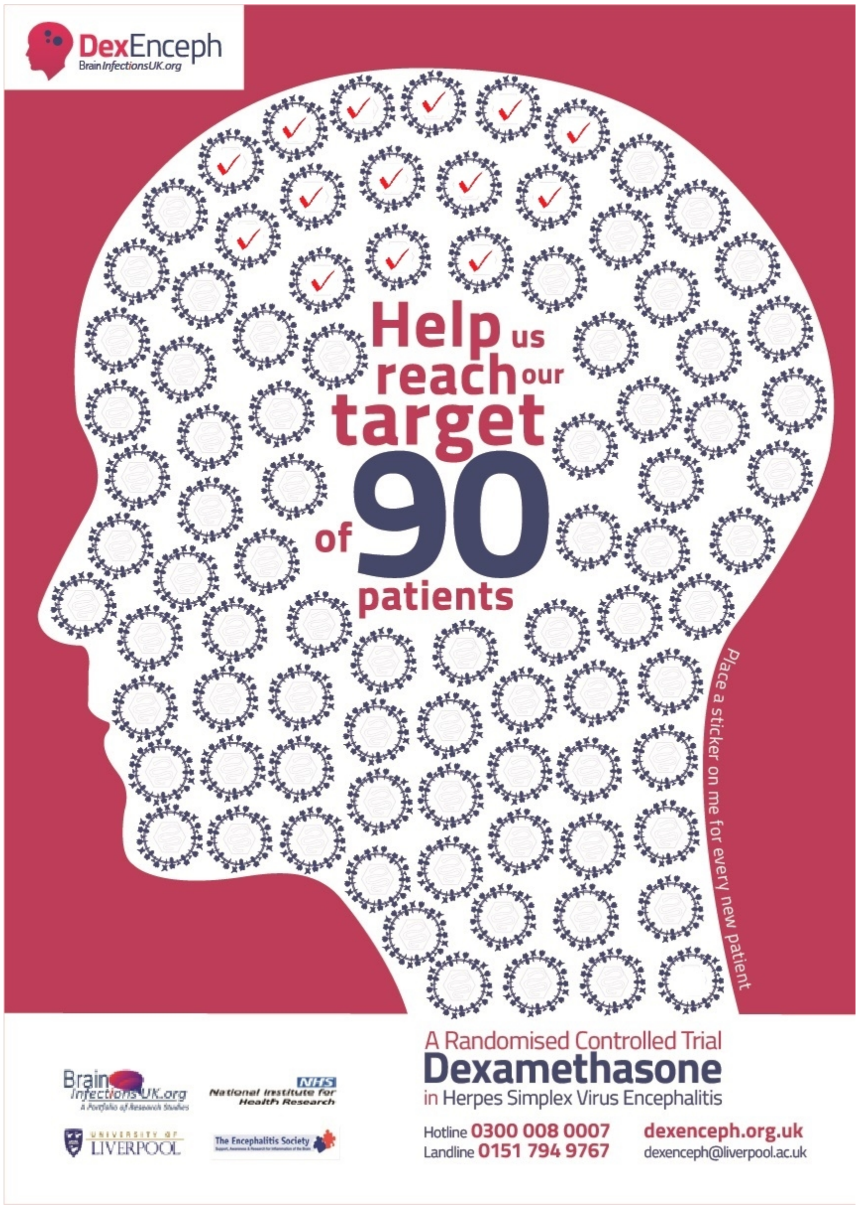

Figure 2: DexEnceph Trial recruitment poster

303x426mm (72 x 72 DPI)

University of Liverpool | NHS National Institute for Health Research

DexEnceph
Brain InfectionsUK.org

RCT Adult with Capacity Information Sheet
Version 5.0, 25 May 2018

DexEnceph: A study of dexamethasone in adults with Herpes Simplex Virus (HSV) encephalitis
Brain Infections Group, University of Liverpool

We understand this is a difficult and stressful time for you, so we firstly want to thank you for taking the time to read this leaflet.

You are being invited to take part in a research study on HSV encephalitis. This condition is extremely rare and is probably something you had never heard about before. This is why a team member will go through this leaflet with you, explaining what taking part in the study would involve and answering any questions.

Important things you need to know

- This is a study for patients with encephalitis (swelling of the brain) caused by a virus called herpes simplex virus (HSV).
- Encephalitis can make you confused, drowsy, behave out of character, affect your sleep and memory, change your mood or may cause you to have fits.
- We want to find out if reducing the swelling with a drug called dexamethasone is of benefit to patient's memory in the longer term.
- In the study there will be two groups of patients, one that receives dexamethasone and one that does not.
- If you are in the group that receives dexamethasone this will be for 4 days in hospital.
- Both groups will have the same investigations to see if dexamethasone has been of benefit.
- Dexamethasone is a commonly used drug in brain swelling and many other conditions. Like all medicines, dexamethasone has side-effects. We will explain what these can be later.

We would like to invite you to take part in a research study

- Before you decide to take part it is important you know why the research is being done and what it will involve.
- You can discuss with family, friends and clinical staff before making a decision.
- You are free to decide whether you would like to take part.
- If you choose to take part and then decide you no longer want to be involved you can stop taking part without giving a reason. Your care will not be affected.
- Please let us know if there is anything in this leaflet that is not clear or if you would like more information. A member of our team will answer your questions.
- If you decide to take part we will offer you a copy of this form and ask you to sign a consent form.

For peer review only - http://bmjopen.bmj.com/site/about/guidelines.xhtml

UNIVERSITY OF LIVERPOOL

NHS National Institute for Health Research

HSV encephalitis

1. What is HSV encephalitis?

Encephalitis means swelling of the brain and has many different causes. It is often caused by a virus. Herpes Simplex Virus (HSV) is the most common virus that causes encephalitis in the UK.

HSV encephalitis is very rare. It is diagnosed by finding the virus in fluid around the brain and spinal cord. This fluid is called CSF (cerebrospinal fluid). The CSF is obtained by the doctor who performs a lumbar puncture (LP).

HSV encephalitis is treated with the drug aciclovir. Despite treatment, some people are left with significant loss of memory. About 2 out of every 3 people will have memory difficulties long term.

The study

2. Why are we doing this study?

We know dexamethasone can reduce swelling. Reduction in swelling of the brain may improve the recovery of patients with HSV encephalitis.

This study, called DexEnceph, will allow us to compare the recovery of patients that received dexamethasone and those that did not.

3. Why have I been invited to take part?

There are two reasons why you may have been invited to take part:

A. Your doctors have diagnosed you with having HSV encephalitis.

OR

B. You may have been invited to take part before the diagnosis is made. This is because your doctors think there is a chance you may have HSV encephalitis. This will mean you have more time to think about taking part.

4. What will happen to me during the study?

All patients in the study will receive aciclovir. This is standard treatment for HSV encephalitis.

In addition, if you decide to take part in the study, you may be offered a short course of dexamethasone. This will be decided at random by a computer. This is to be fair, so neither you, your doctor, nor the research team, can choose whether you receive dexamethasone or not. Half of the people in the study will receive dexamethasone and half will not.

If you receive dexamethasone this will be 4 times a day for 4 days. It is given in a line you already have for clinical care.

What taking part involves

5. What tests are done if I take part?

When you sign up to the study:
Blood sample
CSF sample
MRI scan

↓

After 2 weeks:
MRI scan
Blood sample
CSF sample

↓

After 1 month (or when you are discharged from hospital):
Review of activities daily living
Short memory test only

↓

After 6 months:
Memory test
MRI scan
Blood sample
Review of activities daily living
Health questionnaires

↓

After 18 months:
Memory test
MRI scan
Review of activities daily living
Health questionnaires

All the tests done when you sign up to the study and the CSF tests after 2 weeks will

For peer review only - http://bmjopen.bmj.com/site/about/guidelines.xhtml

UNIVERSITY OF LIVERPOOL NHS National Institute for Health Research

be done as part of your care whether you take part in the study or not.

6. What do the memory tests involve?

These tests are the most important in the study as they will help us find out if dexamethasone improves memory problems from HSV encephalitis. These tests are sometimes called Neuropsychology tests. They are completed 6 and 18 months after the illness.

The key part of this test takes about 35 minutes. If you are not too tired we can continue with further tests that will provide useful information. These can take up to 2 hours.

They are not pass or fail tests. They provide information about your memory and thinking processes.

They can be done in one day or divided over a few short visits. If you have left hospital we can travel to see you in a convenient place for you. The test will be arranged on a day(s) which suits you.

The results can be added to your hospital notes for future reference if you wish or kept confidential within the trial.

7. What does the MRI scan involve?

As part of your care your doctor will organise an MRI scan when you are in hospital. If you take part in the study we will ask you to have another 3 scans later on.

MRI scans allow us to assess if the brain has been affected by the infection and, if so, which parts.

Each scan takes about 20 minutes. The scan can be noisy but you will be offered headphones.

The extra 3 scans are planned for:

- 2 weeks after the first one (when you are still in hospital)
- After 6 months
- After 18 months

The scans will be done at a hospital near you. We will reimburse mileage or public transport costs for any research visits.

We will check with you that you are still happy to have the scan each time. Sometimes scans may find something not related to this illness. If this happens the doctors looking at the scans will tell your own hospital doctors who will look into this further.

None of the research scans are compulsory so if you do not wish to have them you can still be part of the study.

8. Are there risks to having an MRI scan?

There are no known risks from an MRI scan. They do not use radiation. MRI scans are done routinely in patients with HSV encephalitis.

Because MRI scans use strong magnets you will not have the scan if you have any metal implants or fragments in your body.

Where you lie is quite enclosed and some people may find this unsettling. If you have a fear of confined spaces you should discuss this with your doctor before you go for the scan.

If you think you may be pregnant let your local research team know. We will not ask pregnant women to have MRI scans due to possible risks to the foetus.

9. What samples are collected? What does this involve?

We will collect blood and CSF samples during the study.

All patients with HSV encephalitis need a lumbar puncture (LP) when they come to hospital to find out why they are unwell. The doctor uses a small needle to take a sample from the lower part of the back. This is repeated after 2 weeks of treatment to see if all the virus has gone. Both lumbar punctures are part of the standard care in all patients with this condition.

For peer review only - http://bmjopen.bmj.com/site/about/guidelines.xhtml

UNIVERSITY OF LIVERPOOL
NHS National Institute for Health Research

We will take a little extra fluid at this time for the research tests. The amount of fluid we ask for each time is about 1 teaspoon, 5.5mls.

If you have already had a lumbar puncture before being told about the study, we will take stored CSF that is leftover for research tests.

Blood tests are requested at 3 different times spread over 6 months. We take between 1 to 4 teaspoons of blood, this is 5 to 23mls.

With these blood and CSF tests we will be able to better understand how the infection affects your body and how the body tries to defend itself against it.

10. What will happen to the samples that are collected? Will any genetic tests be done?

All samples will be taken at your hospital and then transported to the University of Liverpool or other laboratories supporting the study. The samples will not have any of your personal information written on them. In the University they will be stored in a secure building.

There is an option for the blood and CSF collected to have tests looking at DNA. DNA is found in all cells of the body and contains the genetic information for the working of all human beings. This study collects DNA samples to find out why some people get HSV encephalitis and others do not, and why some people have severe problems due to HSV and others do not. The information we learn from DNA may benefit others with this condition in the future but will not influence your treatment or your future health.

Some of your samples may be left over. We will ask you if they can be used for this and future studies run by the University of Liverpool.

11. How do you review activities of daily living?

We will find out how the illness has affected your day-to-day life.

The research team will look at your hospital notes. They may also talk to you and, if you choose, your relatives. This will happen when you are in hospital and when you have gone home.

We will compare patients who received dexamethasone to those that did not and see if it made a difference.

12. What are the health questionnaires?

Two questionnaires will be sent through the post. They will ask you your views about your health and quality of life. Please send them back in a pre-paid envelope.

Dexamethasone

13. What are the side effects of dexamethasone?

Dexamethasone is used widely in patients and the side-effects are well known as this medicine has been prescribed for a long time. A short course of dexamethasone will be prescribed in this study. Side effects are less common when dexamethasone is given for shorter periods.

It is important you know about the possible side-effects before you decide to take part. These are:

- Stomach pain, indigestion, having more appetite than usual, feeling or being sick.
- Feeling tired or fatigued
- Mood and behaviour changes, especially at the beginning.
- Higher blood sugars.

Other possible risks can include:

- Stomach ulcers and bleeding of ulcers.
- Decreased response to infections.

For peer review only - http://bmjopen.bmj.com/site/about/guidelines.xhtml

You will be in hospital when you take dexamethasone so you can tell your doctors immediately if you have any problem.

If you suffer side effects you or your doctor can decide to stop the dexamethasone at any point.

Dexamethasone is prescribed to women who are pregnant or breast feeding as there are no known risks to the foetus.

Other things to consider

14. Do I have to take part?

No, taking part is voluntary. If you agree, we will ask you to sign a consent form.

If you agree to take part you are free to change your mind at any time, without giving a reason. You may decide to have only some tests in the study without having to drop out of the study altogether. This will not affect the standard of care you receive.

If you withdraw from the study we will stop collecting data. We will ask you if we can use the information and samples we have gathered up to the point that you withdraw.

15. What happens if there is a problem?

If you have any concerns about any part of this study, please speak with your hospital doctor (consultant) or one of your research team.

If you remain unhappy and wish to complain formally you can do this using the NHS Complaints Procedure. You can get information on how to do this from the Patients Advice Liaison department (PALS) in your hospital.

If you suffer harm from taking part in this study, there are no special compensation arrangements. If harm occurs to you and it is due to someone’s negligence, you may have grounds for legal action for compensation against the NHS hospital where you are being treated but you may have to pay your legal costs.

16. Who will know I have taken part in this study?

Only people in your clinical care team and people involved in the study will have access to personal data. With your consent we will tell your GP that you are taking part.

All information collected about you during this study will be confidential and anonymised. It will be handled, stored and destroyed in accordance with the General Data Protection Regulation.

University of Liverpool is the sponsor for this study based in the United Kingdom. We will be using information from you and your medical records in order to undertake this study and will act as the data controller for this study. This means that we are responsible for looking after your information and using it properly. University of Liverpool will keep identifiable information about you for 15 years after the study has finished

Your rights to access, change or move your information are limited, as we need to manage your information in specific ways in order for the research to be reliable and accurate. If you withdraw from the study, we will keep the information about you that we have already obtained. To safeguard your rights, we will use the minimum personally-identifiable information possible.

You can find out more about how we use your information http://www.dexenceph.org.uk/. Our Data Protection Officer is Victoria Heath and you can contact them at V.Heath@liverpool.ac.uk.

Benefits and risks

17. What are the benefits of taking part?

You may benefit from receiving dexamethasone, however we will not know this until the end of the study. You

For peer review only - http://bmjopen.bmj.com/site/about/guidelines.xhtml

may also benefit from the increased monitoring of having extra scans and memory tests.

The information we get from this study may benefit patients in the future.

18. What are the possible disadvantages and risks of taking part?

The disadvantage in taking part in this study may be the risk of having the side-effects of dexamethasone listed in question 13 (this will not be the case if you are in the group that does not have dexamethasone).

There is the inconvenience of having the dexamethasone through the drip when you are in hospital. Once you leave hospital there is the inconvenience of travelling to hospital for 2 scans, having the memory tests and completing questionnaires.

Contact details

If you have any questions about this study, then please contact the study team members:

Principal Investigator (Doctor leading this study in your hospital):

Name: ______________________________

____

Telephone:__________________________

Research Nurse:

Name: ______________________________

____

Telephone:__________________________

Name of your Hospital:________________

____

Further information

This study is being run at your hospital and many other NHS hospitals throughout the UK. It aims to recruit 90 patients over 4 years.

It is organised by the University of Liverpool and is funded by the National Institute for Health Research (NIHR), the public body in charge of research in the UK.

Our study team includes The Encephalitis Society, a charity that supports patients and families (www.encephalitis.info).

The study has been reviewed for scientific content by expert members of NIHR. The National Research Ethics Service Committee Liverpool Central has reviewed the study and given approval for it to take place.

For peer review only

For peer review only - http://bmjopen.bmj.com/site/about/guidelines.xhtml

RCT Adult with Capacity Consent Form

Version 5.0, Dated: 25/May/2018

EudraCT Number: 2015-001609-16

Centre Name: Centre Code:

Name of Principal Investigator:

Study Number:

Please complete this form. When completed give one copy to the participant to keep, send one copy to CTU [fax/encrypted email/post], and keep one in the participant's medical notes. Please put the original in the site file.

**For patient: once you have understood each statement please initial the YES OR NO box**	**YES**	**NO**
1. I confirm I have read and understand the Information Leaflet (dated DD/MMM/YYYY) for the above study, and have had the opportunity to ask questions and have these answered satisfactorily.	INITIAL IF YES	INITIAL IF NO
2. I agree to take part in this study.	INITIAL IF YES	INITIAL IF NO
3. I understand that my participation is voluntary and that I am free to withdraw from the study at any time without giving a reason and without my care or legal rights being affected.	INITIAL IF YES	INITIAL IF NO
4. I agree for my consent form and contact details to be passed to the University of Liverpool for the administration of the study.	INITIAL IF YES	INITIAL IF NO
5. I understand that relevant sections of my medical notes and any data collected during the study may be looked at by authorised individuals from the research and clinical team and Regulatory Authorities where it is relevant to my taking part in this research. I give permission for these individuals to have access to my records.	INITIAL IF YES	INITIAL IF NO
6. I agree for genetic tests to be done on blood and CSF collected. I understand these genetic tests will not be of any individual significance to me.	INITIAL IF YES	INITIAL IF NO

For peer review only - http://bmjopen.bmj.com/site/about/guidelines.xhtml

7. I agree to have MRI scans as part of the trial.	INITIAL IF YES	INITIAL IF NO
8. I agree for my GP and hospital doctors to be informed if the scan picks up something unexpected.	INITIAL IF YES	INITIAL IF NO
9. I agree to gift the remainder of any blood or CSF sample to the University of Liverpool where it will be stored for use in future research. This may include genetic tests.	INITIAL IF YES	INITIAL IF NO
10. I agree to any images or scans that are taken to be used for teaching, education and publication (in scientific journals, books or internet).	INITIAL IF YES	INITIAL IF NO
11. I agree for my GP to be informed I am taking part in this study.	INITIAL IF YES	INITIAL IF NO

Name of Participant (Please print)	Signature	Date (DD/MM/YYYY)

Researcher*	Signature	Date (DD/MM/YYYY)

*** Important:** Prior to signing please ensure local research contact details are complete on page 6.

For peer review only

Information to Research Team:

Once a Consent Form has been signed, please copy three times: One for the participant, one to file in the medical notes and fax/post/encrypted email one to CTU. Please place original in the site file.

Please fax/encrypt email/post this consent form to CTU **separately** to other anonymised trial documents (e.g. CRF).

For peer review only - http://bmjopen.bmj.com/site/about/guidelines.xhtml

BMJ Open

Protocol for DexEnceph; a randomised controlled trial of dexamethasone therapy in adults with Herpes Simplex Virus Encephalitis

Journal:	*BMJ Open*
Manuscript ID	bmjopen-2020-041808.R1
Article Type:	Protocol
Date Submitted by the Author:	01-Nov-2020
Complete List of Authors:	Whitfield, Thomas; University of Liverpool, Brain Infections Unit<br>Fernandez, Cristina; University of Liverpool, Brain Infections Unit<br>Davies, Kelly; University of Liverpool, Clinical Trials Research Centre<br>Defres, Sylviane; Institute of Infection and Global Health, Clinical infection, medical mircobiology and Immunology; Royal Liverpool and Broadgreen University Hospitals Trust, Tropical infectious diseases Unit<br>Griffiths, Michael; University of Liverpool, Brain Infections Unit; Alder Hey Children's NHS Foundation Trust, Neurology<br>Hooper, Cory; University of Liverpool, Brain Infections Unit<br>Tangney, Rebecca; Aintree University Hospitals NHS Foundation Trust<br>Burnside, Girvan; University of Liverpool Faculty of Health and Life Sciences, Biostatistics<br>Rosala- Hallas, Anna; University of Liverpool Faculty of Health and Life Sciences, Biostatistics<br>Moore, Perry; The Walton Centre NHS Foundation Trust, Dept of Clinical Neuropsychology<br>Das, Kumar; The Walton Centre NHS Foundation Trust<br>Zuckerman, Mark; King's College Hospital NHS Foundation Trust, South London Specialist Virology Centre<br>Parkes, Laura; University of Manchester Institute of Science and Technology Department of Biomolecular Science,<br>Keller, Simon; Aintree University Hospitals NHS Foundation Trust<br>Roberts, Neil; The University of Edinburgh The Queen's Medical Research Institute<br>Easton, Ava; The Encephalitis Society<br>Touati, Saber; CHU Grenoble Alpes, Service des Maladies Infectieuses et Tropicales<br>Kneen, Rachel; Alder Hey Children's NHS Foundation Trust, Department of Neurology,; Institute of Infection and Global Health, Clinical Infection, medical microbiology and Immunology<br>Stahl, JP; Infectious Diseases Department, University of Grenoble<br>Solomon, Tom; University of Liverpool Institute of Infection and Global Health; National Institute for Health Research Health Protection Research Unit in Emerging and Zoonotic Infections, University of Liverpool
**Primary Subject Heading**:	Neurology
Secondary Subject Heading:	Infectious diseases, Pharmacology and therapeutics, Intensive care

Keywords:	Neurology < INTERNAL MEDICINE, VIROLOGY, NEUROLOGY, Neurological injury < NEUROLOGY

SCHOLARONE™ Manuscripts

I, the Submitting Author has the right to grant and does grant on behalf of all authors of the Work (as defined in the below author licence), an exclusive licence and/or a non-exclusive licence for contributions from authors who are: i) UK Crown employees; ii) where BMJ has agreed a CC-BY licence shall apply, and/or iii) in accordance with the terms applicable for US Federal Government officers or employees acting as part of their official duties; on a worldwide, perpetual, irrevocable, royalty-free basis to BMJ Publishing Group Ltd ("BMJ") its licensees and where the relevant Journal is co-owned by BMJ to the co-owners of the Journal, to publish the Work in this journal and any other BMJ products and to exploit all rights, as set out in our licence.

The Submitting Author accepts and understands that any supply made under these terms is made by BMJ to the Submitting Author unless you are acting as an employee on behalf of your employer or a postgraduate student of an affiliated institution which is paying any applicable article publishing charge ("APC") for Open Access articles. Where the Submitting Author wishes to make the Work available on an Open Access basis (and intends to pay the relevant APC), the terms of reuse of such Open Access shall be governed by a Creative Commons licence – details of these licences and which Creative Commons licence will apply to this Work are set out in our licence referred to above.

Other than as permitted in any relevant BMJ Author's Self Archiving Policies, I confirm this Work has not been accepted for publication elsewhere, is not being considered for publication elsewhere and does not duplicate material already published. I confirm all authors consent to publication of this Work and authorise the granting of this licence.

For peer review only

Protocol for DexEnceph; a randomised controlled trial of dexamethasone therapy in adults with Herpes Simplex Virus Encephalitis

The study is funded by the National Institute for Health Research's Efficacy and Mechanism Evaluation (EME) programme.

The trial sponsor is the University of Liverpool, who are responsible for all aspects of study design, implementation, analysis and write up (sponsor@liverpool.ac.uk).

For peer review only

Abstract

Introduction

Herpes simplex virus (HSV) encephalitis is a severe form of brain inflammation that commonly leaves survivors and their families with devastating long-term consequences. The virus particularly targets the temporal lobe of the brain causing debilitating memory problems, especially in verbal memory. It is postulated that immunomodulation with steroids could improve outcomes by reducing brain swelling. However, there are concerns (so far not observed) that such immunosuppression might facilitate increased viral replication with resultant worsening of disease. A previous trail closed early because of slow recruitment.

Method

DexEnceph is a pragmatic multicentre, randomised, controlled, open label, observer-blind trial to determine whether adults who receive dexamethasone alongside standard treatment with aciclovir for HSV encephalitis have improved clinical outcomes compared with those who receive standard treatment alone. Overall, 90 patients with HSV encephalitis will be recruited from a target of 90 recruiting sites; patients will be randomised 1:1 to the dexamethasone or control arms of the study. The primary outcome measured is verbal memory as assessed by the Weschler Memory Scale fourth edition Auditory Memory Index at 26 weeks post randomisation. Secondary outcomes are measured up to 72 weeks include additional neuropsychological, clinical and functional outcomes as well as comparison of neuroimaging findings. Patient safety monitoring occurs throughout and includes the detection of HSV DNA in cerebrospinal fluid 2 weeks after randomisation, which is indicative of ongoing viral replication.

For peer review only

For peer review only - http://bmjopen.bmj.com/site/about/guidelines.xhtml

Discussion

DexEnceph aims to be the first completed randomised controlled trial of steroid therapy in HSV encephalitis. The results will provide evidence for future practice in managing adults with the condition and has the potential to improve outcomes in a life-changing disease.

Ethics and dissemination

The trial has ethical approval from the UK National Research Ethics committee (Liverpool Central, REF: 15/NW/0545, 10/08/2015). Protocol version 2.1 July 2019 The results will be published and presented as soon as possible on completion.

Registration numbers

ISRCTN11774734, EUDRACT 2015-001609-16

Key words: Herpes simplex, Encephalitis, Dexamethasone, Steroid, Verbal memory

For peer review only - http://bmjopen.bmj.com/site/about/guidelines.xhtml

Article Summary

'Strengths and limitations of this study', and containing up to five short bullet points, no longer than one sentence each, that relate specifically to the methods.

- DexEnceph will be the first completed randomised controlled trial of steroid in herpes simplex virus encephalitis, examining the utility and safety of steroid use.

- DexEnceph's primary end point is verbal memory score recorded at 26 weeks post randomisation, this represents the most common area of the brain to be affected.

- The recruitment target is informed by the recent Enceph-UK programme grant of encephalitis in the UK; the trial is currently open and has recruited 71 patients of a target 90.

- Concentration on site engagement with innovative, bespoke methods are key to identifying and recruiting patients to ensure trial success.

- The pragmatic open label, observer blind design of the trial is ensuring successful recruitment to date.

Authors: Whitfield T,[1] Fernandez C, [1] Davies K, [2] Defres S[1, 13, 15], Griffiths M, [1, 14] Hooper C,[1] Tangney R, [3] Burnside G, [4] Rosala-Hallas A,[4] Moore P, [12] Das K, [5] Zuckerman M, [6] Parkes L, [7], Keller S, [3] Roberts N,[8] Easton A, [1,9] Touati S,[10] Kneen R, [1,14] JP Stahl[11] & Solomon T[1, 5,15]

1. Institute of Infection and Global Health, University of Liverpool, L69 7BE, United Kingdom
2. Clinical Trials Research Centre, University of Liverpool, L12 2AP, United Kingdom
3. University Hospital Aintree, L9 7AL, United Kingdom
4. Department of Biostatistics, University of Liverpool, L69 3GL, United Kingdom

For peer review only

5. The Walton Centre NHS Foundation Trust, L9 7JL, United Kingdom
6. South London Specialist Virology Centre, Kings College Hospital NHS Foundation Trust, SE5 9RS, United Kingdom
7. Division of Neuroscience and Experimental Psychology, Faculty of Biology Medicine and Health, University of Manchester, M13 9NT, United Kingdom
8. Queens Medical Research Institute, University of Edinburgh, EH16 4TJ, United Kingdom
9. Encephalitis Society, Malton, North Yorkshire, YO17 7DT, United Kingdom
10. Département de Recherche Clinique (DRC), Grenoble Cedex, France
11. Infectious Diseases Department, University and Hospital Grenoble Alpes, Grenoble Cedex, France
12. Salford Royal NHS Foundation Trust, M6 8HD, United Kingdom
13. Tropical Infectious diseases Unit, Royal Liverpool University Hospital, L7 8XP, United Kingdom
14. Alder Hey NHS Foundation Trust, Liverpool, L14 5AB, United Kingdom
15. NIHR Health Protection Research Unit on Emerging and Zoonotic Infections, Liverpool, L69 7BE, United Kingdom

Correspondence & trial sponsor: Tom Solomon tsolomon@liverpool.ac.uk

HPRU Project Team, The Ronald Ross Building, University of Liverpool, 8 West Derby Street, Liverpool, L69 7BE

Tel: 0151 795 7552

Fax: 0151 795 5527

For peer review only

Word Count : 4900 words

Introduction

Herpes simplex virus infection (HSV) is the most commonly identified viral cause of encephalitis, inflammation and swelling of the brain caused by a virus or the body's immune system, in the UK as in most western industrialised nations.[1–4] The incidence has been estimated at 1 in 250,000-500,000,[2] with evidence this may be higher.[4] Although a rare disease, HSV encephalitis has a disproportionately large impact due to its devastating long term neuro-psychological sequelae . These can have a marked impact on the quality of life of the patient and their family and high health economic and social costs.[5,6]

Since the introduction of the antiviral drug aciclovir in the 1970s the mortality of HSV encephalitis has reduced from around 70% to 5.5-12%.[7–9] However, survivors are commonly left with neurological impairment; less than 20% of patients are able to return to work and 48% are classed as moderate to severely disabled.[10] Even when obvious disabilities have not occurred, families often report personality changes – the person they take home from hospital is simply not the same as the one before the illness. [6,11]

HSV encephalitis can cause a broad range of cognitive impairments, but impaired memory, especially verbal memory, is the most common and likely relates to the viral predilection for

For peer review only

the temporal lobe of the brain. [12,13] The verbal memory deficits manifest as difficulties remembering names of objects and people, as well as listening to and recalling spoken information such as conversations. [14,15] In addition to memory problems, difficulties in processing speed, concentration, language and executive function are also common amongst survivors of HSV encephalitis, along with fatigue, poor concentration, anxiety and depression. [7,16,17]

The pathogenic mechanisms in HSV encephalitis are not fully understood. The evidence suggests that in addition to direct viral pathogenesis, inflammation of the brain in response to the virus is a key component of the disease process. [18–21] This is supported by the observation that in the cerebrospinal fluid (CSF), higher levels of proinflammatory chemokines, especially monocyte chemotactic protein (MCP)-1, interferon γ and interleukin 6 (IL-6) are associated with a worse prognosis.[22,23] Poor prognosis is also associated with the extent of inflammation seen on neuroimaging,[24] and the degree of temporal lobe swelling is correlated with the severity of verbal memory impairment.[25]

Control of the inflammation in HSV encephalitis may improve outcome, as shown in mouse models of the disease. [26–28] Before the availability of aciclovir, corticosteroids were also used routinely as a treatment in humans with HSV encephalitis,[29,30] and more recently both cerebral oedema on imaging and cerebrospinal fluid IL-6 levels were shown to be reduced in patients given corticosteroids.[23] However, because corticosteroids cause immunosuppression which in theory facilitates increased viral replication, their role is uncertain.[31]

In other brain infections, including bacterial meningitis and tuberculous meningitis the benefit of corticosteroids has been demonstrated in large clinical trials.[32] For HSV encephalitis the

For peer review only

potential benefit of using corticosteroid as an adjunct to aciclovir therapy has been suggested from small case series and retrospective comparisons, but there has been no prospective randomised study reported. [33–38] One study, the German trial of Aciclovir and Corticosteroids in Herpes simplex virus Encephalitis (the GACHE trial) was stopped early because of poor recruitment rates. [39] However, there is clearly a need for a study to assess this question. The DexEnceph Study, a randomised controlled trial of dexamethasone in HSV encephalitis, aims to achieve this.

Trial design

DexEnceph is a pragmatic, multicentre, randomised, controlled, observer-blind trial to determine whether the addition of dexamethasone to standard aciclovir treatment improves clinical outcomes (in particular verbal memory score) for adults with HSV encephalitis. Additionally, neuroimaging and biomarkers will be assessed along with detection of HSV in the cerebrospinal fluid at 2 weeks post randomisation to monitor for difference in viral replication between the two groups.

Primary objective

To determine whether a short course of intravenous dexamethasone, in addition to standard care, improves verbal memory score in adults with HSV encephalitis at 26 weeks post treatment compared to standard care alone.

Secondary objectives

For peer review only

Secondary objectives include the following:

To determine whether dexamethasone therapy has an effect on other neuropsychological, cognitive, clinical, disability and functional outcomes in HSV encephalitis.

To assess the effect of dexamethasone therapy on brain swelling assessed by neuroimaging imaging.

To determine whether dexamethasone therapy affects clearance of HSV from cerebrospinal fluid, the emergence of anti-N-Methyl-D Aspartic acid (NMDA) receptor antibody or causes any changes in transcriptomic and proteomic profiling in the CSF and blood.

A more comprehensive list of measures is detailed in the outcomes section.

Methods and analysis

DexEnceph is an observer blind, open label, prospective, randomised, controlled trial of dexamethasone at 10mg four times daily for 4 days, versus no dexamethasone, in adults with HSV encephalitis.

Research setting

The trial is being conducted in up to 45 NHS trusts, with a recruitment target of 0-2 patients per site per year. A full list of sites involved in DexEnceph can be obtained from www.dexenceph.org.uk

Eligibility criteria

Inclusion Criteria

For peer review only

For peer review only - http://bmjopen.bmj.com/site/about/guidelines.xhtml

Enrolled patients fulfil all of the following criteria:

1. Suspected encephalitis defined as: new onset seizure or new focal neurological signs or alteration in consciousness, cognition, personality, or behaviour. Personality / behaviour change includes agitation, psychosis, somnolence, insomnia, catatonia, mood lability, altered sleep pattern.

2. A positive HSV DNA polymerase chain reaction (PCR) result from CSF, reported not more than 7 days prior to randomisation.

3. Receiving intravenous aciclovir administered as 10mg/kg three times daily or at a reduced dose if clinically indicated.

4. Age ≥ 16 years.

5. Written informed consent given by the patient or their legal representative[40]

Exclusion Criteria

Patients are excluded if they have any of the following:

1. Have received oral or injectable corticosteroid therapy in the 30 days prior to the day of admission to hospital. This does not apply to topical/ inhaled corticosteroids. [Patients who have received oral or injectable corticosteroid therapy AFTER their admission to hospital will not be excluded from the study if they consent to trial participation].

For peer review only - http://bmjopen.bmj.com/site/about/guidelines.xhtml

2. History of hypersensitivity to corticosteroids.

3. Immunosuppression secondary to:

a. Known HIV infection and CD4 white cell count under 200/mm^3

b. Currently taking biologic therapy or other immunosuppressive agents [e.g. azathioprine, methotrexate, ciclosporin]

c. Previous solid organ transplant and currently on immunosuppression

d. Previous bone marrow transplant

e. Currently undergoing a course of chemotherapy or radiotherapy

f. Known primary immunodeficiency syndrome

g. Known current haematological malignancy

4. Pre-existing indwelling ventricular devices.

5. Peptic ulcer disease in the last 6 months, defined as a peptic ulcer seen at endoscopy or an upper gastrointestinal bleed causing a ≥ 2unit haemoglobin drop, in the last 6 months.

6. Antiretroviral regime containing rilpivirine as current treatment [Levels of rilpivirne are known to significantly decrease in co-administration with dexamethasone, a switch to a suitable alternative can facilitate trial entry].

Intervention

For peer review only

234 Participants are randomised in a 1:1 ratio to dexamethasone four times daily for four days alongside standard care, or standard care alone (Figure 1). Standard care includes intravenous aciclovir for a minimum of 14 days based on an ideal body weight at 10mg/kg every 8 hours, unless dose adjustment to account for renal impairment is necessary. Participating clinicians remain free to modify or discontinue the dexamethasone administration or to give alternative treatments at any stage, if this is judged to be in the best interest of the patient.

241 Participants assigned dexamethasone receive 10 mg equivalent of ordinary ward stock, prescribed by an authorised member of the local study team, given intravenously four times daily for four days (16 doses in total) starting within 24 hours of randomisation.

245 The University of Liverpool employs a clinical trials unit to be responsible for screening, and monitoring data collection, quality and completeness. As there is a low number of participants to be recruited, the trials unit are able to liaise regularly with each site following randomisation to ensure all follow up data are collected. Primary outcome is recorded by a centrally employed roving neuropsychologist, who collects the neuropsychological outcomes at 26 and 72 weeks.

For peer review only

For peer review only - http://bmjopen.bmj.com/site/about/guidelines.xhtml

Outcome measures

Primary Outcome

Verbal memory score, determined by the Wechsler Memory Scale 4th edition (WMS-IV) Auditory Memory Index, at 26 weeks after randomisation.

Secondary Outcomes

Other neuropsychological outcome measures [at 26 weeks and 78 weeks after randomisation]:

- Verbal memory score, determined by the WMS-IV, Auditory Memory Index, at 78 weeks after randomisation.
- Visual, Immediate and Delayed Memory by Indexes of the WMS-IV, processing speed and working memory subscales from the Wechsler Adult Intelligence Scale Fourth Edition.
- Higher executive function using the Trail Making Test.
- Anxiety and Depression symptom levels by the Beck Depression Inventory and Beck Anxiety Inventory.
- Subjective cognitive complaints using the Perceived Deficits Questionnaire.

Cognitive Outcome Measures [at discharge or 30 days if still in hospital, 26 weeks and 78 weeks]

For peer review only

- Addenbrooke’s Cognitive Assessment III.

Clinical Outcomes

- Incidence of epilepsy.
- Time to hospital discharge.
- Requirement of high dependency unit or Intensive care unit admission up to 30 days post randomisation.
- Time taken to be free of ventilatory support for 14 days [if any].
- Time to reach maximum recorded Glasgow coma scale score.
- Survival.

Disability & Functional Outcomes [at discharge or 30 days if still in hospital, 26 weeks and 78 weeks]:

- Modified Rankin Score, Barthel Index, Liverpool Outcome Score and Glasgow Outcome Scale Extended.

Imaging Outcomes: Change from Baseline at 2 weeks, 26 weeks and 78 weeks

- Temporal lobe volume (as % of intra-cranial volume).
- Whole brain volume (as % of intra-cranial volume).
- Volume of affected region as seen on fluid-attenuated inversion recovery (FLAIR) image (as % of intra-cranial volume).
- Volume of affected region as seen on diffusion-weighted image (as % of intra-cranial volume).

Biomarker Outcomes:

- Transcriptomic and proteomic profiling on blood at convalescence (2 weeks and 26 weeks), compared to acute baselines, and on CSF at 2 weeks compared to acute baseline.

For peer review only

- Anti-NMDA receptor antibody testing at 26 weeks.

Safety Outcomes:

- Proportion of patients with detectable HSV in CSF by PCR at 2 weeks.

Health Status and Quality of Life [at 26 and 78 weeks]:

- Measured by the EuroQoL-5 Dimension-5 Level quality of life scale (EQ-5D-5L) and 36 item short form survey (SF-36) self-completed questionnaires.

Screening

The majority of potential patients are identified by the local research team through identifying patients with a relevant clinical presentation suspicious of HSV encephalitis and/or detection of HSV in a CSF sample. A screening log is completed for all potential patients. A strong link with the local laboratory is essential as a key factor in ensuring eligible patients are not missed by the investigative team. Investigators for the local research team include neurologists, infectious disease clinicians, microbiologist and virologists.

Because this is an uncommon disease, extra measures have been taken to try and maximise recruitment. On identifying a suitable patient, sites are able to contact the trial management team for intensive support via a dedicated telephone hotline, email, or an app. Short videos which explain the trial to patients, families and to health care workers also support recruitment. Every month, the trial management group monitor the screening reports of each site for the previous 3 months, to ensure they are actively looking for patients. Lower than expected screening is followed up by the central study team making contact with the study site to review their screening methodology and offer support.

For peer review only

Randomisation

Participants are randomised using a 24-hour secure web-based programme, which is centrally controlled by the clinical trials research centre. Designated members of the trial team at the site (detailed on the delegation of responsibilities log) are provided with a unique username and password which is required to access the web-based randomisation system. In the event of system failure, the patient can be randomised centrally electronically or through secure envelopes. Each participant is allocated a unique study number (randomisation number), the primary identifier for all the participants in this study.

The neuropsychologist collecting the primary outcome and other outcome assessors such as radiologists and those responsible for paper authorship are blinded to randomisation during the trial. Trial participants and local site study teams, as well as the trial manager and trial data manager at the clinical trials unit are aware of what treatments have been allocated. The independent data safety and monitoring committee (IDSMC) and statisticians have access to unblinded data grouped by intervention throughout the trial and make recommendations to the trial's steering committee who would only become unblinded in the event of a serious event.

Participant timeline

The time schedule for enrolment, interventions and assessments is given in table 1.

For peer review only - http://bmjopen.bmj.com/site/about/guidelines.xhtml

Statistical Considerations

Sample Size

The primary outcome variable is verbal memory , assessed as part of WMS-IV. In one published series of adults who survived HSV encephalitis, 19 of 22 had memory impairment evident at follow up, with verbal memory being most severely affected [12]. In that study the mean (standard deviation, SD) verbal memory score was 88.9 (18.9) compared with the population mean (SD) of 100 [15]. This score can only be assessed in survivors, we estimate approximately 10% of patients in the trial will die before assessment of the primary outcome[8,41,42]. In instances where the death is judged to be associated with encephalitis the verbal memory score is recorded as 40, (the lowest possible value which would be obtained even where a patient recorded no recall of any of the items administered in the memory subtests). Where the cause of death is thought to be independent of having encephalitis, those patients will be recorded as lost to follow up. Similarly, for patients who are too unwell, due to encephalitis, to undergo the assessment, the score is recorded as 40. Decisions as to whether the reasons for death or non-completion of the measures were due to encephalitis will be made by an independent committee blinded to dexamethasone allocation. Adjusting the estimate of mean and standard deviation from survivors, to include the 10% of patients with the lowest possible value of 40, gives a total population mean of 84.8, with a standard deviation of 23.1. A final sample size of 36 participants per group allows us to detect a clinically meaningful difference of 15.5 on the verbal memory score with 80% power, at a two-sided significance level of 0.05. Allowing for up to 20% dropout gives an initial target sample size of 45 participants per group, for a total of 90.

For peer review only - http://bmjopen.bmj.com/site/about/guidelines.xhtml

Statistical analysis

For the primary outcome, participants are included in the analysis based on the intention-to-treat principle. Verbal memory score will be compared between groups using linear regression. The model will be adjusted for pre-specified variables which are judged to be potentially related to the outcome, including age and admission Glasgow coma scale score. No interim analysis is planned, but there is regular monitoring by the IDSMC.

For peer review only

As there may be some missing primary outcome data due to death, inability to complete the assessment, or loss to follow up, a sensitivity analysis will be carried out. All randomised patients will be included in this analysis.

For continuous secondary outcome variables, comparisons between groups will be analysed as per the primary outcome. The results for residual viral presence in the CSF at 2 weeks will be reported with a 95% confidence interval for the difference in proportions between groups. Time to event outcomes will be analysed using Kaplan-Meier curves, log rank tests and Cox Proportional Hazards models. Binary secondary outcomes will be analysed using logistic regression.

Trial Promotion and engagement

Considerable effort is maintained to keep the principal investigators, research nurses and the community engaged in the trial. The trial is being publicised using public forums, the Encephalitis Society website and newsletter, social media, patient journey articles and work with newspapers and TV. To promote site engagement, study days are arranged for research

For peer review only - http://bmjopen.bmj.com/site/about/guidelines.xhtml

teams to attend, along with scheduled research nurse teleconferences to allow ideas on maximising recruitment and updates on trial progress to be shared. Sites are also kept updated through our website (www.dexenceph.org.uk), newsletters and an innovative sticker chart, whereby a sticker is sent out to every site each time a patient is recruited (Figure 2). The Encephalitis Society are playing a key role in providing additional support to the patients and their families aside from their work for the trial.

Trial Closure

The end of the trial is defined to be the date on which data for all participants is frozen and data entry privileges are withdrawn from the trial database. The trial may be closed prematurely by the trial steering committee, on the recommendation of the IDSMC if there is sufficient evidence of risk to patient safety.

Pharmacovigilance

Oversight of the trial is provided by the trial steering committee, which meets at least annually to review trial progress, safety, and adverse events. The committee is also informed of any protocol changes by the clinical trial research unit.

The Medicines for Human Use (Clinical Trials) Regulations 2004 (SI 2004/1031) definitions are used for Adverse Event (AE), Adverse Reaction (AR), Unexpected Adverse Reaction (UAR), Serious Adverse Event (SAE), Serious Adverse Reaction (SAR) or Suspected Unexpected Serious Adverse Reaction (SUSAR).

Depending on the nature of the event the reporting procedures below are followed:

For peer review only

For peer review only - http://bmjopen.bmj.com/site/about/guidelines.xhtml

1. Serious adverse events occurring up to 30 days after randomisation are reported through a Serious Adverse Event Form (if serious) or in the 30 day/discharge case record forms (CRF) if they are a notable event (Positive PCR in CSF at 2nd lumbar puncture, gastrointestinal bleed, hyperglycaemia requiring change in medical management, opportunistic infections, unexpected/severe neuropsychiatric events).

2. Serious adverse events occurring after 30 days from randomisation are monitored through reporting in the CRFs with safety data collected in the 26 week and 78-week CRFs if serious.

The research investigator at each study site (or designated other) assesses all adverse events for seriousness, causality and severity. The Chief Investigator (or designated other) assesses all adverse drug reactions for expectedness from known side effects of the use of Dexamethasone.[43] All serious ARs, AEs and SUSARs occurring up to 30 days from randomisation (apart from death unless the investigator suspects causality) require reporting to the clinical trials unit, within 24 hours of the site becoming aware of the event. In the case of death of a patient causality will be assessed by the trial steering committee.

The clinical trials unit will notify the Medicines and Healthcare products Regulatory Agency and main research ethics committee of all SUSARs that occur during the study according to the following timelines; fatal and life-threatening within 7 days of notification and non-life threatening within 15 days. All investigators are informed of all SUSARs occurring throughout the study.

SAEs occurring after 30 days from randomisation are monitored by the clinical trials unit via the 26 week and 78week CRFs. These CRFs need to be received at the clinical trials research unit by 4 weeks after the 26 and 6 weeks after 78week time points.

For peer review only

Safety data is provided to the IDSMC, who are responsible for safeguarding the interests of trial participants and assessing the safety of the interventions during the trial; the IDSMC ensures action is taken as needed should they become aware of trends in reported AEs that raise safety concerns.

Trial Funding and Financial Arrangements

Contractual agreements are in place between the Sponsor and collaborating centres that describe financial arrangements. Trial participants are not paid to participate in the trial but are paid travel expenses for the follow up visits, estimated at £50 per visit. Payments to sites are made per site initiation but the bulk of payments are made per patient recruitment. Sites receive payment for: clinical time oversight, research nurse time, administrative support, magnetic resonance imaging scanning and pharmacy oversight.

Patient and public involvement

The Encephalitis Society was consulted and provided advice on the design of the trial and the difficulties participants and their families will likely encounter. The Chief Executive of The Encephalitis Society is a co-applicant on the grant application and a co-author on this paper.

The Encephalitis Society has also provided patient representatives at our trial steering committee and assisted in the production and dissemination of trial promotional materials. The Encephalitis Society will drive forward publication and dissemination of the trial findings among lay, therapeutic and health professionals through the use of web materials, newsletters and guides as well as at conferences and seminars in relation to encephalitis and

For peer review only

For peer review only - http://bmjopen.bmj.com/site/about/guidelines.xhtml

related fields. All patients and their family/carers will be acknowledged in any outputs from the trial. We also work with The Encephalitis Society on a programme of teaching events and produce guides for healthcare professionals and lay people.

In instances where trial participants and their families have ongoing difficulties the central study team seek help for them through the Encephalitis Society and appropriate specialists for further assistance.

Ethics and Dissemination

The trial falls within the remit of the EU Directive 2001/20/EC, transposed into UK law as the UK Statutory Instrument 2004 No 1031: Medicines for Human Use (Clinical Trials) Regulations 2004 as amended. This trial is registered with the MHRA and granted Clinical Trial Authorisation (CTA). The EUDRACT number for CTA reference is 2015-001609-16. Ethical approval has been obtained from a multi-centre research ethics committee familiar with the principals of the Mental Capacity Act 2005 guidance for sites in England and Wales and the Adults with Incapacity Act 2008 for sites in Scotland as the principals are relevant to a clinical trial of investigational medicinal products (CTIMP). Clinical Research Governance approval was given through the Sponsor, The University of Liverpool. The trial protocol was approved by a National Research Ethics Service Committee reference is 2015-001609-16 (Attained 31/03/2016) and underwent independent review at the Research and Development offices at participating sites. This study abides by the principles of the World Medical Association Declaration of Helsinki (1964) and Tokyo (1975), Venice (1983), Hong Kong (1989) and South

For peer review only - http://bmjopen.bmj.com/site/about/guidelines.xhtml

Africa (1996). Due to the nature of this trial it also abides by the Medicine for Human Use (Clinical Trials) regulations 2004 (S.I.2004:1031) and all following amendments which are incorporated into UK law.

Informed Consent Process

In obtaining and documenting informed consent, the investigators adhere to National Institute for Health Research (NIHR) Good Clinical Practice (GCP) guidelines and the ethical principles derived from the Declaration of Helsinki. Staff delegated by the principal investigator and appropriately trained with experience in obtaining informed consent, discuss the objectives, risks and inconveniences of the trial and the conditions under which it is to be conducted with the patient or if the patient lacks capacity with a legal or professional representative. Trial information documents and points of contact for further information are provided and the potential participants are given adequate time to consider their decision(supplementary file 1).

As this is a CTIMP, the clinical trial regulations for incapacitated adults are followed (Medicines for Human Use Clinical Trial Regulations 2004 and amendments). When a legal representative has given consent for a patient to participate in the trial and the patient subsequently regains capacity, the research team will provide the patient information sheet and request consent from the participant. Patients are allowed to withdraw from the study at any point and may request withdrawal of their data collected until this point. Prospective consent can also be obtained prior to a positive PCR result so participants may have adequate time for contemplation.

For peer review only - http://bmjopen.bmj.com/site/about/guidelines.xhtml

As suspected encephalitis is a medical emergency, a deferred consent process is used for the collection and retention of some samples as part of routine clinical management. Using emergency deferred consent for samples involves taking additional samples of blood and CSF only if the procedure is being performed for clinical care. If deferred consent has been used, written consent is requested from either the patient or a legal representative as soon as is possible and appropriate, with samples discarded if this is declined. This approach is based on discussions with patients and the public through the Encephalitis Society.

Data Capture Methods

Data is stored securely in line with the Data Protection Act 1998. The randomisation system, data capture form and CRF have been designed to optimally protect participant information and to maintain confidentiality. Trial data is captured at local sites using paper CRFs. These are then sent into the clinical trials research unit for data entry into the study specific database. Completed CRFs are returned to clinical trial research centre within 7 days of completion. A copy of the CRF sent over to the clinical trials research unit is retained at site. CRFs and consent forms are stored separately and securely at all times in dedicated areas of the clinical trials research unit.

CRFs are checked for data quality by the clinical trials research unit in Liverpool responsible for ensuring data collection and storage.

Patients' anonymised and labelled neuroimaging data are put on to discs at site and sent to the clinical trials research unit; the images can also be transferred via the Image Exchange

For peer review only

Portal in an encrypted manner. The final dataset will be solely accessible to the central study team at the University of Liverpool for analysis and write up.

Dissemination

The results of the DexEnceph trial will be published in a high impact journal in a timely manner to present the findings to front-line clinicians. They will be presented at the annual conference organised by the Encephalitis Society and at other meetings. Authorship of the final papers will be determined in accordance with the international committee of medical journal editors' guidelines. The investigators will be involved in the preparation and drafting of the manuscripts. There is no intended use of professional writers.

For peer review only

Discussion

This protocol describes the design of a randomised controlled trial to demonstrate the utility of using dexamethasone in the management of individuals with HSV encephalitis. HSV encephalitis is a rare sporadic acute disease, and the trial has been designed to take this challenge into account, along with the practicalities of running the trial in a UK National Health Service setting. In particular lessons were learnt from a previous similar European study, the German trial of Aciclovir and Corticosteroids in Herpes simplex virus Encephalitis (GACHE trial), which was stopped early because of recruitment difficulties. Recruitment to the GACHE

For peer review only - http://bmjopen.bmj.com/site/about/guidelines.xhtml

trial necessitated patients had focal neurological signs of no longer than five days prior to admission, whilst DexEnceph has less stringent criteria and reflects the diverse ways in which HSV encephalitis may present. DexEnceph has been designed to be both practical and pragmatic, in that patients must be recruited within 7 days of the PCR result becoming available. This allows for occasions where it may take longer to get the PCR performed, and also allows time for patients admitted to district general hospitals, which may not be study centres, to be transferred to larger hospitals which are. DexEnceph also has the advantage in that its recruitment projections were based on preliminary data garnered from the ENCEPH-UK NIHR program (www.encephuk.org) and from a multicentre cohort study of encephalitis in England, run by the Health Protection Agency (fore-runner to the Health Protection Agency).[9] These two studies provided direct information on the number of HSV encephalitis patients presenting to UK hospitals. Our choice of an open label observer-blind study, rather than a placebo-controlled double-blind trial, avoided the logistic challenges of ensuring blinded study drug was available across the large number of centres, necessitated by a rare sporadic disease, which may also have been a factor in the recruitment difficulties experienced by the GACHE trial. We are confident our robust monitoring and trial promotion ensures the majority of eligible patients are recruited. N- methyl-D-aspartic acid (NMDA) receptor antibody encephalitis (which is treated with corticosteroids and other immunomodulatory therapies) is being recognised increasingly as a late complication of HSV encephalitis. [44,45] DexEnceph may also be able examine whether corticosteroids reduce the incidence of this complication. If there is demonstrable efficacy of corticosteroid in improving neuropsychological, imaging and quality of life outcomes, without compromising patient safety the results will be far reaching.

For peer review only - http://bmjopen.bmj.com/site/about/guidelines.xhtml

Collaboration with France

Because we recognised from the start that there may be difficulties keeping to recruitment targets in the DexEnceph study, we worked with colleagues in France to develop a parallel French study (DexEnceph-France). This follows the UK DexEnceph protocol as closely as possible, whilst being pragmatic about the constraints of a different country's health care system. The French trial is based in 10 hospitals with the lead centre being Grenoble Alpes University Hospital and the aim of recruiting 30 patients.

The intention is for the two trials to be analysed separately, with the option of also results also combining them into an overall analysis which will give additional power to detect a treatment effect.

Trial status

The trial has been open across the UK since August 2016, and as of July 2019 is open at 45 NHS trusts; 71 patients have been randomised (of the total target of 90) meaning that we have recruited 79% of the patients are recruiting at just over 80% of the target recruitment rate. The trial was due to complete recruitment later in 2020 with 6 months follow up and 3 months post trial closure for write up. Though recent COVID-19 pandemic has placed difficulties in conducting trials and paused recruitment, the primary outcome has not been missed in any existing DexEnceph recruit, this has been achieved by conducting

For peer review only - http://bmjopen.bmj.com/site/about/guidelines.xhtml

neuropsychology assessment by either telephone or online video discussion, thus avoiding any risk of transmission. DexEnceph-France study, which opened in 2018 has recruited 10 patients from 10 sites.

If you are interested to find out more information or to see if your trust is involved visit www.dexenceph.org.uk or for more information please email: dexenceph@liverpool.ac.uk.

References

1. Davison KL, Crowcroft NS, Ramsay ME, Brown DWG, Andrews NJ. Viral encephalitis in England, 1989-1998: What did we miss? *Emerg Infect Dis*. 2003;9(2):234-240. doi:10.3201/eid0902.020218

2. Whitley RJ. Herpes simplex encephalitis: Adolescents and adults. *Antiviral Res*. 2006;71(2-3 SPEC. ISS.):141-148. doi:10.1016/j.antiviral.2006.04.002

3. Mailles A, Stahl J. Infectious Encephalitis in France in 2007: A National Prospective Study. *Clin Infect Dis*. 2009;49(12):1838-1847. doi:10.1086/648419

4. Granerod J, Cousens S, Davies NWS, Crowcroft NS, Thomas SL. New estimates of incidence of encephalitis in England. *Emerg Infect Dis*. 2013;19(9):1455-1462. doi:10.3201/eid1909.130064

5. Solomon T, Hart IJ, Beeching NJ, Solomon T, Hart IJ, Beeching NJ. Viral encephalitis : a

For peer review only

clinician ' s guide. 2007;(October):288-305. doi:10.1136/jnnp.2007.129098

6. Easton A. Encephalitis: raising awareness and collaborating in research. *Lancet Neurol*. 2016;15(4):353. doi:10.1016/s1474-4422(16)00059-4

7. Mcgrath N, Anderson NE, Croxson MC, Powell KF. Herpes simplex encephalitis treated with acyclovir: diagnosis and long term outcome. *J Neurol Neurosurg Psychiatry*. 1997;63:321-326. doi:10.1136/jnnp.63.3.321

8. Jouan Y, Grammatico-Guillon L, Espitalier F, Cazals X, François P, Guillon A. Long-term outcome of severe herpes simplex encephalitis: a population-based observational study. *Crit Care*. 2015;19(1):345. doi:10.1186/s13054-015-1046-y

9. Granerod J, Ambrose HE, Davies NWS, et al. Causes of encephalitis and differences in their clinical presentations in England: A multicentre, population-based prospective study. *Lancet Infect Dis*. 2010;10(12):835-844. doi:10.1016/S1473-3099(10)70222-X

10. Raschilas F, Wolff M, Delatour F, et al. Outcome of and Prognostic Factors for Herpes Simplex Encephalitis in Adult Patients: Results of a Multicenter Study. *Clin Infect Dis*. 2002;35(3):254-260. doi:10.1086/341405

11. Egdell R, Egdell D, Solomon T. Herpes simplex virus encephalitis. *Bmj*. 2012;344(jun13 2):e3630-e3630. doi:10.1136/bmj.e3630

12. Utley TF, Ogden JA, Gibb A, McGrath N, Anderson NE. The long-term neuropsychological outcome of herpes simplex encephalitis in a series of unselected survivors. *Neuropsychiatry Neuropsychol Behav Neurol*. 1997;10(3):180-189. http://www.ncbi.nlm.nih.gov/pubmed/9297711.

13. Frisch S, Thiel F, Marschhauser A, Villringer A, Horstmann A, Schroeter ML. Identifying neural correlates of memory and language disturbances in herpes simplex encephalitis: a voxel-based morphometry (VBM) study. *J Neurol*. 2015;262(3):563-

For peer review only

569. doi:10.1007/s00415-014-7604-4

14. Pewter SM, Williams WH, Haslam C, Kay JM. Neuropsychological and psychiatric profiles in acute encephalitis in adults. *Neuropsychol Rehabil*. 2007;17(4-5):478-505. doi:10.1080/09602010701202238

15. Hokkanen L, Poutiainen E, Valanne L, et al. Cognitive impairment after acute encephalitis: comparison of herpes simplex and other aetiologies. *Neurosurgery, and Psychiatry*. 1996;61(December 1994):478-484. doi:10.1136/jnnp.61.5.478

16. Cooper J, Kierans C, Defres S, Easton A, Kneen R, Solomon T. Care beyond the hospital ward: understanding the socio-medical trajectory of herpes simplex virus encephalitis. *BMC Health Serv Res*. 2017;17(1):1-11. doi:10.1186/s12913-017-2608-2

17. Cooper J, Kierans C, Defres S, Easton A, Kneen R, Solomon T. Diagnostic pathways as social and participatory practices: The case of herpes simplex encephalitis. *PLoS One*. 2016;11(3):1-15. doi:10.1371/journal.pone.0151145

18. Aurelius E, Andersson B, Forsgren M, Sköldenberg B, Strannegård. Cytokines and other markers of intrathecal immune response in patients with herpes simplex encephalitis. *J Infect Dis*. 1994;170(3):678-681. doi:10.1093/infdis/170.3.678

19. Sköldenberg B, Aurelius E, Hjalmarsson A, et al. Incidence and pathogenesis of clinical relapse after herpes simplex encephalitis in adults. *J Neurol*. 2006;253(2):163-170. doi:10.1007/s00415-005-0941-6

20. Michael BD, Griffiths MJ, Granerod J, et al. Characteristic Cytokine and Chemokine Profiles in Encephalitis of Infectious, Immune-Mediated, and Unknown Aetiology. *PLoS One*. 2016;11(1):e0146288. doi:10.1371/journal.pone.0146288

21. Wnęk M, Ressel L, Ricci E, et al. Herpes simplex encephalitis is linked with selective mitochondrial damage; a post-mortem and in vitro study. *Acta Neuropathol*.

For peer review only

2016;132(3):433-451. doi:10.1007/s00401-016-1597-2

22. Rösler A, Pohl M, Braune HJ, Oertel WH, Gemsa D, Sprenger H. Time course of chemokines in the cerebrospinal fluid and serum during herpes simplex type 1 encephalitis. *J Neurol Sci*. 1998;157(1):82-89. doi:10.1016/S0022-510X(98)00061-6

23. Kamei S, Taira N, Ishihara M, et al. Prognostic value of cerebrospinal fluid cytokine changes in herpes simplex virus encephalitis. *Cytokine*. 2009;46(2):187-193. doi:10.1016/j.cyto.2009.01.004

24. Taira N, Kamei S, Morita a, et al. Predictors of a prolonged clinical course in adult patients with herpes simplex virus encephalitis. *Intern Med*. 2009;48(2):89-94. doi:10.2169/internalmedicine.48.1445

25. Kapur N, Barker S, Burrows EH, et al. Herpes simplex encephalitis: long term magnetic resonance imaging and neuropsychological profile. *Neurol Neurosurg Psychiatry*. 1994;5757:1334-1342. doi:10.1136/jnnp.57.11.1334

26. Meyding-Lamadé UK, Oberlinner C, Rau PR, et al. Experimental herpes simplex virus encephalitis: a combination therapy of acyclovir and glucocorticoids reduces long-term magnetic resonance imaging abnormalities. *J Neurovirol*. 2003;9(1):118-125. doi:10.1080/13550280390173373

27. Sellner J, Dvorak F, Zhou Y, et al. Acute and long-term alteration of chemokine mRNA expression after anti-viral and anti-inflammatory treatment in herpes simplex virus encephalitis. *Neurosci Lett*. 2005;374(3):197-202. doi:10.1016/j.neulet.2004.10.054

28. Meyding-lamade U, Seyfer S, Dvorak F, et al. Experimental herpes simplex virus encephalitis: inhibition of the expression of inducible nitric oxide synthase in mouse brain tissue. *Neurosci Lett*. 2002;318:21-24.

29. Upton AR., Barwick D., Foster J. DEXAMETHASONE TREATMENT IN HERPES-SIMPLEX

For peer review only

For peer review only - http://bmjopen.bmj.com/site/about/guidelines.xhtml

ENCEPHALITIS. *Lancet*. 1971;297(7693):290-291. doi:10.1016/S0140-6736(71)91019-1

30. Habel AH, Brown JK. DEXAMETHASONE IN HERPES-SIMPLEX ENCEPHALITIS. *Lancet*. 1972;299(7752):695. doi:10.1016/S0140-6736(72)90505-3

31. Defres S, Keller SS, Das K, et al. A feasibility study of quantifying longitudinal brain changes in herpes simplex virus (HSV) encephalitis using magnetic resonance imaging (MRI) and stereology. *PLoS One*. 2017;12(1):1-15. doi:10.1371/journal.pone.0170215

32. de Gans J, van de Beek D. Dexamethasone in Adults with Bacterial Meningitis. *N Engl J Med*. 2002;347(20):1549-1556. doi:10.1056/NEJMoa021334

33. Kamei S. Evaluation of combination therapy using aciclovir and corticosteroid in adult patients with herpes simplex virus encephalitis. *J Neurol Neurosurg Psychiatry*. 2005;76(11):1544-1549. doi:10.1136/jnnp.2004.049676

34. Musallam B, Matoth I, Wolf DG, Engelhard D, Averbuch D. Steroids for Deteriorating Herpes Simplex Virus Encephalitis. *Pediatr Neurol*. 2007;37(3):229-232. doi:10.1016/j.pediatrneurol.2007.05.007

35. Maraş Genç H, Uyur Yalçın E, Sayan M, et al. Clinical outcomes in children with herpes simplex encephalitis receiving steroid therapy. *J Clin Virol*. 2016;80:87-92. doi:10.1016/j.jcv.2016.05.002

36. Nakano A, Yamasaki R, Miyazaki S, Horiuchi N, Kunishige M, Mitsui T. Beneficial effect of steroid pulse therapy on acute viral encephalitis. *Eur Neurol*. 2003;50(4):225-229. doi:10.1159/000073864

37. Lizarraga KJ, Alexandre LC, Ramos-Estebanez C, Merenda A. Are steroids a beneficial adjunctive therapy in the immunosuppressed patient with herpes simplex virus encephalitis? *Case Rep Neurol*. 2013;5(1):52-55. doi:10.1159/000350572

38. Mesker AJ, Bon GG, de Gans J, de Kruijk JR. Case report: a pregnant woman with

For peer review only

For peer review only - http://bmjopen.bmj.com/site/about/guidelines.xhtml

herpes simplex encephalitis successfully treated with dexamethasone. *Eur J Obstet Gynecol Reprod Biol*. 2011;154(2):231-232. doi:10.1016/j.ejogrb.2010.10.014

39. Martinez-Torres F, Menon S, Pritsch M, et al. Protocol for German trial of Acyclovir and corticosteroids in Herpes-simplex-virus-encephalitis (GACHE): a multicenter, multinational, randomized, double-blind, placebo-controlled German, Austrian and Dutch trial [ISRCTN45122933]. *BMC Neurol*. 2008;8:40. doi:10.1186/1471-2377-8-40

40. The Medicines for Human Use (Clinical Trials) Regulations 2004. http://www.legislation.gov.uk/uksi/2004/1031/schedule/1/made. Accessed June 19, 2019.

41. Whitley RJ, Alford CA, Hirsch MS, et al. Vidarabine versus Acyclovir Therapy in Herpes Simplex Encephalitis. *N Engl J Med*. 1986;314(3):144-149. doi:10.1056/NEJM198601163140303

42. Sköldenberg B, Alestig K, Burman L, et al. ACYCLOVIR VERSUS VIDARABINE IN HERPES SIMPLEX ENCEPHALITIS. *Lancet*. 1984;324(8405):707-711. doi:10.1016/S0140-6736(84)92623-0

43. Spc. Dexamethasone SPC. 2014;(June):1-4.

44. Gilbert GJ, Leypoldt F, Dalmau J. Herpes simplex virus-1 encephalitis can trigger anti-NMDA receptor encephalitis: Case report. *Neurology*. 2014;82(22):2041-2041. doi:10.1212/01.wnl.0000450946.75616.32

45. Leypoldt F, Titulaer MJ, Aguilar E, et al. Herpes simplex virus-1 encephalitis can trigger anti-NMDA receptor encephalitis: Case report. *Neurology*. 2013;81(18):1637-1639. doi:10.1212/WNL.0b013e3182a9f531

Authors' Contributions

For peer review only

For peer review only - http://bmjopen.bmj.com/site/about/guidelines.xhtml

All authors were consulted and inputted into the article, below lists the particular role within DexEnceph

TW: Clinical research fellow, CF: Clinical research fellow, KD: trials manager, SD: contributor to trial design and running, MG: Clinical and laboratory biomarkers lead, CH: Neuropsychology researcher, RT: Trial pharmacist, GB: Trial statistician, AR-H: trial statistician, PM: Neuropsychology lead, KDas: Neuroimaging lead, MZ: Virology, LP: Neuroimaging, SK: Neuroimaging, NR: Neuroimaging, EA: Encephalitis Society Chief Executive advisor, ST: Study co-ordinator France, RK: Clinical lead brain infections UK, JPS: Principle investigator France, TS: Chief Investigator responsible for the trial

Trial Funding statement

This trial is funded by the NIHR Efficacy and Mechanism Evaluation Programme for the Department of Health reference 12/205/28.

Declaration of Interests

TS is supported by the National Institute for Health Research (NIHR) Health Protection Research Unit in Emerging and Zoonotic Infections (Grant No. IS-HPU-1112-10117), NIHR Global Health Research Group on Brain Infections (No. 17/63/110), and the European Union's Horizon 2020 research and innovation program ZikaPLAN (Preparedness Latin America Network), grant agreement No. 734584.

Other authors: non declared

Data Statement

There are currently no current plans to publish the data, data is available on reasonable request.

For peer review only

For peer review only - http://bmjopen.bmj.com/site/about/guidelines.xhtml

Word count: 4900 words

Figure Legends

Figure 1: Schematic design of DexEnceph randomised controlled trial

Figure 2 : DexEnceph Recruitment poster

Supplementary file 1: DexEnceph Information and Consent form

Table 1: Time scale for patients Randomised in the DexEnceph study

	Follow-up Schedule						
Procedures	Screening	Baseline	2 weeks	Discharge or Day 30 of admission (whichever is sooner)	26 weeks	78 weeks	Premature Discontinuation
Signed Consent Form	X†	X*					
Assessment of Eligibility Criteria	X	X*					
Review of Medical History		X*					
Review of Concomitant Medications		X*	X	X	X	X	X
Physical Exam		X		X			
Study Intervention		X					
Clinical Data Collection		X		X	X	X	
Magnetic resonance imaging (MRI) scan		Xμ	X		X	X	
Research Blood Testing		X	X		X		
Lumbar Puncture		Xγ	X				
Disability & Functional Outcomes				X	X	X	
Glasgow Coma Scale		X∞*	X∞	X∞	X	X	
Addenbrooke's Cognitive Examination revised				X	X	X	
Neuropsychology assessment					X	X	
Health Status and quality of life questionnaires					X	X	

For peer review only - http://bmjopen.bmj.com/site/about/guidelines.xhtml

	Follow-up Schedule						
Procedures	Screening	Baseline	2 weeks	Discharge or Day 30 of admission (whichever is sooner)	26 weeks	78 weeks	Premature Discontinuation
Clinical Laboratory: Haematology, Biochemistry		Xα					
Assessment of Adverse Events			(X)	(X)	(X)	(X)	(X)

† Only applicable when patients are prospectively consented for the randomised controlled trial
*Procedures required before randomisation.

μ Baseline MRI done for clinical purposes can be done from hospital admission up to 7 days after randomisation
γ Diagnostic lumbar puncture for clinical purposes done prior to randomisation
α Recording of clinic al laboratory tests done for clinical purposes, NOT as part of trial
∞ Recorded prior to randomisation, daily for the first 14 days and then weekly until Discharge/30 days (whichever sooner)
(X) – As indicated/appropriate

For peer review only

Figure 1:Schematic Design of DexEnceph randomised controlled trial

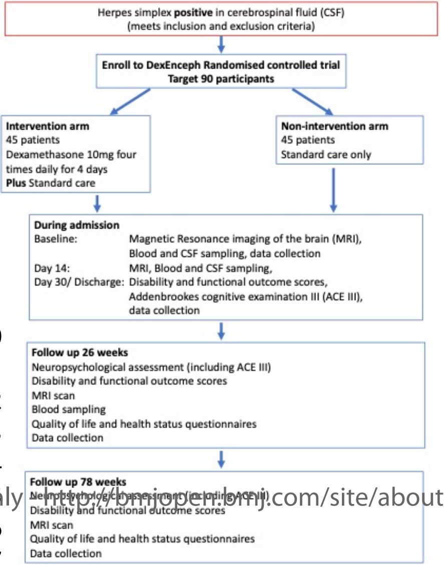

w only - http://bmjopen.bmj.com/site/about/gu

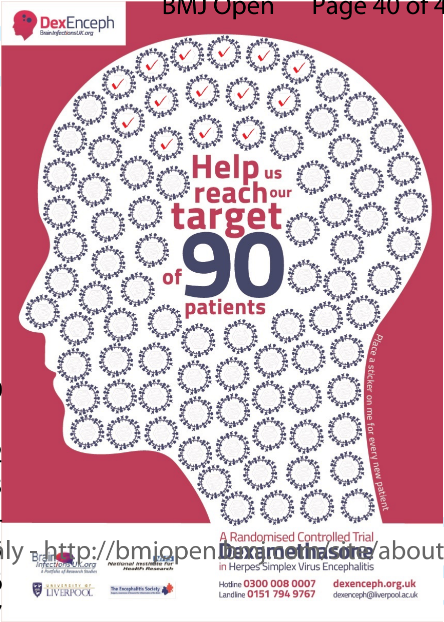

w only - http://bmjopen.bmj.com/site/about/gu

University of Liverpool
NHS National Institute for Health Research

DexEnceph
BrainInfectionsUK.org

RCT Adult with Capacity Information Sheet

Version 5.0, 25 May 2018

DexEnceph: A study of dexamethasone in adults with Herpes Simplex Virus (HSV) encephalitis
Brain Infections Group, University of Liverpool

We understand this is a difficult and stressful time for you, so we firstly want to thank you for taking the time to read this leaflet.

You are being invited to take part in a research study on HSV encephalitis. This condition is extremely rare and is probably something you had never heard about before. This is why a team member will go through this leaflet with you, explaining what taking part in the study would involve and answering any questions.

Important things you need to know

- This is a study for patients with encephalitis (swelling of the brain) caused by a virus called herpes simplex virus (HSV).
- Encephalitis can make you confused, drowsy, behave out of character, affect your sleep and memory, change your mood or may cause you to have fits.
- We want to find out if reducing the swelling with a drug called dexamethasone is of benefit to patient's memory in the longer term.
- In the study there will be two groups of patients, one that receives dexamethasone and one that does not.
- If you are in the group that receives dexamethasone this will be for 4 days in hospital.
- Both groups will have the same investigations to see if dexamethasone has been of benefit.
- Dexamethasone is a commonly used drug in brain swelling and many other conditions. Like all medicines, dexamethasone has side-effects. We will explain what these can be later.

We would like to invite you to take part in a research study

- Before you decide to take part it is important you know why the research is being done and what it will involve.
- You can discuss with family, friends and clinical staff before making a decision.
- You are free to decide whether you would like to take part.
- If you choose to take part and then decide you no longer want to be involved you can stop taking part without giving a reason. Your care will not be affected.
- Please let us know if there is anything in this leaflet that is not clear or if you would like more information. A member of our team will answer your questions.
- If you decide to take part we will offer you a copy of this form and ask you to sign a consent form.

For peer review only - http://bmjopen.bmj.com/site/about/guidelines.xhtml

UNIVERSITY OF LIVERPOOL NHS National Institute for Health Research

HSV encephalitis

1. What is HSV encephalitis?

Encephalitis means swelling of the brain and has many different causes. It is often caused by a virus. Herpes Simplex Virus (HSV) is the most common virus that causes encephalitis in the UK.

HSV encephalitis is very rare. It is diagnosed by finding the virus in fluid around the brain and spinal cord. This fluid is called CSF (cerebrospinal fluid). The CSF is obtained by the doctor who performs a lumbar puncture (LP).

HSV encephalitis is treated with the drug aciclovir. Despite treatment, some people are left with significant loss of memory. About 2 out of every 3 people will have memory difficulties long term.

The study

2. Why are we doing this study?

We know dexamethasone can reduce swelling. Reduction in swelling of the brain may improve the recovery of patients with HSV encephalitis.

This study, called DexEnceph, will allow us to compare the recovery of patients that received dexamethasone and those that did not.

3. Why have I been invited to take part?

There are two reasons why you may have been invited to take part:

A. Your doctors have diagnosed you with having HSV encephalitis.

OR

B. You may have been invited to take part before the diagnosis is made. This is because your doctors think there is a chance you may have HSV encephalitis. This will mean you have more time to think about taking part.

4. What will happen to me during the study?

All patients in the study will receive aciclovir. This is standard treatment for HSV encephalitis.

In addition, if you decide to take part in the study, you may be offered a short course of dexamethasone. This will be decided at random by a computer. This is to be fair, so neither you, your doctor, nor the research team, can choose whether you receive dexamethasone or not. Half of the people in the study will receive dexamethasone and half will not.

If you receive dexamethasone this will be 4 times a day for 4 days. It is given in a line you already have for clinical care.

What taking part involves

5. What tests are done if I take part?

When you sign up to the study:
Blood sample
CSF sample
MRI scan

↓

After 2 weeks:
MRI scan
Blood sample
CSF sample

↓

After 1 month (or when you are discharged from hospital):
Review of activities daily living
Short memory test only

↓

After 6 months:
Memory test
MRI scan
Blood sample
Review of activities daily living
Health questionnaires

↓

After 18 months:
Memory test
MRI scan
Review of activities daily living
Health questionnaires

All the tests done when you sign up to the study and the CSF tests after 2 weeks will

For peer review only - http://bmjopen.bmj.com/site/about/guidelines.xhtml

be done as part of your care whether you take part in the study or not.

6. What do the memory tests involve?

These tests are the most important in the study as they will help us find out if dexamethasone improves memory problems from HSV encephalitis. These tests are sometimes called Neuropsychology tests. They are completed 6 and 18 months after the illness.

The key part of this test takes about 35 minutes. If you are not too tired we can continue with further tests that will provide useful information. These can take up to 2 hours.

They are not pass or fail tests. They provide information about your memory and thinking processes.

They can be done in one day or divided over a few short visits. If you have left hospital we can travel to see you in a convenient place for you. The test will be arranged on a day(s) which suits you.

The results can be added to your hospital notes for future reference if you wish or kept confidential within the trial.

7. What does the MRI scan involve?

As part of your care your doctor will organise an MRI scan when you are in hospital. If you take part in the study we will ask you to have another 3 scans later on.

MRI scans allow us to assess if the brain has been affected by the infection and, if so, which parts.

Each scan takes about 20 minutes. The scan can be noisy but you will be offered headphones.

The extra 3 scans are planned for:

- 2 weeks after the first one (when you are still in hospital)
- After 6 months
- After 18 months

The scans will be done at a hospital near you. We will reimburse mileage or public transport costs for any research visits.

We will check with you that you are still happy to have the scan each time. Sometimes scans may find something not related to this illness. If this happens the doctors looking at the scans will tell your own hospital doctors who will look into this further.

None of the research scans are compulsory so if you do not wish to have them you can still be part of the study.

8. Are there risks to having an MRI scan?

There are no known risks from an MRI scan. They do not use radiation. MRI scans are done routinely in patients with HSV encephalitis.

Because MRI scans use strong magnets you will not have the scan if you have any metal implants or fragments in your body.

Where you lie is quite enclosed and some people may find this unsettling. If you have a fear of confined spaces you should discuss this with your doctor before you go for the scan.

If you think you may be pregnant let your local research team know. We will not ask pregnant women to have MRI scans due to possible risks to the foetus.

9. What samples are collected? What does this involve?

We will collect blood and CSF samples during the study.

All patients with HSV encephalitis need a lumbar puncture (LP) when they come to hospital to find out why they are unwell. The doctor uses a small needle to take a sample from the lower part of the back. This is repeated after 2 weeks of treatment to see if all the virus has gone. Both lumbar punctures are part of the standard care in all patients with this condition.

For peer review only - http://bmjopen.bmj.com/site/about/guidelines.xhtml

We will take a little extra fluid at this time for the research tests. The amount of fluid we ask for each time is about 1 teaspoon, 5.5mls.

If you have already had a lumbar puncture before being told about the study, we will take stored CSF that is leftover for research tests.

Blood tests are requested at 3 different times spread over 6 months. We take between 1 to 4 teaspoons of blood, this is 5 to 23mls.

With these blood and CSF tests we will be able to better understand how the infection affects your body and how the body tries to defend itself against it.

For peer review only

10. What will happen to the samples that are collected? Will any genetic tests be done?

All samples will be taken at your hospital and then transported to the University of Liverpool or other laboratories supporting the study. The samples will not have any of your personal information written on them. In the University they will be stored in a secure building.

There is an option for the blood and CSF collected to have tests looking at DNA. DNA is found in all cells of the body and contains the genetic information for the working of all human beings. This study collects DNA samples to find out why some people get HSV encephalitis and others do not, and why some people have severe problems due to HSV and others do not. The information we learn from DNA may benefit others with this condition in the future but will not influence your treatment or your future health.

Some of your samples may be left over. We will ask you if they can be used for this and future studies run by the University of Liverpool.

11. How do you review activities of daily living?

We will find out how the illness has affected your day-to-day life.

The research team will look at your hospital notes. They may also talk to you and, if you choose, your relatives. This will happen when you are in hospital and when you have gone home.

We will compare patients who received dexamethasone to those that did not and see if it made a difference.

12. What are the health questionnaires?

Two questionnaires will be sent through the post. They will ask you your views about your health and quality of life. Please send them back in a pre-paid envelope.

Dexamethasone

13. What are the side effects of dexamethasone?

Dexamethasone is used widely in patients and the side-effects are well known as this medicine has been prescribed for a long time. A short course of dexamethasone will be prescribed in this study. Side effects are less common when dexamethasone is given for shorter periods.

It is important you know about the possible side-effects before you decide to take part. These are:

- Stomach pain, indigestion, having more appetite than usual, feeling or being sick.
- Feeling tired or fatigued
- Mood and behaviour changes, especially at the beginning.
- Higher blood sugars.

Other possible risks can include:

- Stomach ulcers and bleeding of ulcers.
- Decreased response to infections.

For peer review only - http://bmjopen.bmj.com/site/about/guidelines.xhtml

You will be in hospital when you take dexamethasone so you can tell your doctors immediately if you have any problem.

If you suffer side effects you or your doctor can decide to stop the dexamethasone at any point.

Dexamethasone is prescribed to women who are pregnant or breast feeding as there are no known risks to the foetus.

Other things to consider

14. Do I have to take part?

No, taking part is voluntary. If you agree, we will ask you to sign a consent form.

If you agree to take part you are free to change your mind at any time, without giving a reason. You may decide to have only some tests in the study without having to drop out of the study altogether. This will not affect the standard of care you receive.

If you withdraw from the study we will stop collecting data. We will ask you if we can use the information and samples we have gathered up to the point that you withdraw.

15. What happens if there is a problem?

If you have any concerns about any part of this study, please speak with your hospital doctor (consultant) or one of your research team.

If you remain unhappy and wish to complain formally you can do this using the NHS Complaints Procedure. You can get information on how to do this from the Patients Advice Liaison department (PALS) in your hospital.

If you suffer harm from taking part in this study, there are no special compensation arrangements. If harm occurs to you and it is due to someone's negligence, you may have grounds for legal action for compensation against the NHS hospital where you are being treated but you may have to pay your legal costs.

16. Who will know I have taken part in this study?

Only people in your clinical care team and people involved in the study will have access to personal data. With your consent we will tell your GP that you are taking part.

All information collected about you during this study will be confidential and anonymised. It will be handled, stored and destroyed in accordance with the General Data Protection Regulation.

University of Liverpool is the sponsor for this study based in the United Kingdom. We will be using information from you and your medical records in order to undertake this study and will act as the data controller for this study. This means that we are responsible for looking after your information and using it properly. University of Liverpool will keep identifiable information about you for 15 years after the study has finished

Your rights to access, change or move your information are limited, as we need to manage your information in specific ways in order for the research to be reliable and accurate. If you withdraw from the study, we will keep the information about you that we have already obtained. To safeguard your rights, we will use the minimum personally-identifiable information possible.

You can find out more about how we use your information http://www.dexenceph.org.uk/. Our Data Protection Officer is Victoria Heath and you can contact them at V.Heath@liverpool.ac.uk.

Benefits and risks

17. What are the benefits of taking part?

You may benefit from receiving dexamethasone, however we will not know this until the end of the study. You

For peer review only - http://bmjopen.bmj.com/site/about/guidelines.xhtml

UNIVERSITY OF LIVERPOOL NHS National Institute for Health Research

may also benefit from the increased monitoring of having extra scans and memory tests.

The information we get from this study may benefit patients in the future.

18. What are the possible disadvantages and risks of taking part?

The disadvantage in taking part in this study may be the risk of having the side-effects of dexamethasone listed in question 13 (this will not be the case if you are in the group that does not have dexamethasone).

There is the inconvenience of having the dexamethasone through the drip when you are in hospital. Once you leave hospital there is the inconvenience of travelling to hospital for 2 scans, having the memory tests and completing questionnaires.

Contact details

If you have any questions about this study, then please contact the study team members:

Principal Investigator (Doctor leading this study in your hospital):

Name: ____________________________

____

Telephone:_________________________

Research Nurse:

Name: ____________________________

____

Telephone:_________________________

Name of your Hospital:_______________

____

Further information

This study is being run at your hospital and many other NHS hospitals throughout the UK. It aims to recruit 90 patients over 4 years.

It is organised by the University of Liverpool and is funded by the National Institute for Health Research (NIHR), the public body in charge of research in the UK.

Our study team includes The Encephalitis Society, a charity that supports patients and families (www.encephalitis.info).

The study has been reviewed for scientific content by expert members of NIHR. The National Research Ethics Service Committee Liverpool Central has reviewed the study and given approval for it to take place.

For peer review only

RCT Adult with Capacity Consent Form

Version 5.0, Dated: 25/May/2018

EudraCT Number: 2015-001609-16

Centre Name: Centre Code:

Name of Principal Investigator:

Study Number:

For peer review only

Please complete this form. When completed give one copy to the participant to keep, send one copy to CTU [fax/encrypted email/post], and keep one in the participant's medical notes. Please put the original in the site file.

**For patient: once you have understood each statement please initial the YES OR NO box**	**YES**	**NO**
1. I confirm I have read and understand the Information Leaflet (dated DD/MMM/YYYY) for the above study, and have had the opportunity to ask questions and have these answered satisfactorily.	INITIAL IF YES	INITIAL IF NO
2. I agree to take part in this study.	INITIAL IF YES	INITIAL IF NO
3. I understand that my participation is voluntary and that I am free to withdraw from the study at any time without giving a reason and without my care or legal rights being affected.	INITIAL IF YES	INITIAL IF NO
4. I agree for my consent form and contact details to be passed to the University of Liverpool for the administration of the study.	INITIAL IF YES	INITIAL IF NO
5. I understand that relevant sections of my medical notes and any data collected during the study may be looked at by authorised individuals from the research and clinical team and Regulatory Authorities where it is relevant to my taking part in this research. I give permission for these individuals to have access to my records.	INITIAL IF YES	INITIAL IF NO
6. I agree for genetic tests to be done on blood and CSF collected. I understand these genetic tests will not be of any individual significance to me.	INITIAL IF YES	INITIAL IF NO

For peer review only - http://bmjopen.bmj.com/site/about/guidelines.xhtml

7. I agree to have MRI scans as part of the trial.	INITIAL IF YES	INITIAL IF NO
8. I agree for my GP and hospital doctors to be informed if the scan picks up something unexpected.	INITIAL IF YES	INITIAL IF NO
9. I agree to gift the remainder of any blood or CSF sample to the University of Liverpool where it will be stored for use in future research. This may include genetic tests.	INITIAL IF YES	INITIAL IF NO
10. I agree to any images or scans that are taken to be used for teaching, education and publication (in scientific journals, books or internet).	INITIAL IF YES	INITIAL IF NO
11. I agree for my GP to be informed I am taking part in this study.	INITIAL IF YES	INITIAL IF NO

For peer review only

Name of Participant (Please print)	Signature	Date (DD/MM/YYYY)

Researcher*	Signature	Date (DD/MM/YYYY)

*** Important:** Prior to signing please ensure local research contact details are complete on page 6.

Information to Research Team:

Once a Consent Form has been signed, please copy three times: One for the participant, one to file in the medical notes and fax/post/encrypted email one to CTU. Please place original in the site file.

Please fax/encrypt email/post this consent form to CTU **separately** to other anonymised trial documents (e.g. CRF).

For peer review only - http://bmjopen.bmj.com/site/about/guidelines.xhtml

BMJ Open

Protocol for DexEnceph; a randomised controlled trial of dexamethasone therapy in adults with Herpes Simplex Virus Encephalitis

Journal:	*BMJ Open*
Manuscript ID	bmjopen-2020-041808.R2
Article Type:	Protocol
Date Submitted by the Author:	23-Feb-2021
Complete List of Authors:	Whitfield, Thomas; University of Liverpool, Brain Infections Unit<br>Fernandez, Cristina; University of Liverpool, Brain Infections Unit<br>Davies, Kelly; University of Liverpool, Clinical Trials Research Centre<br>Defres, Sylviane; Institute of Infection and Global Health, Clinical infection, medical mircobiology and Immunology; Royal Liverpool and Broadgreen University Hospitals Trust, Tropical infectious diseases Unit<br>Griffiths, Michael; University of Liverpool, Brain Infections Unit; Alder Hey Children's NHS Foundation Trust, Neurology<br>Hooper, Cory; University of Liverpool, Brain Infections Unit<br>Tangney, Rebecca; Aintree University Hospitals NHS Foundation Trust<br>Burnside, Girvan; University of Liverpool Faculty of Health and Life Sciences, Biostatistics<br>Rosala- Hallas, Anna; University of Liverpool Faculty of Health and Life Sciences, Biostatistics<br>Moore, Perry; The Walton Centre NHS Foundation Trust, Dept of Clinical Neuropsychology<br>Das, Kumar; The Walton Centre NHS Foundation Trust<br>Zuckerman, Mark; King's College Hospital NHS Foundation Trust, South London Specialist Virology Centre<br>Parkes, Laura; University of Manchester Institute of Science and Technology Department of Biomolecular Science,<br>Keller, Simon; Aintree University Hospitals NHS Foundation Trust<br>Roberts, Neil; The University of Edinburgh The Queen's Medical Research Institute<br>Easton, Ava; The Encephalitis Society<br>Touati, Saber; CHU Grenoble Alpes, Service des Maladies Infectieuses et Tropicales<br>Kneen, Rachel; Alder Hey Children's NHS Foundation Trust, Department of Neurology,; Institute of Infection and Global Health, Clinical Infection, medical microbiology and Immunology<br>Stahl, JP; Infectious Diseases Department, University of Grenoble<br>Solomon, Tom; University of Liverpool Institute of Infection and Global Health; National Institute for Health Research Health Protection Research Unit in Emerging and Zoonotic Infections, University of Liverpool
**Primary Subject Heading**:	Neurology
Secondary Subject Heading:	Infectious diseases, Pharmacology and therapeutics, Intensive care

Keywords:	Neurology < INTERNAL MEDICINE, VIROLOGY, NEUROLOGY, Neurological injury < NEUROLOGY

SCHOLARONE™
Manuscripts

I, the Submitting Author has the right to grant and does grant on behalf of all authors of the Work (as defined in the below author licence), an exclusive licence and/or a non-exclusive licence for contributions from authors who are: i) UK Crown employees; ii) where BMJ has agreed a CC-BY licence shall apply, and/or iii) in accordance with the terms applicable for US Federal Government officers or employees acting as part of their official duties; on a worldwide, perpetual, irrevocable, royalty-free basis to BMJ Publishing Group Ltd ("BMJ") its licensees and where the relevant Journal is co-owned by BMJ to the co-owners of the Journal, to publish the Work in this journal and any other BMJ products and to exploit all rights, as set out in our licence.

The Submitting Author accepts and understands that any supply made under these terms is made by BMJ to the Submitting Author unless you are acting as an employee on behalf of your employer or a postgraduate student of an affiliated institution which is paying any applicable article publishing charge ("APC") for Open Access articles. Where the Submitting Author wishes to make the Work available on an Open Access basis (and intends to pay the relevant APC), the terms of reuse of such Open Access shall be governed by a Creative Commons licence – details of these licences and which Creative Commons licence will apply to this Work are set out in our licence referred to above.

Other than as permitted in any relevant BMJ Author's Self Archiving Policies, I confirm this Work has not been accepted for publication elsewhere, is not being considered for publication elsewhere and does not duplicate material already published. I confirm all authors consent to publication of this Work and authorise the granting of this licence.

For peer review only

Protocol for DexEnceph; a randomised controlled trial of dexamethasone therapy in adults with Herpes Simplex Virus Encephalitis

The study is funded by the National Institute for Health Research's Efficacy and Mechanism Evaluation (EME) programme.

The trial sponsor is the University of Liverpool, who are responsible for all aspects of study design, implementation, analysis and write up (sponsor@liverpool.ac.uk).

For peer review only

Abstract

Introduction

Herpes simplex virus (HSV) encephalitis is a severe form of brain inflammation that commonly leaves survivors and their families with devastating long-term consequences. The virus particularly targets the temporal lobe of the brain causing debilitating memory problems, especially in verbal memory. It is postulated that immunomodulation with steroids could improve outcomes by reducing brain swelling. However, there are concerns (so far not observed) that such immunosuppression might facilitate increased viral replication with resultant worsening of disease. A previous trail closed early because of slow recruitment.

Method

DexEnceph is a pragmatic multicentre, randomised, controlled, open label, observer-blind trial to determine whether adults who receive dexamethasone alongside standard treatment with aciclovir for HSV encephalitis have improved clinical outcomes compared with those who receive standard treatment alone. Overall, 90 patients with HSV encephalitis will be recruited from a target of 90 recruiting sites; patients will be randomised 1:1 to the dexamethasone or control arms of the study. The primary outcome measured is verbal memory as assessed by the Weschler Memory Scale fourth edition Auditory Memory Index at 26 weeks post randomisation. Secondary outcomes are measured up to 72 weeks include additional neuropsychological, clinical and functional outcomes as well as comparison of neuroimaging findings. Patient safety monitoring occurs throughout and includes the detection of HSV DNA in cerebrospinal fluid 2 weeks after randomisation, which is indicative of ongoing viral replication.

For peer review only - http://bmjopen.bmj.com/site/about/guidelines.xhtml

Discussion

DexEnceph aims to be the first completed randomised controlled trial of steroid therapy in HSV encephalitis. The results will provide evidence for future practice in managing adults with the condition and has the potential to improve outcomes in a life-changing disease.

Ethics and dissemination

The trial has ethical approval from the UK National Research Ethics committee (Liverpool Central, REF: 15/NW/0545, 10/08/2015). Protocol version 2.1 July 2019 The results will be published and presented as soon as possible on completion.

Registration numbers

ISRCTN11774734, EUDRACT 2015-001609-16

Key words: Herpes simplex, Encephalitis, Dexamethasone, Steroid, Verbal memory

For peer review only

Article Summary

'Strengths and limitations of this study',

- DexEnceph will be the first completed randomised controlled trial of steroid in herpes simplex virus encephalitis, examining the utility and safety of steroid use.
- DexEnceph's primary end point is verbal memory score recorded at 26 weeks post randomisation, this represents the most common area of the brain to be affected.
- The recruitment target is informed by the recent Enceph-UK programme grant of encephalitis in the UK; the trial is currently open and has recruited 71 patients of a target 90.
- Concentration on site engagement with innovative, bespoke methods are key to identifying and recruiting patients to ensure trial success.
- The pragmatic open label, observer blind design of the trial is ensuring successful recruitment to date.

Authors: Whitfield T,[1] Fernandez C, [1] Davies K, [2] Defres S[1, 13, 15], Griffiths M, [1, 14] Hooper C,[1] Tangney R, [3] Burnside G, [4] Rosala-Hallas A,[4] Moore P, [12] Das K, [5] Zuckerman M, [6] Parkes L, [7], Keller S, [3] Roberts N,[8] Easton A, [1,9] Touati S,[10] Kneen R, [1,14] JP Stahl[11] & Solomon T[1, 5,15]

1. Institute of Infection and Global Health, University of Liverpool, L69 7BE, United Kingdom
2. Clinical Trials Research Centre, University of Liverpool, L12 2AP, United Kingdom
3. University Hospital Aintree, L9 7AL, United Kingdom
4. Department of Biostatistics, University of Liverpool, L69 3GL, United Kingdom

For peer review only

5. The Walton Centre NHS Foundation Trust, L9 7JL, United Kingdom
6. South London Specialist Virology Centre, Kings College Hospital NHS Foundation Trust, SE5 9RS, United Kingdom
7. Division of Neuroscience and Experimental Psychology, Faculty of Biology Medicine and Health, University of Manchester, M13 9NT, United Kingdom
8. Queens Medical Research Institute, University of Edinburgh, EH16 4TJ, United Kingdom
9. Encephalitis Society, Malton, North Yorkshire, YO17 7DT, United Kingdom
10. Département de Recherche Clinique (DRC), Grenoble Cedex, France
11. Infectious Diseases Department, University and Hospital Grenoble Alpes, Grenoble Cedex, France
12. Salford Royal NHS Foundation Trust, M6 8HD, United Kingdom
13. Tropical Infectious diseases Unit, Royal Liverpool University Hospital, L7 8XP, United Kingdom
14. Alder Hey NHS Foundation Trust, Liverpool, L14 5AB, United Kingdom
15. NIHR Health Protection Research Unit on Emerging and Zoonotic Infections, Liverpool, L69 7BE, United Kingdom

Correspondence & trial sponsor: Tom Solomon tsolomon@liverpool.ac.uk

HPRU Project Team, The Ronald Ross Building, University of Liverpool, 8 West Derby Street, Liverpool, L69 7BE

Tel: 0151 795 7552

Fax: 0151 795 5527

For peer review only

Word Count : 4900 words

Introduction

Herpes simplex virus infection (HSV) is the most commonly identified viral cause of encephalitis, inflammation and swelling of the brain caused by a virus or the body's immune system, in the UK as in most western industrialised nations.[1–4] The incidence has been estimated at 1 in 250,000-500,000,[2] with evidence this may be higher.[4] Although a rare disease, HSV encephalitis has a disproportionately large impact due to its devastating long term neuro-psychological sequelae . These can have a marked impact on the quality of life of the patient and their family and high health economic and social costs.[5,6]

Since the introduction of the antiviral drug aciclovir in the 1970s the mortality of HSV encephalitis has reduced from around 70% to 5.5-12%.[7–9] However, survivors are commonly left with neurological impairment; less than 20% of patients are able to return to work and 48% are classed as moderate to severely disabled.[10] Even when obvious disabilities have not occurred, families often report personality changes – the person they take home from hospital is simply not the same as the one before the illness.[6,11]

HSV encephalitis can cause a broad range of cognitive impairments, but impaired memory, especially verbal memory, is the most common and likely relates to the viral predilection for

For peer review only - http://bmjopen.bmj.com/site/about/guidelines.xhtml

the temporal lobe of the brain.[12,13] The verbal memory deficits manifest as difficulties remembering names of objects and people, as well as listening to and recalling spoken information such as conversations.[14,15] In addition to memory problems, difficulties in processing speed, concentration, language and executive function are also common amongst survivors of HSV encephalitis, along with fatigue, poor concentration, anxiety and depression.[7,16,17]

The pathogenic mechanisms in HSV encephalitis are not fully understood. The evidence suggests that in addition to direct viral pathogenesis, inflammation of the brain in response to the virus is a key component of the disease process.[18–21] This is supported by the observation that in the cerebrospinal fluid (CSF), higher levels of proinflammatory chemokines, especially monocyte chemotactic protein (MCP)-1, interferon γ and interleukin 6 (IL-6) are associated with a worse prognosis.[22,23] Poor prognosis is also associated with the extent of inflammation seen on neuroimaging,[24] and the degree of temporal lobe swelling is correlated with the severity of verbal memory impairment.[25]

Control of the inflammation in HSV encephalitis may improve outcome, as shown in mouse models of the disease.[26–28] Before the availability of aciclovir, corticosteroids were also used routinely as a treatment in humans with HSV encephalitis,[29,30] and more recently both cerebral oedema on imaging and cerebrospinal fluid IL-6 levels were shown to be reduced in patients given corticosteroids.[23] However, because corticosteroids cause immunosuppression which in theory facilitates increased viral replication, their role is uncertain.[31]

In other brain infections, including bacterial meningitis and tuberculous meningitis the benefit of corticosteroids has been demonstrated in large clinical trials.[32] For HSV encephalitis the

For peer review only - http://bmjopen.bmj.com/site/about/guidelines.xhtml

potential benefit of using corticosteroid as an adjunct to aciclovir therapy has been suggested from small case series and retrospective comparisons, but there has been no prospective randomised study reported.[33–38] One study, the German trial of Aciclovir and Corticosteroids in Herpes simplex virus Encephalitis (the GACHE trial) was stopped early because of poor recruitment rates.[39] However, there is clearly a need for a study to assess this question. The DexEnceph Study, a randomised controlled trial of dexamethasone in HSV encephalitis, aims to achieve this.

Trial design

DexEnceph is a pragmatic, multicentre, randomised, controlled, observer-blind trial to determine whether the addition of dexamethasone to standard aciclovir treatment improves clinical outcomes (in particular verbal memory score) for adults with HSV encephalitis. Additionally, neuroimaging and biomarkers will be assessed along with detection of HSV in the cerebrospinal fluid at 2 weeks post randomisation to monitor for difference in viral replication between the two groups.

Primary objective

To determine whether a short course of intravenous dexamethasone, in addition to standard care, improves verbal memory score in adults with HSV encephalitis at 26 weeks post treatment compared to standard care alone.

Secondary objectives

For peer review only

Secondary objectives include the following:

To determine whether dexamethasone therapy has an effect on other neuropsychological, cognitive, clinical, disability and functional outcomes in HSV encephalitis.

To assess the effect of dexamethasone therapy on brain swelling assessed by neuroimaging imaging.

To determine whether dexamethasone therapy affects clearance of HSV from cerebrospinal fluid, the emergence of anti-N-Methyl-D Aspartic acid (NMDA) receptor antibody or causes any changes in transcriptomic and proteomic profiling in the CSF and blood.

A more comprehensive list of measures is detailed in the outcomes section.

Methods and analysis

DexEnceph is an observer blind, open label, prospective, randomised, controlled trial of dexamethasone at 10mg four times daily for 4 days, versus no dexamethasone, in adults with HSV encephalitis.

Research setting

The trial is being conducted in up to 45 NHS trusts, with a recruitment target of 0-2 patients per site per year. A full list of sites involved in DexEnceph can be obtained from www.dexenceph.org.uk. The trail uses SPIRIT reporting guidelines,[40] (supplementary file 1).

Eligibility criteria

Inclusion Criteria

For peer review only

Enrolled patients fulfil all of the following criteria:

1. Suspected encephalitis defined as: new onset seizure or new focal neurological signs or alteration in consciousness, cognition, personality, or behaviour. Personality / behaviour change includes agitation, psychosis, somnolence, insomnia, catatonia, mood lability, altered sleep pattern.

2. A positive HSV DNA polymerase chain reaction (PCR) result from CSF, reported not more than 7 days prior to randomisation.

3. Receiving intravenous aciclovir administered as 10mg/kg three times daily or at a reduced dose if clinically indicated.

4. Age ≥ 16 years.

5. Written informed consent given by the patient or their legal representative[41]

Exclusion Criteria

Patients are excluded if they have any of the following:

1. Have received oral or injectable corticosteroid therapy in the 30 days prior to the day of admission to hospital. This does not apply to topical/ inhaled corticosteroids. [Patients who have received oral or injectable corticosteroid therapy AFTER their admission to hospital will not be excluded from the study if they consent to trial participation].

For peer review only - http://bmjopen.bmj.com/site/about/guidelines.xhtml

2. History of hypersensitivity to corticosteroids.

3. Immunosuppression secondary to:

a. Known HIV infection and CD4 white cell count under $200/mm^3$

b. Currently taking biologic therapy or other immunosuppressive agents [e.g. azathioprine, methotrexate, ciclosporin]

c. Previous solid organ transplant and currently on immunosuppression

d. Previous bone marrow transplant

e. Currently undergoing a course of chemotherapy or radiotherapy

f. Known primary immunodeficiency syndrome

g. Known current haematological malignancy

4. Pre-existing indwelling ventricular devices.

5. Peptic ulcer disease in the last 6 months, defined as a peptic ulcer seen at endoscopy or an upper gastrointestinal bleed causing a ≥ 2unit haemoglobin drop, in the last 6 months.

6. Antiretroviral regime containing rilpivirine as current treatment [Levels of rilpivirne are known to significantly decrease in co-administration with dexamethasone, a switch to a suitable alternative can facilitate trial entry].

Intervention

For peer review only

Participants are randomised in a 1:1 ratio to dexamethasone four times daily for four days alongside standard care, or standard care alone (Figure 1). Standard care includes intravenous aciclovir for a minimum of 14 days based on an ideal body weight at 10mg/kg every 8 hours, unless dose adjustment to account for renal impairment is necessary. Participating clinicians remain free to modify or discontinue the dexamethasone administration or to give alternative treatments at any stage, if this is judged to be in the best interest of the patient.

Participants assigned dexamethasone receive 10 mg equivalent of ordinary ward stock, prescribed by an authorised member of the local study team, given intravenously four times daily for four days (16 doses in total) starting within 24 hours of randomisation.

The University of Liverpool employs a clinical trials unit to be responsible for screening, and monitoring data collection, quality and completeness. As there is a low number of participants to be recruited, the trials unit are able to liaise regularly with each site following randomisation to ensure all follow up data are collected. Primary outcome is recorded by a centrally employed roving neuropsychologist, who collects the neuropsychological outcomes at 26 and 72 weeks.

For peer review only

Outcome measures

Primary Outcome

Verbal memory score, determined by the Wechsler Memory Scale 4th edition (WMS-IV) Auditory Memory Index, at 26 weeks after randomisation.

Secondary Outcomes

Other neuropsychological outcome measures [at 26 weeks and 78 weeks after randomisation]:

- Verbal memory score, determined by the WMS-IV, Auditory Memory Index, at 78 weeks after randomisation.
- Visual, Immediate and Delayed Memory by Indexes of the WMS-IV, processing speed and working memory subscales from the Wechsler Adult Intelligence Scale Fourth Edition.
- Higher executive function using the Trail Making Test.
- Anxiety and Depression symptom levels by the Beck Depression Inventory and Beck Anxiety Inventory.
- Subjective cognitive complaints using the Perceived Deficits Questionnaire.

Cognitive Outcome Measures [at discharge or 30 days if still in hospital, 26 weeks and 78 weeks]

For peer review only - http://bmjopen.bmj.com/site/about/guidelines.xhtml

- Addenbrooke's Cognitive Assessment III.

Clinical Outcomes

- Incidence of epilepsy.
- Time to hospital discharge.
- Requirement of high dependency unit or Intensive care unit admission up to 30 days post randomisation.
- Time taken to be free of ventilatory support for 14 days [if any].
- Time to reach maximum recorded Glasgow coma scale score.
- Survival.

Disability & Functional Outcomes [at discharge or 30 days if still in hospital, 26 weeks and 78 weeks]:

- Modified Rankin Score, Barthel Index, Liverpool Outcome Score and Glasgow Outcome Scale Extended.

Imaging Outcomes: Change from Baseline at 2 weeks, 26 weeks and 78 weeks

- Temporal lobe volume (as % of intra-cranial volume).
- Whole brain volume (as % of intra-cranial volume).
- Volume of affected region as seen on fluid-attenuated inversion recovery (FLAIR) image (as % of intra-cranial volume).
- Volume of affected region as seen on diffusion-weighted image (as % of intra-cranial volume).

Biomarker Outcomes:

- Transcriptomic and proteomic profiling on blood at convalescence (2 weeks and 26 weeks), compared to acute baselines, and on CSF at 2 weeks compared to acute baseline.

For peer review only

- Anti-NMDA receptor antibody testing at 26 weeks.

Safety Outcomes:

- Proportion of patients with detectable HSV in CSF by PCR at 2 weeks.

Health Status and Quality of Life [at 26 and 78 weeks]:

- Measured by the EuroQoL-5 Dimension-5 Level quality of life scale (EQ-5D-5L) and 36 item short form survey (SF-36) self-completed questionnaires.

Screening

The majority of potential patients are identified by the local research team through identifying patients with a relevant clinical presentation suspicious of HSV encephalitis and/or detection of HSV in a CSF sample. A screening log is completed for all potential patients. A strong link with the local laboratory is essential as a key factor in ensuring eligible patients are not missed by the investigative team. Investigators for the local research team include neurologists, infectious disease clinicians, microbiologist and virologists.

Because this is an uncommon disease, extra measures have been taken to try and maximise recruitment. On identifying a suitable patient, sites are able to contact the trial management team for intensive support via a dedicated telephone hotline, email, or an app. Short videos which explain the trial to patients, families and to health care workers also support recruitment. Every month, the trial management group monitor the screening reports of each site for the previous 3 months, to ensure they are actively looking for patients. Lower than expected screening is followed up by the central study team making contact with the study site to review their screening methodology and offer support.

For peer review only

Randomisation

Participants are randomised using a 24-hour secure web-based programme, which is centrally controlled by the clinical trials research centre. Designated members of the trial team at the site (detailed on the delegation of responsibilities log) are provided with a unique username and password which is required to access the web-based randomisation system. In the event of system failure, the patient can be randomised centrally electronically or through secure envelopes. Each participant is allocated a unique study number (randomisation number), the primary identifier for all the participants in this study.

The neuropsychologist collecting the primary outcome and other outcome assessors such as radiologists and those responsible for paper authorship are blinded to randomisation during the trial. Trial participants and local site study teams, as well as the trial manager and trial data manager at the clinical trials unit are aware of what treatments have been allocated. The independent data safety and monitoring committee (IDSMC) and statisticians have access to unblinded data grouped by intervention throughout the trial and make recommendations to the trial's steering committee who would only become unblinded in the event of a serious event.

Participant timeline

The time schedule for enrolment, interventions and assessments is given in table 1.

For peer review only

Statistical Considerations

Sample Size

The primary outcome variable is verbal memory , assessed as part of WMS-IV. In one published series of adults who survived HSV encephalitis, 19 of 22 had memory impairment evident at follow up, with verbal memory being most severely affected. [12] In that study the mean (standard deviation, SD) verbal memory score was 88.9 (18.9) compared with the population mean (SD) of 100.[15] This score can only be assessed in survivors, we estimate approximately 10% of patients in the trial will die before assessment of the primary outcome.[8,42,43] In instances where the death is judged to be associated with encephalitis the verbal memory score is recorded as 40, (the lowest possible value which would be obtained even where a patient recorded no recall of any of the items administered in the memory subtests). Where the cause of death is thought to be independent of having encephalitis, those patients will be recorded as lost to follow up. Similarly, for patients who are too unwell, due to encephalitis, to undergo the assessment, the score is recorded as 40. Decisions as to whether the reasons for death or non-completion of the measures were due to encephalitis will be made by an independent committee blinded to dexamethasone allocation. Adjusting the estimate of mean and standard deviation from survivors, to include the 10% of patients with the lowest possible value of 40, gives a total population mean of 84.8, with a standard deviation of 23.1. A final sample size of 36 participants per group allows us to detect a clinically meaningful difference of 15.5 on the verbal memory score with 80% power, at a two-sided significance level of 0.05. Allowing for up to 20% dropout gives an initial target sample size of 45 participants per group, for a total of 90.

For peer review only

For peer review only - http://bmjopen.bmj.com/site/about/guidelines.xhtml

Statistical analysis

For the primary outcome, participants are included in the analysis based on the intention-to-treat principle. Verbal memory score will be compared between groups using linear regression. The model will be adjusted for pre-specified variables which are judged to be potentially related to the outcome, including age and admission Glasgow coma scale score. No interim analysis is planned, but there is regular monitoring by the IDSMC.

As there may be some missing primary outcome data due to death, inability to complete the assessment, or loss to follow up, a sensitivity analysis will be carried out. All randomised patients will be included in this analysis.

For continuous secondary outcome variables, comparisons between groups will be analysed as per the primary outcome. The results for residual viral presence in the CSF at 2 weeks will be reported with a 95% confidence interval for the difference in proportions between groups. Time to event outcomes will be analysed using Kaplan-Meier curves, log rank tests and Cox Proportional Hazards models. Binary secondary outcomes will be analysed using logistic regression.

Trial Promotion and engagement

Considerable effort is maintained to keep the principal investigators, research nurses and the community engaged in the trial. The trial is being publicised using public forums, the Encephalitis Society website and newsletter, social media, patient journey articles and work with newspapers and TV. To promote site engagement, study days are arranged for research

For peer review only - http://bmjopen.bmj.com/site/about/guidelines.xhtml

teams to attend, along with scheduled research nurse teleconferences to allow ideas on maximising recruitment and updates on trial progress to be shared. Sites are also kept updated through our website (www.dexenceph.org.uk), newsletters and an innovative sticker chart, whereby a sticker is sent out to every site each time a patient is recruited (Figure 2). The Encephalitis Society are playing a key role in providing additional support to the patients and their families aside from their work for the trial.

For peer review only

Trial Closure

The end of the trial is defined to be the date on which data for all participants is frozen and data entry privileges are withdrawn from the trial database. The trial may be closed prematurely by the trial steering committee, on the recommendation of the IDSMC if there is sufficient evidence of risk to patient safety.

Pharmacovigilance

Oversight of the trial is provided by the trial steering committee, which meets at least annually to review trial progress, safety, and adverse events. The committee is also informed of any protocol changes by the clinical trial research unit.

The Medicines for Human Use (Clinical Trials) Regulations 2004 (SI 2004/1031) definitions are used for Adverse Event (AE), Adverse Reaction (AR), Unexpected Adverse Reaction (UAR), Serious Adverse Event (SAE), Serious Adverse Reaction (SAR) or Suspected Unexpected Serious Adverse Reaction (SUSAR).

Depending on the nature of the event the reporting procedures below are followed:

For peer review only - http://bmjopen.bmj.com/site/about/guidelines.xhtml

1. Serious adverse events occurring up to 30 days after randomisation are reported through a Serious Adverse Event Form (if serious) or in the 30 day/discharge case record forms (CRF) if they are a notable event (Positive PCR in CSF at 2nd lumbar puncture, gastrointestinal bleed, hyperglycaemia requiring change in medical management, opportunistic infections, unexpected/severe neuropsychiatric events).

2. Serious adverse events occurring after 30 days from randomisation are monitored through reporting in the CRFs with safety data collected in the 26 week and 78-week CRFs if serious.

The research investigator at each study site (or designated other) assesses all adverse events for seriousness, causality and severity. The Chief Investigator (or designated other) assesses all adverse drug reactions for expectedness from known side effects of the use of Dexamethasone.[44] All serious ARs, AEs and SUSARs occurring up to 30 days from randomisation (apart from death unless the investigator suspects causality) require reporting to the clinical trials unit, within 24 hours of the site becoming aware of the event. In the case of death of a patient causality will be assessed by the trial steering committee.

The clinical trials unit will notify the Medicines and Healthcare products Regulatory Agency and main research ethics committee of all SUSARs that occur during the study according to the following timelines; fatal and life-threatening within 7 days of notification and non-life threatening within 15 days. All investigators are informed of all SUSARs occurring throughout the study.

SAEs occurring after 30 days from randomisation are monitored by the clinical trials unit via the 26 week and 78week CRFs. These CRFs need to be received at the clinical trials research unit by 4 weeks after the 26 and 6 weeks after 78week time points.

For peer review only

For peer review only - http://bmjopen.bmj.com/site/about/guidelines.xhtml

Safety data is provided to the IDSMC, who are responsible for safeguarding the interests of trial participants and assessing the safety of the interventions during the trial; the IDSMC ensures action is taken as needed should they become aware of trends in reported AEs that raise safety concerns.

Trial Funding and Financial Arrangements

Contractual agreements are in place between the Sponsor and collaborating centres that describe financial arrangements. Trial participants are not paid to participate in the trial but are paid travel expenses for the follow up visits, estimated at £50 per visit. Payments to sites are made per site initiation but the bulk of payments are made per patient recruitment. Sites receive payment for: clinical time oversight, research nurse time, administrative support, magnetic resonance imaging scanning and pharmacy oversight.

Patient and public involvement

The Encephalitis Society was consulted and provided advice on the design of the trial and the difficulties participants and their families will likely encounter. The Chief Executive of The Encephalitis Society is a co-applicant on the grant application and a co-author on this paper.

The Encephalitis Society has also provided patient representatives at our trial steering committee and assisted in the production and dissemination of trial promotional materials. The Encephalitis Society will drive forward publication and dissemination of the trial findings among lay, therapeutic and health professionals through the use of web materials, newsletters and guides as well as at conferences and seminars in relation to encephalitis and

For peer review only

For peer review only - http://bmjopen.bmj.com/site/about/guidelines.xhtml

related fields. All patients and their family/carers will be acknowledged in any outputs from the trial. We also work with The Encephalitis Society on a programme of teaching events and produce guides for healthcare professionals and lay people.

In instances where trial participants and their families have ongoing difficulties the central study team seek help for them through the Encephalitis Society and appropriate specialists for further assistance.

Ethics and Dissemination

The trial falls within the remit of the EU Directive 2001/20/EC, transposed into UK law as the UK Statutory Instrument 2004 No 1031: Medicines for Human Use (Clinical Trials) Regulations 2004 as amended. This trial is registered with the MHRA and granted Clinical Trial Authorisation (CTA). The EUDRACT number for CTA reference is 2015-001609-16. Ethical approval has been obtained from a multi-centre research ethics committee familiar with the principals of the Mental Capacity Act 2005 guidance for sites in England and Wales and the Adults with Incapacity Act 2008 for sites in Scotland as the principals are relevant to a clinical trial of investigational medicinal products (CTIMP). Clinical Research Governance approval was given through the Sponsor, The University of Liverpool. The trial protocol was approved by a National Research Ethics Service Committee reference is 2015-001609-16 (Attained 31/03/2016) and underwent independent review at the Research and Development offices at participating sites. This study abides by the principles of the World Medical Association Declaration of Helsinki (1964) and Tokyo (1975), Venice (1983), Hong Kong (1989) and South

For peer review only

For peer review only - http://bmjopen.bmj.com/site/about/guidelines.xhtml

Africa (1996). Due to the nature of this trial it also abides by the Medicine for Human Use (Clinical Trials) regulations 2004 (S.I.2004:1031) and all following amendments which are incorporated into UK law.

Informed Consent Process

In obtaining and documenting informed consent, the investigators adhere to National Institute for Health Research (NIHR) Good Clinical Practice (GCP) guidelines and the ethical principles derived from the Declaration of Helsinki. Staff delegated by the principal investigator and appropriately trained with experience in obtaining informed consent, discuss the objectives, risks and inconveniences of the trial and the conditions under which it is to be conducted with the patient or if the patient lacks capacity with a legal or professional representative. Trial information documents and points of contact for further information are provided and the potential participants are given adequate time to consider their decision(supplementary file 2).

As this is a CTIMP, the clinical trial regulations for incapacitated adults are followed (Medicines for Human Use Clinical Trial Regulations 2004 and amendments). When a legal representative has given consent for a patient to participate in the trial and the patient subsequently regains capacity, the research team will provide the patient information sheet and request consent from the participant. Patients are allowed to withdraw from the study at any point and may request withdrawal of their data collected until this point. Prospective consent can also be obtained prior to a positive PCR result so participants may have adequate time for contemplation.

For peer review only - http://bmjopen.bmj.com/site/about/guidelines.xhtml

As suspected encephalitis is a medical emergency, a deferred consent process is used for the collection and retention of some samples as part of routine clinical management. Using emergency deferred consent for samples involves taking additional samples of blood and CSF only if the procedure is being performed for clinical care. If deferred consent has been used, written consent is requested from either the patient or a legal representative as soon as is possible and appropriate, with samples discarded if this is declined. This approach is based on discussions with patients and the public through the Encephalitis Society.

Data Capture Methods

Data is stored securely in line with the Data Protection Act 1998. The randomisation system, data capture form and CRF have been designed to optimally protect participant information and to maintain confidentiality. Trial data is captured at local sites using paper CRFs. These are then sent into the clinical trials research unit for data entry into the study specific database. Completed CRFs are returned to clinical trial research centre within 7 days of completion. A copy of the CRF sent over to the clinical trials research unit is retained at site. CRFs and consent forms are stored separately and securely at all times in dedicated areas of the clinical trials research unit.

CRFs are checked for data quality by the clinical trials research unit in Liverpool responsible for ensuring data collection and storage.

Patients' anonymised and labelled neuroimaging data are put on to discs at site and sent to the clinical trials research unit; the images can also be transferred via the Image Exchange

For peer review only

Portal in an encrypted manner. The final dataset will be solely accessible to the central study team at the University of Liverpool for analysis and write up.

Dissemination

The results of the DexEnceph trial will be published in a high impact journal in a timely manner to present the findings to front-line clinicians. They will be presented at the annual conference organised by the Encephalitis Society and at other meetings. Authorship of the final papers will be determined in accordance with the international committee of medical journal editors' guidelines. The investigators will be involved in the preparation and drafting of the manuscripts. There is no intended use of professional writers.

Discussion

This protocol describes the design of a randomised controlled trial to demonstrate the utility of using dexamethasone in the management of individuals with HSV encephalitis. HSV encephalitis is a rare sporadic acute disease, and the trial has been designed to take this challenge into account, along with the practicalities of running the trial in a UK National Health Service setting. In particular lessons were learnt from a previous similar European study, the German trial of Aciclovir and Corticosteroids in Herpes simplex virus Encephalitis (GACHE trial), which was stopped early because of recruitment difficulties. Recruitment to the GACHE

For peer review only - http://bmjopen.bmj.com/site/about/guidelines.xhtml

trial necessitated patients had focal neurological signs of no longer than five days prior to admission, whilst DexEnceph has less stringent criteria and reflects the diverse ways in which HSV encephalitis may present. DexEnceph has been designed to be both practical and pragmatic, in that patients must be recruited within 7 days of the PCR result becoming available. This allows for occasions where it may take longer to get the PCR performed, and also allows time for patients admitted to district general hospitals, which may not be study centres, to be transferred to larger hospitals which are. DexEnceph also has the advantage in that its recruitment projections were based on preliminary data garnered from the ENCEPH-UK NIHR program (www.encephuk.org) and from a multicentre cohort study of encephalitis in England, run by the Health Protection Agency (fore-runner to the Health Protection Agency).[9] These two studies provided direct information on the number of HSV encephalitis patients presenting to UK hospitals. Our choice of an open label observer-blind study, rather than a placebo-controlled double-blind trial, avoided the logistic challenges of ensuring blinded study drug was available across the large number of centres, necessitated by a rare sporadic disease, which may also have been a factor in the recruitment difficulties experienced by the GACHE trial. We are confident our robust monitoring and trial promotion ensures the majority of eligible patients are recruited. N- methyl-D-aspartic acid (NMDA) receptor antibody encephalitis (which is treated with corticosteroids and other immunomodulatory therapies) is being recognised increasingly as a late complication of HSV encephalitis.[45,46] DexEnceph may also be able examine whether corticosteroids reduce the incidence of this complication. If there is demonstrable efficacy of corticosteroid in improving neuropsychological, imaging and quality of life outcomes, without compromising patient safety the results will be far reaching.

For peer review only

For peer review only - http://bmjopen.bmj.com/site/about/guidelines.xhtml

581

582 **Collaboration with France**

583 Because we recognised from the start that there may be difficulties keeping to recruitment
584 targets in the DexEnceph study, we worked with colleagues in France to develop a parallel
585 French study (DexEnceph-France). This follows the UK DexEnceph protocol as closely as
586 possible, whilst being pragmatic about the constraints of a different country's health care
587 system. The French trial is based in 10 hospitals with the lead centre being Grenoble Alpes
588 University Hospital and the aim of recruiting 30 patients.

589 The intention is for the two trials to be analysed separately, with the option of also results
590 also combining them into an overall analysis which will give additional power to detect a
591 treatment effect.

592

593

594

595

596 **Trial status**

597 The trial has been open across the UK since August 2016, and as of July 2019 is open at 45
598 NHS trusts; 71 patients have been randomised (of the total target of 90) meaning that we
599 have recruited 79% of the patients are recruiting at just over 80% of the target recruitment
600 rate. The trial was due to complete recruitment later in 2020 with 6 months follow up and 3
601 months post trial closure for write up. Though recent COVID-19 pandemic has placed
602 difficulties in conducting trials and paused recruitment, the primary outcome has not been
603 missed in any existing DexEnceph recruit, this has been achieved by conducting

For peer review only

neuropsychology assessment by either telephone or online video discussion, thus avoiding any risk of transmission. DexEnceph-France study, which opened in 2018 has recruited 10 patients from 10 sites.

If you are interested to find out more information or to see if your trust is involved visit www.dexenceph.org.uk or for more information please email: dexenceph@liverpool.ac.uk.

References

1. Davison KL, Crowcroft NS, Ramsay ME, Brown DWG, Andrews NJ. Viral encephalitis in England, 1989-1998: What did we miss? *Emerg Infect Dis*. 2003;9(2):234-240. doi:10.3201/eid0902.020218
2. Whitley RJ. Herpes simplex encephalitis: Adolescents and adults. *Antiviral Res*. 2006;71(2-3 SPEC. ISS.):141-148. doi:10.1016/j.antiviral.2006.04.002
3. Mailles A, Stahl J. Infectious Encephalitis in France in 2007: A National Prospective Study. *Clin Infect Dis*. 2009;49(12):1838-1847. doi:10.1086/648419
4. Granerod J, Cousens S, Davies NWS, Crowcroft NS, Thomas SL. New estimates of incidence of encephalitis in England. *Emerg Infect Dis*. 2013;19(9):1455-1462. doi:10.3201/eid1909.130064
5. Solomon T, Hart IJ, Beeching NJ, Solomon T, Hart IJ, Beeching NJ. Viral encephalitis : a

For peer review only

clinician ' s guide. 2007;(October):288-305. doi:10.1136/jnnp.2007.129098

6. Easton A. Encephalitis: raising awareness and collaborating in research. *Lancet Neurol*. 2016;15(4):353. doi:10.1016/s1474-4422(16)00059-4

7. Mcgrath N, Anderson NE, Croxson MC, Powell KF. Herpes simplex encephalitis treated with acyclovir: diagnosis and long term outcome. *J Neurol Neurosurg Psychiatry*. 1997;63:321-326. doi:10.1136/jnnp.63.3.321

8. Jouan Y, Grammatico-Guillon L, Espitalier F, Cazals X, François P, Guillon A. Long-term outcome of severe herpes simplex encephalitis: a population-based observational study. *Crit Care*. 2015;19(1):345. doi:10.1186/s13054-015-1046-y

9. Granerod J, Ambrose HE, Davies NWS, et al. Causes of encephalitis and differences in their clinical presentations in England: A multicentre, population-based prospective study. *Lancet Infect Dis*. 2010;10(12):835-844. doi:10.1016/S1473-3099(10)70222-X

10. Raschilas F, Wolff M, Delatour F, et al. Outcome of and Prognostic Factors for Herpes Simplex Encephalitis in Adult Patients: Results of a Multicenter Study. *Clin Infect Dis*. 2002;35(3):254-260. doi:10.1086/341405

11. Egdell R, Egdell D, Solomon T. Herpes simplex virus encephalitis. *Bmj*. 2012;344(jun13 2):e3630-e3630. doi:10.1136/bmj.e3630

12. Utley TF, Ogden JA, Gibb A, McGrath N, Anderson NE. The long-term neuropsychological outcome of herpes simplex encephalitis in a series of unselected survivors. *Neuropsychiatry Neuropsychol Behav Neurol*. 1997;10(3):180-189. http://www.ncbi.nlm.nih.gov/pubmed/9297711.

13. Frisch S, Thiel F, Marschhauser A, Villringer A, Horstmann A, Schroeter ML. Identifying neural correlates of memory and language disturbances in herpes simplex encephalitis: a voxel-based morphometry (VBM) study. *J Neurol*. 2015;262(3):563-

For peer review only

For peer review only - http://bmjopen.bmj.com/site/about/guidelines.xhtml

569. doi:10.1007/s00415-014-7604-4

14. Pewter SM, Williams WH, Haslam C, Kay JM. Neuropsychological and psychiatric profiles in acute encephalitis in adults. *Neuropsychol Rehabil*. 2007;17(4-5):478-505. doi:10.1080/09602010701202238

15. Hokkanen L, Poutiainen E, Valanne L, et al. Cognitive impairment after acute encephalitis: comparison of herpes simplex and other aetiologies. *Neurosurgery, and Psychiatry*. 1996;61(December 1994):478-484. doi:10.1136/jnnp.61.5.478

16. Cooper J, Kierans C, Defres S, Easton A, Kneen R, Solomon T. Care beyond the hospital ward: understanding the socio-medical trajectory of herpes simplex virus encephalitis. *BMC Health Serv Res*. 2017;17(1):1-11. doi:10.1186/s12913-017-2608-2

17. Cooper J, Kierans C, Defres S, Easton A, Kneen R, Solomon T. Diagnostic pathways as social and participatory practices: The case of herpes simplex encephalitis. *PLoS One*. 2016;11(3):1-15. doi:10.1371/journal.pone.0151145

18. Aurelius E, Andersson B, Forsgren M, Sköldenberg B, Strannegård. Cytokines and other markers of intrathecal immune response in patients with herpes simplex encephalitis. *J Infect Dis*. 1994;170(3):678-681. doi:10.1093/infdis/170.3.678

19. Sköldenberg B, Aurelius E, Hjalmarsson A, et al. Incidence and pathogenesis of clinical relapse after herpes simplex encephalitis in adults. *J Neurol*. 2006;253(2):163-170. doi:10.1007/s00415-005-0941-6

20. Michael BD, Griffiths MJ, Granerod J, et al. Characteristic Cytokine and Chemokine Profiles in Encephalitis of Infectious, Immune-Mediated, and Unknown Aetiology. *PLoS One*. 2016;11(1):e0146288. doi:10.1371/journal.pone.0146288

21. Wnęk M, Ressel L, Ricci E, et al. Herpes simplex encephalitis is linked with selective mitochondrial damage; a post-mortem and in vitro study. *Acta Neuropathol*.

For peer review only

For peer review only - http://bmjopen.bmj.com/site/about/guidelines.xhtml

676 2016;132(3):433-451. doi:10.1007/s00401-016-1597-2

677 22. Rösler A, Pohl M, Braune HJ, Oertel WH, Gemsa D, Sprenger H. Time course of
678 chemokines in the cerebrospinal fluid and serum during herpes simplex type 1
679 encephalitis. *J Neurol Sci*. 1998;157(1):82-89. doi:10.1016/S0022-510X(98)00061-6

680 23. Kamei S, Taira N, Ishihara M, et al. Prognostic value of cerebrospinal fluid cytokine
681 changes in herpes simplex virus encephalitis. *Cytokine*. 2009;46(2):187-193.
682 doi:10.1016/j.cyto.2009.01.004

683 24. Taira N, Kamei S, Morita a, et al. Predictors of a prolonged clinical course in adult
684 patients with herpes simplex virus encephalitis. *Intern Med*. 2009;48(2):89-94.
685 doi:10.2169/internalmedicine.48.1445

686 25. Kapur N, Barker S, Burrows EH, et al. Herpes simplex encephalitis: long term magnetic
687 resonance imaging and neuropsychological profile. *Neurol Neurosurg Psychiatry*.
688 1994;5757:1334-1342. doi:10.1136/jnnp.57.11.1334

689 26. Meyding-Lamadé UK, Oberlinner C, Rau PR, et al. Experimental herpes simplex virus
690 encephalitis: a combination therapy of acyclovir and glucocorticoids reduces long-
691 term magnetic resonance imaging abnormalities. *J Neurovirol*. 2003;9(1):118-125.
692 doi:10.1080/13550280390173373

693 27. Sellner J, Dvorak F, Zhou Y, et al. Acute and long-term alteration of chemokine mRNA
694 expression after anti-viral and anti-inflammatory treatment in herpes simplex virus
695 encephalitis. *Neurosci Lett*. 2005;374(3):197-202. doi:10.1016/j.neulet.2004.10.054

696 28. Meyding-lamade U, Seyfer S, Dvorak F, et al. Experimental herpes simplex virus
697 encephalitis: inhibition of the expression of inducible nitric oxide synthase in mouse
698 brain tissue. *Neurosci Lett*. 2002;318:21-24.

699 29. Upton AR., Barwick D., Foster J. DEXAMETHASONE TREATMENT IN HERPES-SIMPLEX

For peer review only

ENCEPHALITIS. *Lancet*. 1971;297(7693):290-291. doi:10.1016/S0140-6736(71)91019-1

30. Habel AH, Brown JK. DEXAMETHASONE IN HERPES-SIMPLEX ENCEPHALITIS. *Lancet*. 1972;299(7752):695. doi:10.1016/S0140-6736(72)90505-3

31. Defres S, Keller SS, Das K, et al. A feasibility study of quantifying longitudinal brain changes in herpes simplex virus (HSV) encephalitis using magnetic resonance imaging (MRI) and stereology. *PLoS One*. 2017;12(1):1-15. doi:10.1371/journal.pone.0170215

32. de Gans J, van de Beek D. Dexamethasone in Adults with Bacterial Meningitis. *N Engl J Med*. 2002;347(20):1549-1556. doi:10.1056/NEJMoa021334

33. Kamei S. Evaluation of combination therapy using aciclovir and corticosteroid in adult patients with herpes simplex virus encephalitis. *J Neurol Neurosurg Psychiatry*. 2005;76(11):1544-1549. doi:10.1136/jnnp.2004.049676

34. Musallam B, Matoth I, Wolf DG, Engelhard D, Averbuch D. Steroids for Deteriorating Herpes Simplex Virus Encephalitis. *Pediatr Neurol*. 2007;37(3):229-232. doi:10.1016/j.pediatrneurol.2007.05.007

35. Maraş Genç H, Uyur Yalçın E, Sayan M, et al. Clinical outcomes in children with herpes simplex encephalitis receiving steroid therapy. *J Clin Virol*. 2016;80:87-92. doi:10.1016/j.jcv.2016.05.002

36. Nakano A, Yamasaki R, Miyazaki S, Horiuchi N, Kunishige M, Mitsui T. Beneficial effect of steroid pulse therapy on acute viral encephalitis. *Eur Neurol*. 2003;50(4):225-229. doi:10.1159/000073864

37. Lizarraga KJ, Alexandre LC, Ramos-Estebanez C, Merenda A. Are steroids a beneficial adjunctive therapy in the immunosuppressed patient with herpes simplex virus encephalitis? *Case Rep Neurol*. 2013;5(1):52-55. doi:10.1159/000350572

38. Mesker AJ, Bon GG, de Gans J, de Kruijk JR. Case report: a pregnant woman with

For peer review only

herpes simplex encephalitis successfully treated with dexamethasone. *Eur J Obstet Gynecol Reprod Biol*. 2011;154(2):231-232. doi:10.1016/j.ejogrb.2010.10.014

39. Martinez-Torres F, Menon S, Pritsch M, et al. Protocol for German trial of Acyclovir and corticosteroids in Herpes-simplex-virus-encephalitis (GACHE): a multicenter, multinational, randomized, double-blind, placebo-controlled German, Austrian and Dutch trial [ISRCTN45122933]. *BMC Neurol*. 2008;8:40. doi:10.1186/1471-2377-8-40

40. Hro A, Mann H, Dickersin K, et al. Research and Reporting Methods Annals of Internal Medicine SPIRIT 2013 Statement : Defining Standard Protocol Items for Clinical Trials. *Ann Intern Med*. 2015;1(3):200-207. doi:10.7326/0003-4819-158-3-201302050-00583.Requests

41. The Medicines for Human Use (Clinical Trials) Regulations 2004. http://www.legislation.gov.uk/uksi/2004/1031/schedule/1/made. Accessed June 19, 2019.

42. Whitley RJ, Alford CA, Hirsch MS, et al. Vidarabine versus Acyclovir Therapy in Herpes Simplex Encephalitis. *N Engl J Med*. 1986;314(3):144-149. doi:10.1056/NEJM198601163140303

43. Sköldenberg B, Alestig K, Burman L, et al. ACYCLOVIR VERSUS VIDARABINE IN HERPES SIMPLEX ENCEPHALITIS. *Lancet*. 1984;324(8405):707-711. doi:10.1016/S0140-6736(84)92623-0

44. Spc. Dexamethasone SPC. 2014;(June):1-4.

45. Gilbert GJ, Leypoldt F, Dalmau J. Herpes simplex virus-1 encephalitis can trigger anti-NMDA receptor encephalitis: Case report. *Neurology*. 2014;82(22):2041-2041. doi:10.1212/01.wnl.0000450946.75616.32

46. Leypoldt F, Titulaer MJ, Aguilar E, et al. Herpes simplex virus-1 encephalitis can trigger

For peer review only

anti-NMDA receptor encephalitis: Case report. *Neurology*. 2013;81(18):1637-1639. doi:10.1212/WNL.0b013e3182a9f531

Authors' Contributions

All authors were consulted and inputted into the article, below lists the particular role within DexEnceph

TW: Clinical research fellow, CF: Clinical research fellow, KD: trials manager, SD: contributor to trial design and running, MG: Clinical and laboratory biomarkers lead, CH: Neuropsychology researcher, RT: Trial pharmacist, GB: Trial statistician, AR-H: trial statistician, PM: Neuropsychology lead, KDas: Neuroimaging lead, MZ: Virology, LP: Neuroimaging, SK: Neuroimaging, NR: Neuroimaging, EA: Encephalitis Society Chief Executive advisor, ST: Study co-ordinator France, RK: Clinical lead brain infections UK, JPS: Principle investigator France, TS: Chief Investigator responsible for the trial

Trial Funding statement

This trial is funded by the NIHR Efficacy and Mechanism Evaluation Programme for the Department of Health reference 12/205/28.

Declaration of Interests

TS is supported by the National Institute for Health Research (NIHR) Health Protection Research Unit in Emerging and Zoonotic Infections (Grant No. IS-HPU-1112-10117), NIHR Global Health Research Group on Brain Infections (No. 17/63/110), and the European Union's Horizon 2020 research and innovation program ZikaPLAN (Preparedness Latin America Network), grant agreement No. 734584.

For peer review only

Other authors: non declared

Data Statement

There are currently no current plans to publish the data, data is available on reasonable request.

Word count: 4900 words

Figure Legends

Figure 1: Schematic design of DexEnceph randomised controlled trial

Figure 2 : DexEnceph Recruitment poster

Supplementary file 1: DexEnceph SPIRIT checklist

Supplementary file 2: DexEnceph Information and Consent form

Table 1: Time scale for patients Randomised in the DexEnceph study

	Follow-up Schedule						
Procedures	Screening	Baseline	2 weeks	Discharge or Day 30 of admission (whichever is sooner)	26 weeks	78 weeks	Premature Discontinuation
Signed Consent Form	X†	X*					
Assessment of Eligibility Criteria	X	X*					
Review of Medical History		X*					
Review of Concomitant Medications		X*	X	X	X	X	X
Physical Exam		X		X			
Study Intervention		X					
Clinical Data Collection		X		X	X	X	

For peer review

For peer review only - http://bmjopen.bmj.com/site/about/guidelines.xhtml

	Follow-up Schedule						
Procedures	Screening	Baseline	2 weeks	Discharge or Day 30 of admission (whichever is sooner)	26 weeks	78 weeks	Premature Discontinuation
Magnetic resonance imaging (MRI) scan		Xμ	X		X	X	
Research Blood Testing		X	X		X		
Lumbar Puncture		Xγ	X				
Disability & Functional Outcomes				X	X	X	
Glasgow Coma Scale		X∞*	X∞	X∞	X	X	
Addenbrooke's Cognitive Examination revised				X	X	X	
Neuropsychology assessment					X	X	
Health Status and quality of life questionnaires					X	X	
Clinical Laboratory: Haematology, Biochemistry		Xα					
Assessment of Adverse Events			(X)	(X)	(X)	(X)	(X)

† Only applicable when patients are prospectively consented for the randomised controlled trial
*Procedures required before randomisation.

μ Baseline MRI done for clinical purposes can be done from hospital admission up to 7 days after randomisation
γ Diagnostic lumbar puncture for clinical purposes done prior to randomisation
α Recording of clinic al laboratory tests done for clinical purposes, NOT as part of trial
∞ Recorded prior to randomisation, daily for the first 14 days and then weekly until Discharge/30 days (whichever sooner)
(X) – As indicated/appropriate

review only

For peer review only

For peer review only - http://bmjopen.bmj.com/site/about/guidelines.xhtml

Figure 1:Schematic Design of DexEnceph randomised controlled trial

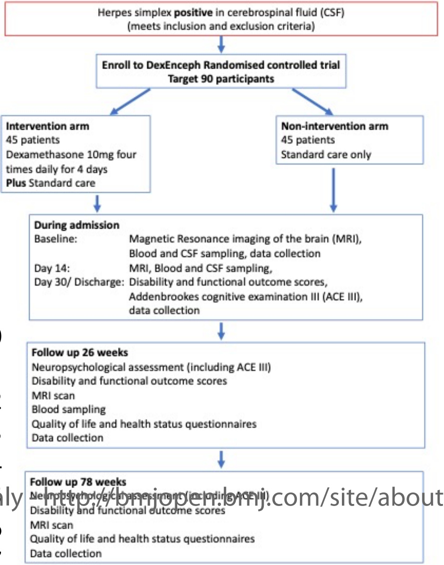

w only - http://bmjopen.bmj.com/site/about/gu

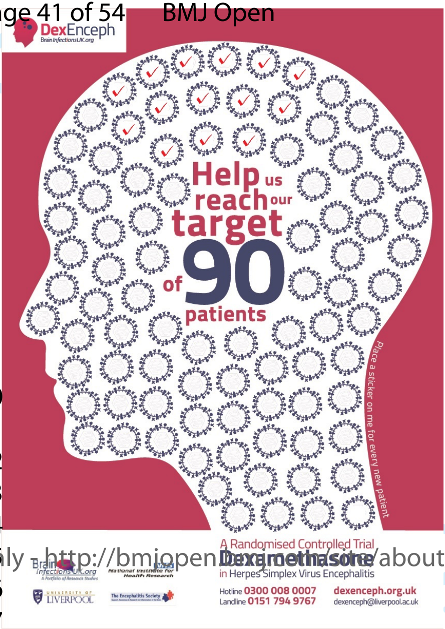

w only - http://bmjopen.bmj.com/site/about/gu

SPIRIT checklist for protocol of the DexEnceph clinical trial.

		Reporting Item	Page Number
**Administrative information**			
Title	#1	Descriptive title identifying the study design, population, interventions, and, if applicable, trial acronym	1
Trial registration	#2a	Trial identifier and registry name. If not yet registered, name of intended registry	3
Trial registration: data set	#2b	All items from the World Health Organization Trial Registration Data Set	3
Protocol version	#3	Date and version identifier	3
Funding	#4	Sources and types of financial, material, and other support	34
Roles and responsibilities: contributorship	#5a	Names, affiliations, and roles of protocol contributors	4-5
Roles and responsibilities: sponsor contact information	#5b	Name and contact information for the trial sponsor	1, 5
Roles and responsibilities: sponsor and funder	#5c	Role of study sponsor and funders, if any, in study design; collection, management, analysis, and interpretation of data; writing of the report; and the decision to submit the report for publication, including whether they will have ultimate authority over any of these activities	33-34

For peer review only

Roles and responsibilities: committees	#5d	Composition, roles, and responsibilities of the coordinating centre, steering committee, endpoint adjudication committee, data management team, and other individuals or groups overseeing the trial, if applicable (see Item 21a for data monitoring committee)	19-21
**Introduction**			
Background and rationale	#6a	Description of research question and justification for undertaking the trial, including summary of relevant studies (published and unpublished) examining benefits and harms for each intervention	6-9
Background and rationale: choice of comparators	#6b	Explanation for choice of comparators	6-9
Objectives	#7	Specific objectives or hypotheses	8-9
Trial design	#8	Description of trial design including type of trial (eg, parallel group, crossover, factorial, single group), allocation ratio, and framework (eg, superiority, equivalence, non-inferiority, exploratory)	8
**Methods: Participants, interventions, and outcomes**			
Study setting	#9	Description of study settings (eg, community clinic, academic hospital) and list of countries where data will be collected. Reference to where list of study sites can be obtained	9
Eligibility criteria	#10	Inclusion and exclusion criteria for participants. If applicable, eligibility criteria for study centres and individuals who will perform the interventions (eg, surgeons, psychotherapists)	9-11
Interventions: description	#11a	Interventions for each group with sufficient detail to allow replication, including how and when they will be administered	11-12

For peer review only

Interventions: modifications	#11b	Criteria for discontinuing or modifying allocated interventions for a given trial participant (eg, drug dose change in response to harms, participant request, or improving / worsening disease)	11-12, 20-21
Interventions: adherance	#11c	Strategies to improve adherence to intervention protocols, and any procedures for monitoring adherence (eg, drug tablet return; laboratory tests)	12, 18,
Interventions: concomitant care	#11d	Relevant concomitant care and interventions that are permitted or prohibited during the trial	11-12
Outcomes	#12	Primary, secondary, and other outcomes, including the specific measurement variable (eg, systolic blood pressure), analysis metric (eg, change from baseline, final value, time to event), method of aggregation (eg, median, proportion), and time point for each outcome. Explanation of the clinical relevance of chosen efficacy and harm outcomes is strongly recommended	13-15
Participant timeline	#13	Time schedule of enrolment, interventions (including any run-ins and washouts), assessments, and visits for participants. A schematic diagram is highly recommended (see Figure)	Figure 1, Table 1
Sample size	#14	Estimated number of participants needed to achieve study objectives and how it was determined, including clinical and statistical assumptions supporting any sample size calculations	16-18
Recruitment	#15	Strategies for achieving adequate participant enrolment to reach target sample size	18-19
**Methods: Assignment of interventions (for controlled trials)**			
Allocation: sequence generation	#16a	Method of generating the allocation sequence (eg, computer-generated random numbers), and list of any factors for stratification. To reduce predictability of a	15-16

For peer review only

		random sequence, details of any planned restriction (eg, blocking) should be provided in a separate document that is unavailable to those who enrol participants or assign interventions	
Allocation concealment mechanism	#16b	Mechanism of implementing the allocation sequence (eg, central telephone; sequentially numbered, opaque, sealed envelopes), describing any steps to conceal the sequence until interventions are assigned	15-16
Allocation: implementation	#16c	Who will generate the allocation sequence, who will enrol participants, and who will assign participants to interventions	15-16
Blinding (masking)	#17a	Who will be blinded after assignment to interventions (eg, trial participants, care providers, outcome assessors, data analysts), and how	15-16
Blinding (masking): emergency unblinding	#17b	If blinded, circumstances under which unblinding is permissible, and procedure for revealing a participant's allocated intervention during the trial	16
**Methods: Data collection, management, and analysis**			
Data collection plan	#18a	Plans for assessment and collection of outcome, baseline, and other trial data, including any related processes to promote data quality (eg, duplicate measurements, training of assessors) and a description of study instruments (eg, questionnaires, laboratory tests) along with their reliability and validity, if known. Reference to where data collection forms can be found, if not in the protocol	16-18,
Data collection plan: retention	#18b	Plans to promote participant retention and complete follow-up, including list of any outcome data to be collected for participants who discontinue or deviate from intervention protocols	18-19
Data management	#19	Plans for data entry, coding, security, and storage,	24

For peer review only

		including any related processes to promote data quality (eg, double data entry; range checks for data values). Reference to where details of data management procedures can be found, if not in the protocol	
Statistics: outcomes	#20a	Statistical methods for analysing primary and secondary outcomes. Reference to where other details of the statistical analysis plan can be found, if not in the protocol	16-18
Statistics: additional analyses	#20b	Methods for any additional analyses (eg, subgroup and adjusted analyses)	17-18
Statistics: analysis population and missing data	#20c	Definition of analysis population relating to protocol non-adherence (eg, as randomised analysis), and any statistical methods to handle missing data (eg, multiple imputation)	17-18
**Methods: Monitoring**			
Data monitoring: formal committee	#21a	Composition of data monitoring committee (DMC); summary of its role and reporting structure; statement of whether it is independent from the sponsor and competing interests; and reference to where further details about its charter can be found, if not in the protocol. Alternatively, an explanation of why a DMC is not needed	17, 19-21
Data monitoring: interim analysis	#21b	Description of any interim analyses and stopping guidelines, including who will have access to these interim results and make the final decision to terminate the trial	19-21
Harms	#22	Plans for collecting, assessing, reporting, and managing solicited and spontaneously reported adverse events and other unintended effects of trial interventions or trial conduct	19-21
Auditing	#23	Frequency and procedures for auditing trial conduct, if any, and whether the process will be independent from investigators and the sponsor	19-21

For peer review only

Ethics and dissemination

Research ethics approval	#24	Plans for seeking research ethics committee / institutional review board (REC / IRB) approval	22
Protocol amendments	#25	Plans for communicating important protocol modifications (eg, changes to eligibility criteria, outcomes, analyses) to relevant parties (eg, investigators, REC / IRBs, trial participants, trial registries, journals, regulators)	22
Consent or assent	#26a	Who will obtain informed consent or assent from potential trial participants or authorised surrogates, and how (see Item 32)	23
Consent or assent: ancillary studies	#26b	Additional consent provisions for collection and use of participant data and biological specimens in ancillary studies, if applicable	NA
Confidentiality	#27	How personal information about potential and enrolled participants will be collected, shared, and maintained in order to protect confidentiality before, during, and after the trial	24
Declaration of interests	#28	Financial and other competing interests for principal investigators for the overall trial and each study site	34
Data access	#29	Statement of who will have access to the final trial dataset, and disclosure of contractual agreements that limit such access for investigators	34
Ancillary and post trial care	#30	Provisions, if any, for ancillary and post-trial care, and for compensation to those who suffer harm from trial participation	19,22
Dissemination policy: trial results	#31a	Plans for investigators and sponsor to communicate trial results to participants, healthcare professionals, the public, and other relevant groups (eg, via publication, reporting in results databases, or other data sharing arrangements), including any publication restrictions	21,22
Dissemination policy:	#31b	Authorship eligibility guidelines and any intended use of	25

For peer review only

authorship		professional writers	
Dissemination policy: reproducible research	#31c	Plans, if any, for granting public access to the full protocol, participant-level dataset, and statistical code	34

Appendices

Informed consent materials	#32	Model consent form and other related documentation given to participants and authorised surrogates	Appendix 1
Biological specimens	#33	Plans for collection, laboratory evaluation, and storage of biological specimens for genetic or molecular analysis in the current trial and for future use in ancillary studies, if applicable	NA

For peer review only

RCT Adult with Capacity Information Sheet

Version 5.0, 25 May 2018

DexEnceph: A study of dexamethasone in adults with Herpes Simplex Virus (HSV) encephalitis
Brain Infections Group, University of Liverpool

We understand this is a difficult and stressful time for you, so we firstly want to thank you for taking the time to read this leaflet.

You are being invited to take part in a research study on HSV encephalitis. This condition is extremely rare and is probably something you had never heard about before. This is why a team member will go through this leaflet with you, explaining what taking part in the study would involve and answering any questions.

Important things you need to know

- This is a study for patients with encephalitis (swelling of the brain) caused by a virus called herpes simplex virus (HSV).
- Encephalitis can make you confused, drowsy, behave out of character, affect your sleep and memory, change your mood or may cause you to have fits.
- We want to find out if reducing the swelling with a drug called dexamethasone is of benefit to patient's memory in the longer term.
- In the study there will be two groups of patients, one that receives dexamethasone and one that does not.
- If you are in the group that receives dexamethasone this will be for 4 days in hospital.
- Both groups will have the same investigations to see if dexamethasone has been of benefit.
- Dexamethasone is a commonly used drug in brain swelling and many other conditions. Like all medicines, dexamethasone has side-effects. We will explain what these can be later.

We would like to invite you to take part in a research study

- Before you decide to take part it is important you know why the research is being done and what it will involve.
- You can discuss with family, friends and clinical staff before making a decision.
- You are free to decide whether you would like to take part.
- If you choose to take part and then decide you no longer want to be involved you can stop taking part without giving a reason. Your care will not be affected.
- Please let us know if there is anything in this leaflet that is not clear or if you would like more information. A member of our team will answer your questions.
- If you decide to take part we will offer you a copy of this form and ask you to sign a consent form.

For peer review only - http://bmjopen.bmj.com/site/about/guidelines.xhtml

HSV encephalitis

1. What is HSV encephalitis?

Encephalitis means swelling of the brain and has many different causes. It is often caused by a virus. Herpes Simplex Virus (HSV) is the most common virus that causes encephalitis in the UK.

HSV encephalitis is very rare. It is diagnosed by finding the virus in fluid around the brain and spinal cord. This fluid is called CSF (cerebrospinal fluid). The CSF is obtained by the doctor who performs a lumbar puncture (LP).

HSV encephalitis is treated with the drug aciclovir. Despite treatment, some people are left with significant loss of memory. About 2 out of every 3 people will have memory difficulties long term.

The study

2. Why are we doing this study?

We know dexamethasone can reduce swelling. Reduction in swelling of the brain may improve the recovery of patients with HSV encephalitis.

This study, called DexEnceph, will allow us to compare the recovery of patients that received dexamethasone and those that did not.

3. Why have I been invited to take part?

There are two reasons why you may have been invited to take part:

A. Your doctors have diagnosed you with having HSV encephalitis.

OR

B. You may have been invited to take part before the diagnosis is made. This is because your doctors think there is a chance you may have HSV encephalitis. This will mean you have more time to think about taking part.

4. What will happen to me during the study?

All patients in the study will receive aciclovir. This is standard treatment for HSV encephalitis.

In addition, if you decide to take part in the study, you may be offered a short course of dexamethasone. This will be decided at random by a computer. This is to be fair, so neither you, your doctor, nor the research team, can choose whether you receive dexamethasone or not. Half of the people in the study will receive dexamethasone and half will not.

If you receive dexamethasone this will be 4 times a day for 4 days. It is given in a line you already have for clinical care.

What taking part involves

5. What tests are done if I take part?

When you sign up to the study:
Blood sample
CSF sample
MRI scan

↓

After 2 weeks:
MRI scan
Blood sample
CSF sample

↓

After 1 month (or when you are discharged from hospital):
Review of activities daily living
Short memory test only

↓

After 6 months:
Memory test
MRI scan
Blood sample
Review of activities daily living
Health questionnaires

↓

After 18 months:
Memory test
MRI scan
Review of activities daily living
Health questionnaires

All the tests done when you sign up to the study and the CSF tests after 2 weeks will

For peer review only - http://bmjopen.bmj.com/site/about/guidelines.xhtml

be done as part of your care whether you take part in the study or not.

6. What do the memory tests involve?

These tests are the most important in the study as they will help us find out if dexamethasone improves memory problems from HSV encephalitis. These tests are sometimes called Neuropsychology tests. They are completed 6 and 18 months after the illness.

The key part of this test takes about 35 minutes. If you are not too tired we can continue with further tests that will provide useful information. These can take up to 2 hours.

They are not pass or fail tests. They provide information about your memory and thinking processes.

They can be done in one day or divided over a few short visits. If you have left hospital we can travel to see you in a convenient place for you. The test will be arranged on a day(s) which suits you.

The results can be added to your hospital notes for future reference if you wish or kept confidential within the trial.

7. What does the MRI scan involve?

As part of your care your doctor will organise an MRI scan when you are in hospital. If you take part in the study we will ask you to have another 3 scans later on.

MRI scans allow us to assess if the brain has been affected by the infection and, if so, which parts.

Each scan takes about 20 minutes. The scan can be noisy but you will be offered headphones.

The extra 3 scans are planned for:

- 2 weeks after the first one (when you are still in hospital)
- After 6 months
- After 18 months

The scans will be done at a hospital near you. We will reimburse mileage or public transport costs for any research visits.

We will check with you that you are still happy to have the scan each time. Sometimes scans may find something not related to this illness. If this happens the doctors looking at the scans will tell your own hospital doctors who will look into this further.

None of the research scans are compulsory so if you do not wish to have them you can still be part of the study.

8. Are there risks to having an MRI scan?

There are no known risks from an MRI scan. They do not use radiation. MRI scans are done routinely in patients with HSV encephalitis.

Because MRI scans use strong magnets you will not have the scan if you have any metal implants or fragments in your body.

Where you lie is quite enclosed and some people may find this unsettling. If you have a fear of confined spaces you should discuss this with your doctor before you go for the scan.

If you think you may be pregnant let your local research team know. We will not ask pregnant women to have MRI scans due to possible risks to the foetus.

9. What samples are collected? What does this involve?

We will collect blood and CSF samples during the study.

All patients with HSV encephalitis need a lumbar puncture (LP) when they come to hospital to find out why they are unwell. The doctor uses a small needle to take a sample from the lower part of the back. This is repeated after 2 weeks of treatment to see if all the virus has gone. Both lumbar punctures are part of the standard care in all patients with this condition.

For peer review only

For peer review only - http://bmjopen.bmj.com/site/about/guidelines.xhtml

UNIVERSITY OF LIVERPOOL NHS National Institute for Health Research

We will take a little extra fluid at this time for the research tests. The amount of fluid we ask for each time is about 1 teaspoon, 5.5mls.

If you have already had a lumbar puncture before being told about the study, we will take stored CSF that is leftover for research tests.

Blood tests are requested at 3 different times spread over 6 months. We take between 1 to 4 teaspoons of blood, this is 5 to 23mls.

With these blood and CSF tests we will be able to better understand how the infection affects your body and how the body tries to defend itself against it.

10. What will happen to the samples that are collected? Will any genetic tests be done?

All samples will be taken at your hospital and then transported to the University of Liverpool or other laboratories supporting the study. The samples will not have any of your personal information written on them. In the University they will be stored in a secure building.

There is an option for the blood and CSF collected to have tests looking at DNA. DNA is found in all cells of the body and contains the genetic information for the working of all human beings. This study collects DNA samples to find out why some people get HSV encephalitis and others do not, and why some people have severe problems due to HSV and others do not. The information we learn from DNA may benefit others with this condition in the future but will not influence your treatment or your future health.

Some of your samples may be left over. We will ask you if they can be used for this and future studies run by the University of Liverpool.

11. How do you review activities of daily living?

We will find out how the illness has affected your day-to-day life.

The research team will look at your hospital notes. They may also talk to you and, if you choose, your relatives. This will happen when you are in hospital and when you have gone home.

We will compare patients who received dexamethasone to those that did not and see if it made a difference.

12. What are the health questionnaires?

Two questionnaires will be sent through the post. They will ask you your views about your health and quality of life. Please send them back in a pre-paid envelope.

Dexamethasone

13. What are the side effects of dexamethasone?

Dexamethasone is used widely in patients and the side-effects are well known as this medicine has been prescribed for a long time. A short course of dexamethasone will be prescribed in this study. Side effects are less common when dexamethasone is given for shorter periods.

It is important you know about the possible side-effects before you decide to take part. These are:

- Stomach pain, indigestion, having more appetite than usual, feeling or being sick.
- Feeling tired or fatigued
- Mood and behaviour changes, especially at the beginning.
- Higher blood sugars.

Other possible risks can include:

- Stomach ulcers and bleeding of ulcers.
- Decreased response to infections.

For peer review only

For peer review only - http://bmjopen.bmj.com/site/about/guidelines.xhtml

You will be in hospital when you take dexamethasone so you can tell your doctors immediately if you have any problem.

If you suffer side effects you or your doctor can decide to stop the dexamethasone at any point.

Dexamethasone is prescribed to women who are pregnant or breast feeding as there are no known risks to the foetus.

Other things to consider

14. Do I have to take part?

No, taking part is voluntary. If you agree, we will ask you to sign a consent form.

If you agree to take part you are free to change your mind at any time, without giving a reason. You may decide to have only some tests in the study without having to drop out of the study altogether. This will not affect the standard of care you receive.

If you withdraw from the study we will stop collecting data. We will ask you if we can use the information and samples we have gathered up to the point that you withdraw.

15. What happens if there is a problem?

If you have any concerns about any part of this study, please speak with your hospital doctor (consultant) or one of your research team.

If you remain unhappy and wish to complain formally you can do this using the NHS Complaints Procedure. You can get information on how to do this from the Patients Advice Liaison department (PALS) in your hospital.

If you suffer harm from taking part in this study, there are no special compensation arrangements. If harm occurs to you and it is due to someone's negligence, you may have grounds for legal action for compensation against the NHS hospital where you are being treated but you may have to pay your legal costs.

16. Who will know I have taken part in this study?

Only people in your clinical care team and people involved in the study will have access to personal data. With your consent we will tell your GP that you are taking part.

All information collected about you during this study will be confidential and anonymised. It will be handled, stored and destroyed in accordance with the General Data Protection Regulation.

University of Liverpool is the sponsor for this study based in the United Kingdom. We will be using information from you and your medical records in order to undertake this study and will act as the data controller for this study. This means that we are responsible for looking after your information and using it properly. University of Liverpool will keep identifiable information about you for 15 years after the study has finished

Your rights to access, change or move your information are limited, as we need to manage your information in specific ways in order for the research to be reliable and accurate. If you withdraw from the study, we will keep the information about you that we have already obtained. To safeguard your rights, we will use the minimum personally-identifiable information possible.

You can find out more about how we use your information http://www.dexenceph.org.uk/. Our Data Protection Officer is Victoria Heath and you can contact them at V.Heath@liverpool.ac.uk.

Benefits and risks

17. What are the benefits of taking part?

You may benefit from receiving dexamethasone, however we will not know this until the end of the study. You

For peer review only - http://bmjopen.bmj.com/site/about/guidelines.xhtml

may also benefit from the increased monitoring of having extra scans and memory tests.

The information we get from this study may benefit patients in the future.

18. What are the possible disadvantages and risks of taking part?

The disadvantage in taking part in this study may be the risk of having the side-effects of dexamethasone listed in question 13 (this will not be the case if you are in the group that does not have dexamethasone).

There is the inconvenience of having the dexamethasone through the drip when you are in hospital. Once you leave hospital there is the inconvenience of travelling to hospital for 2 scans, having the memory tests and completing questionnaires.

Contact details

If you have any questions about this study, then please contact the study team members:

Principal Investigator (Doctor leading this study in your hospital):
Name: ______________________________

____

Telephone:__________________________

Research Nurse:
Name: ______________________________

____

Telephone:__________________________

Name of your Hospital:_______________

____

Further information

This study is being run at your hospital and many other NHS hospitals throughout the UK. It aims to recruit 90 patients over 4 years.

It is organised by the University of Liverpool and is funded by the National Institute for Health Research (NIHR), the public body in charge of research in the UK.

Our study team includes The Encephalitis Society, a charity that supports patients and families (www.encephalitis.info).

The study has been reviewed for scientific content by expert members of NIHR. The National Research Ethics Service Committee Liverpool Central has reviewed the study and given approval for it to take place.

For peer review only

For peer review only - http://bmjopen.bmj.com/site/about/guidelines.xhtml

RCT Adult with Capacity Consent Form
Version 5.0, Dated: 25/May/2018
EudraCT Number: 2015-001609-16

Centre Name: Centre Code:
Name of Principal Investigator:
Study Number:

For peer review only

Please complete this form. When completed give one copy to the participant to keep, send one copy to CTU [fax/encrypted email/post], and keep one in the participant's medical notes. Please put the original in the site file.

**For patient: once you have understood each statement please initial the YES OR NO box**	**YES**	**NO**
1. I confirm I have read and understand the Information Leaflet (dated DD/MMM/YYYY) for the above study, and have had the opportunity to ask questions and have these answered satisfactorily.	INITIAL IF YES	INITIAL IF NO
2. I agree to take part in this study.	INITIAL IF YES	INITIAL IF NO
3. I understand that my participation is voluntary and that I am free to withdraw from the study at any time without giving a reason and without my care or legal rights being affected.	INITIAL IF YES	INITIAL IF NO
4. I agree for my consent form and contact details to be passed to the University of Liverpool for the administration of the study.	INITIAL IF YES	INITIAL IF NO
5. I understand that relevant sections of my medical notes and any data collected during the study may be looked at by authorised individuals from the research and clinical team and Regulatory Authorities where it is relevant to my taking part in this research.<br>I give permission for these individuals to have access to my records.	INITIAL IF YES	INITIAL IF NO
6. I agree for genetic tests to be done on blood and CSF collected. I understand these genetic tests will not be of any individual significance to me.	INITIAL IF YES	INITIAL IF NO

For peer review only - http://bmjopen.bmj.com/site/about/guidelines.xhtml

University of Liverpool | NHS National Institute for Health Research

7. I agree to have MRI scans as part of the trial.	INITIAL IF YES	INITIAL IF NO
8. I agree for my GP and hospital doctors to be informed if the scan picks up something unexpected.	INITIAL IF YES	INITIAL IF NO
9. I agree to gift the remainder of any blood or CSF sample to the University of Liverpool where it will be stored for use in future research. This may include genetic tests.	INITIAL IF YES	INITIAL IF NO
10. I agree to any images or scans that are taken to be used for teaching, education and publication (in scientific journals, books or internet).	INITIAL IF YES	INITIAL IF NO
11. I agree for my GP to be informed I am taking part in this study.	INITIAL IF YES	INITIAL IF NO

Name of Participant (Please print)	Signature	Date (DD/MM/YYYY)
Researcher*	Signature	Date (DD/MM/YYYY)

*** Important:** Prior to signing please ensure local research contact details are complete on page 6.

<u>Information to Research Team:</u>
Once a Consent Form has been signed, please copy three times: One for the participant, one to file in the medical notes and fax/post/encrypted email one to CTU. Please place original in the site file.

Please fax/encrypt email/post this consent form to CTU **separately** to other anonymised trial documents (e.g. CRF).

For peer review only

For peer review only - http://bmjopen.bmj.com/site/about/guidelines.xhtml
